# Lipid based nutrient supplements (LNS) for treatment of children (6 months to 59 months) with moderate acute malnutrition (MAM): A systematic review

**DOI:** 10.1371/journal.pone.0182096

**Published:** 2017-09-21

**Authors:** Tarun Gera, Juan Pablo Pena-Rosas, Evelyn Boy-Mena, Harshpal S. Sachdev

**Affiliations:** 1 Department of Pediatrics, SL Jain Hospital, Delhi, India; 2 Evidence and Programme Guidance, Department of Nutrition for Health and Development, World Health Organization, Geneva, Switzerland; 3 Department of Pediatrics and Clinical Epidemiology, Sitaram Bhartia Institute of Science and Research, New Delhi, India; Universidade de Sao Paulo, BRAZIL

## Abstract

**Background:**

Moderate acute malnutrition is a major public health problem affecting children from low- and middle-income countries. Lipid nutrient supplements have been proposed as a nutritional intervention for its treatment.

**Objectives:**

To evaluate the effectiveness and safety of LNS for the treatment of MAM in infants and children 6 to 59 months of age.

**Study design:**

Systematic review of randomized-controlled trials and controlled before-after studies.

**Results:**

Data from nine trials showed that use of LNS, in comparison to specially formulated foods, improved the recovery rate (RR 1.08; 95% CI 1.02–1.14, 8 RCTs, 8934 participants, low quality evidence); decreased the chances of no recovery (RR 0.70; 95% CI 0.58–0.85, 7 RCTs, 8364 participants, low quality evidence) and the risk of deterioration into severe acute malnutrition (RR 0.87; 95% CI 0.73–1.03, 6 RCTs, 6788 participants, low quality evidence). There was little impact on mortality (RR 0.94, 95% CI 0.54–1.52, 8 RCTs, 8364 participants, very-low- quality evidence) or default rate (RR 1.32; 95% CI 0.73–2.4, 7 studies, 7570 participants, low quality evidence). There was improvement in weight gain, weight-for-height z-scores, height-for-age z-scores and mid-upper arm circumference. Subset analyses suggested higher recovery rates with greater amount of calories provided and with ready-to-use therapeutic foods, in comparison to ready-to-use supplementary foods.

One study comparing LNS with nutritional counselling (very low quality evidence) showed higher chance of recovery, lower risk of deteriorating into severe acute malnutrition and lower default rate, with no impact on mortality, and no recovery.

**Conclusions:**

Evidence restricted to the African regions suggests that LNS may be slightly more effective than specially formulated fortified foods or nutritional counselling in recovery from MAM, lowering the risk of deterioration into SAM, and improving weight gain with little impact on mortality or default rate.

## Background

Moderate acute malnutrition in children is defined by the World Health Organization (WHO) and the United Nations Children's Fund (UNICEF) as a low weight-for-height between -2 and -3 z-scores of the median WHO growth standards, without edema [1], or an arm circumference between 115 and125 mm, without edema in children aged 6 to 59 months [2]. Children affected by moderate acute malnutrition (also sometimes referred to as moderate wasting) comprise 10% under five population from low and middle income countries, or around 33 million, mostly from South Asia and Africa [3–6]. Malnutrition is generally multifactorial, caused by a vicious cycle between infections and malnutrition, and amplified by food insecurity and poverty in the low and middle income countries. The problem is exacerbated by natural and manmade disasters, like war, drought and famines. [6, 7].

Children with moderate acute malnutrition are at a higher risk for morbidities and mortality. A greatly reduced muscle mass is characteristic of acute malnutrition, and this increases the risk of death during infections [8]. Acute malnutrition has been estimated to be responsible for almost 12.6% of deaths in children under 5 years of age. Of this severe acute malnutrition contributes to 7.4% of the under-5 mortalityHowever, with larger absolute numbers, the population-attributable risk of malnutrition to mortality is much higher [9, 10]. In addition, moderate acute malnutrition is likely to progress to severe acute malnutrition, if left untreated. Both prevention and treatment of moderate acute malnutrition are, therefore, likely to improve under-five survival globally.

The treatment of moderate acute malnutrition till now has been limited to distribution of supplementary foods [11]. These home-based diet and products like blended flours have been inefficacious due to high cost, low accessibility and a nutritional profile not designed to promote recovery (low fat and high protein content) [12, 13]. Lipid-based nutrient supplements have been effective in promoting rapid weight gain in severe acute malnutrition. Given a similar pathophysiology they were used with some success in treating children with moderate acute malnutrition [14]. Lipid-based nutrient supplements refer to products where the majority of energy is provided by lipids. and include energy, protein, essential fatty acids, and micronutrients[15, 16]. Three types of lipid-based nutrient supplements are currently being used in children: (i) *Ready-to-use therapeutic foods*: These have been traditionally used to treat severe acute malnutrition. Since they are supposed to provide all the energy requirements of the child, they are given in large doses (200–300 g/day) [17, 18]. Their composition consists of a staple food like a cereal as the base ingredient, with a protein source and a source of energy to increase the amount of calories provided [19]. They also provide micronutrients. The other properties of these foods are aimed at increasing their shelf life and lowering the risk of contamination, viz, they should not require water for constitution, not require refrigeration, and be free from contamination. [20]. Initially, the ready-to-use therapeutic foods were made from peanut, milk, oil and micronutrients (for example, Plumpy Nut® and BP 100). A number of other formulations have since been tried with alternative sources of proteinslike a combination of cereals and pulses, soy flour, soy protein concentrates or a cheaper protein of animal source, such as whey protein concentrates [19,21]; (ii) *Medium quantity lipid-based nutrient supplements or ready-to-use supplementary foods* were designed either for treatment of moderate acute malnutrition [22], or for prevention of seasonal wasting [23] or stunting [23, 24]. They provide 50–100% of the daily energy requirements, excluding that from breastfeeding (250 to 500 kcal per day) [25, 26]. They are usually required to be given in amounts of 50 to100 g/day.; and (iii) *Low quantity lipid-based nutrient supplements* have been used to prevent wasting and stunting in infants and children. They provide less than 50% of the daily energy requirements and are, therefore, used in lower doses (20 to 50 g/day). Examples of these products include Nutributter® and 'fortified' local foods [27].

The objective of nutritional therapy in acute malnutrition is to provide macro- and micronutrients in relatively large quantities to enable fast recovery [28, 29]. Since infants and children have small body sizes that limit the quantity of food that can be consumed at one time, the supplement has to be energy dense and given at frequent intervals [30, 31]. Lipid-based nutrient supplements satisfy these basic criteria [16, 28].

In the absence of standard guidelines for the management of uncomplicated moderate acute malnutrition and variety of products available, it is important to systematically evaluate the effectiveness of the various approaches and products available. One recent Cochrane Review has evaluated the benefits and safety of specially formulated foods in moderate acute malnutrition [32]. However, additional trials on this subject have become available or are nearing completion. Furthermore, several concerns about the use of lipid-based nutrient supplements in severe and moderate acute malnutrition have generated considerable controversy. The important articulated concerns [15, 33] include: (i) the potential to alter feeding habits (displace breast milk or diverse local diets); (ii) the adverse effects of excessive lipid intake on body composition; (iii) whether and how imported or processed foods should be used to prevent or treat malnutrition; (iv) inappropriate commercial promotion of infant foods; (v) the cost of the products, which raises concerns both about sustainability and trade-offs/opportunity costs; (vi) tendency to monopolize by a handful of manufacturers able to put in place strict quality control measures; and (vii) potential environmental impact. It is also unclear if there are differences in effectiveness and safety of the intervention according to the population characteristics like breastfeeding status, HIV status, age group and stunting. This review intends to update the earlier evidence, particularly in relation to some controversial and unexplored aspects listed above. The stated objective is to evaluate the effectiveness and safety of lipid-based nutrient supplements for the treatment of moderate acute malnutrition in infants and children between 6 and 59 months of age.

## Methods

### Criteria for considering studies for this review

#### Types of studies

Randomized controlled trials, randomized at the level of the individual or cluster, were included. In anticipation of non-availability of sufficient trials of this nature, we planned to include data from the following additional study designs: (i) Non- randomized trials at the level of the individual or cluster. To be eligible, such trials should have a concurrent comparison group (as defined later), preferably with adjustment for baseline characteristics and confounders; (ii) Controlled before-after studies where allocation to the different comparison groups was not made by the investigators. Outcomes of interest should have been measured in both intervention and control groups before the lipid-based nutrient supplements intervention was introduced, and again after the intervention had been introduced.

We planned to include cluster-randomized trials, non-randomized cluster trials, and controlled before-after studies with at least two intervention sites and two control sites.

Eligible studies were included irrespective of the date, language or publication status.

#### Types of participants

Infants and children between the ages of 6 and 59 months with moderate acute malnutrition, defined as a weight-for-height between ≥ -3 SD (Z score) and < -2 SD (Z score) of WHO standard or a mid-upper arm circumference ≥ 115 mm and < 125 mm. The studies were included irrespective of setting or disease status of the study population.

#### Types of interventions

Intervention: Lipid-based nutrient supplementation, as defined by the authors was considered the intervention. The supplements could have been produced centrally or locally and prepared with either a commercial or non-commercial intent. We included studies using any variations in the calories provided, milk content, type of macronutrients like type of oil (e.g. omega 3 fatty acids) or protein quality, maximum duration of supplementation and the definition of recovery.

Comparison: The comparison groups comprised either of the following: (i) Lipid-based nutrient supplements vs. specially formulated supplementary micronutrient fortified foods (e.g. fortified cereal-legume blends); (ii) Lipid-based nutrient supplements vs. no supplementary foods or no intervention; (iii) Lipid-based nutrient supplements vs. multiple micronutrient powders for point-of-use fortification of foods (improved adequacy of local diet); and(iv) Lipid-based nutrient supplements vs. counselling on feeding best possible local diet—home-based foods.

Studies employing simultaneous co-interventions like health education and/or drugs (e.g. deworming or antimalarials) were included if the only difference between the intervention and comparison arms pertained to the provision of lipid-based nutrient supplements.

#### Types of outcome measures

Primary outcomes included (i) Proportion of children recovered (author defined); (ii) Duration of recovery (> 1 month; 1 to 2 months; > 2 months); (iii) Mortality; (iv) Weight; (v) Height; (vi) Weight-for-height z-score; (vii) Mid-upper arm circumference; (viii) Height-for-age; and (ix) Relapse (%, readmission into treatment programme).

Secondary outcomes included (i) Default rate (%, author defined); (ii) Morbidity (author defined; incidence of diarrhoea, acute respiratory illness, others); (iii) Haemoglobin (g/dL); (iv) Micronutrient status (e.g. vitamin A, iron); (v) Any adverse effects (author defined; e.g., allergic reactions as diagnosed by clinical assessment, including atopic dermatitis, urticaria, oedema (oral), ophthalmic pruritus, allergic rhinitis, asthma, anaphylaxis); (vi) Change in body composition; (vii) Cardio-metabolic risk factors, including blood pressure, glucose tolerance, and serum lipids; (viii) Change in infant and young child feeding practices; (ix) Environmental impact of lipid-based nutrient supplementation for example, waste from packaging; (x) Intra-household sharing of the provided supplement; and (xi) Cost-effectiveness.

### Search methods for identification of studies

We searched the following electronic databases for primary studies, using appropriate keywords, with last search date of June 4, 2016:

- Medline
- Web of Science
- The Cochrane Controlled Trials Register
- EMBASE (Ovid)
- LILACS
- Popline
- African Index Medicus
- CINAHL Plus (EBSCO)
- ClinicalTrial.gov
- WHO International Clinical Trials Registry Platform (WHO-ICTRP)

The detailed search strategies are reported in S1 Appendix.

Reference lists of all included papers and relevant reviews were scanned to identify citations that could have been missed in the primary search. Authors of other relevant reviews in the field and those identified from the primary search were contacted for information regarding relevant studies, of which they were aware.

### Data collection and analysis

#### Selection of studies

The title and abstract of the studies identified in the search were scanned independently by two authors (TG and HSS) to exclude literature that clearly did not meet the inclusion criteria. With the remainder, we examined the full text articles against the eligibility criteria using a structured form. Any uncertainty about inclusion was resolved through discussion and by consensus between all authors.

#### Data extraction and management

Two authors (TG and HSS) independently extracted relevant data including details of methods, participants, setting, context, interventions, outcomes and results, publications and investigators using a structured form. We contacted the study authors where reported information was unclear or contradictory, or where important data were missing.The abstracted data on the forms was independently evaluated by the other author. Any discrepancies were resolved through consensus and mutual discussion.

#### Assessment of risk of bias in included studies

Two review authors (TG and HSS) independently assessed the risk of bias for each controlled trial using the criteria outlined in the Cochrane Handbook for Systematic Reviews of Interventions [34].

For randomized controlled trials, non-randomized controlled trials and controlled before-after studies these criteria included:

1. sequence generation (selection bias);
2. allocation sequence concealment (selection bias);
3. blinding of participants and personnel (performance bias);
4. blinding of outcome assessment (detection bias);
5. incomplete outcome data (attrition bias); and
6. selective outcome reporting (reporting bias).

For cluster- randomized trials, particular biases to consider included

1. recruitment bias;
2. baseline imbalance;
3. loss of clusters;
4. incorrect analysis; and
5. comparability with individually randomized trials [34].

The judgment for each entry involved assessing the risk of bias as ‘low risk’, as ‘high risk' or as ‘unclear risk’, with the last category indicating either lack of information or uncertainty over the potential for bias. Plots of ‘risk of bias’ assessments were created in RevMan software. Any disagreements were resolved through mutual discussion or group discussion, if required.

#### Measures of treatment effect

Risk ratio (RR) estimations with 95% confidence intervals (CI) were used for binary outcomes. Data on continuous outcomes was analysed using either mean differences (MD) or standardized mean differences (SMD) if continuous outcomes were measured with similar, but not identical, instruments across studies. In order to maximize the data input for the pooled outcome measures, we utilized the post-intervention values (means and standard deviations (SDs) in preference to the changes from baseline. We used Review Manager 5.3 to manage the data and conduct the analysis. All results are presented with 95% confidence intervals.

#### Unit of analysis issues

In factorial trials and in multi-arm designs yielding two or more intervention groups (different lipid-based nutrient supplements used) and a single control group, the data in the intervention groups, including the variation in the intervention characteristic, was pooled and compared against the single control group to prevent unit-of-analysis error. Ackatia-Armah (C) [35–38] had one intervention arm receiving lipid-based nutrient supplements and three control arms receiving different types of cereal legume blends. The data from these three control groups was pooled for purpose of analyses. LaGrone [39, 40] and Matilsky [22] compared two different types of ready-to-use therapeutic foods with cereal legume blend (corn soy blend plus plus). Here the data in the two intervention groups was pooled to avoid unit of analysis issues.Nikema et al. [41] had a multiarm design comparing the effect of lipid-based nutrient supplements (ready-to-use supplementary foods) with a cereal legume blend and nutritional counselling. These were treated as separate comparisons and are presented as such; i.e., lipid-based nutrient supplements versus specially formulated fortified food and lipid-based nutrient supplements versus nutritional counselling.

#### Handling of missing data

We preferred to use intention-to-treat analyses (author reported), if a per-protocol analysis was also reported. If the author had not reported an intention-to-treat analysis, we used the reported analysis but judged the study to be at risk of bias due to this criterion. In case of missing data, we contacted trial authors for information, wherever possible. Where this could not be done, or the authors did not respond, the missing values were imputed where feasible with the help of a statistician.

#### Assessment of heterogeneity

We intended to assess contextual heterogeneity on the basis of information collected on the context in which the intervention was implemented. We assessed for variability in the participants, interventions and outcomes studied to identify clinical heterogeneity, and for variability in study design to describe methodological diversity.

Statistical heterogeneity was identified by visual inspection of the Forest plots and the Chi square tests, and measured as recommended by the Cochrane Handbook for Systematic Reviews of Interventions (section 9.5.2) [34]. A rough guide used for interpretation was: (i) 0% to 40%: might not be important; (ii) 30% to 60%: may represent moderate heterogeneity; (iii) 50% to 90%: may represent substantial heterogeneity; and (iv) 75% to 100%: considerable heterogeneity.

In view of few included trials, a P value of 0.10 from the Chi2 test was used as cut off for statistical significance.

#### Assessment of reporting biases

The presence of reporting bias in the extracted data was evaluated quasi-statistically using a funnel plot [42]. Formal statistical tests for funnel plot asymmetry, namely the Begg and Egger’s methods [43, 44] were not conducted because of insufficient number of trials.

#### Data synthesis

We performed statistical analysis using the Review Manager software (RevMan 5.3). In concordance with the current recommendations [34], we conducted the meta-analysis of included randomized controlled trials and other trials separately. Pooled estimates (RR with 95% CIs) of the evaluated outcome measures were calculated by the generic inverse variance method. Results are not depicted as ‘not statistically significant’ or ‘nonsignificant’, but we have reported the CI together with the exact P value. We included studies with large variation with respect to populations, interventions, comparators, outcomes and settings. Thus, the true effect is likely to be related, but not the same for all studies. We therefore chose the random-effects model. If it was not possible to synthesise the data for some outcomes (e.g duration to recovery), we provided a narrative synthesis of the results.

The data was finally synthesised using a ‘summary of findings’ table that provided key information concerning the quality of evidence, the magnitude of effect of the interventions examined, and the sum of available data on all primary outcomes for a given comparison. For each primary outcome, quality assessment of the results was also carried out using the GRADE approach, which specifies four levels of quality (high, moderate, low and very low) where the highest quality rating is for a body of evidence based on randomized trials. Quality was assessed separately for each outcome.

#### Subgroup analysis and investigation of heterogeneity

We planned to explore differences in recovery by the following subgroup analysis, if data permitted: age of the study group (6 to 24 mo, > 2years), breastfeeding practises in under 2 population, dietary intake of the study population, exposure to HIV infection (yes/no). antiretroviral therapy for HIV infected population. setting in refugee or internally displaced camps (yes/no). living in an emergency affected country (WHO definition) and not in a camp (yes/ no), belongs to food insecure group (yes/no). height-for-age of the study population. treatment provided in inpatient or outpatient setting. dose (calories provided per kg per day) of the formulation. type of formulation (milk based or not). type of macronutrients provided and duration of intervention.

The available data, however, allowed us to conduct subgroup analyses only for age of study group, dose of formulation, type of formulation (ready-to-use therapeutic foods vs. ready-to-use supplementary foods) and duration of intervention.

#### Sensitivity analysis

We undertook sensitivity analyses with respect to the risk of bias parameters, namely: (i) sequence generation (selection bias), (ii) allocation sequence concealment (selection bias), (iii) blinding of participants and personnel (performance bias), (iv) blinding of outcome assessment (detection bias), (v) incomplete outcome data (attrition bias), (vii) selective outcome reporting (reporting bias) and (viii) other potential sources of bias.

The protocol of this review was registered at PROSPERO (CRD42016036458).

## Results

### Description of studies

#### Results of the search

**Fig 1:**
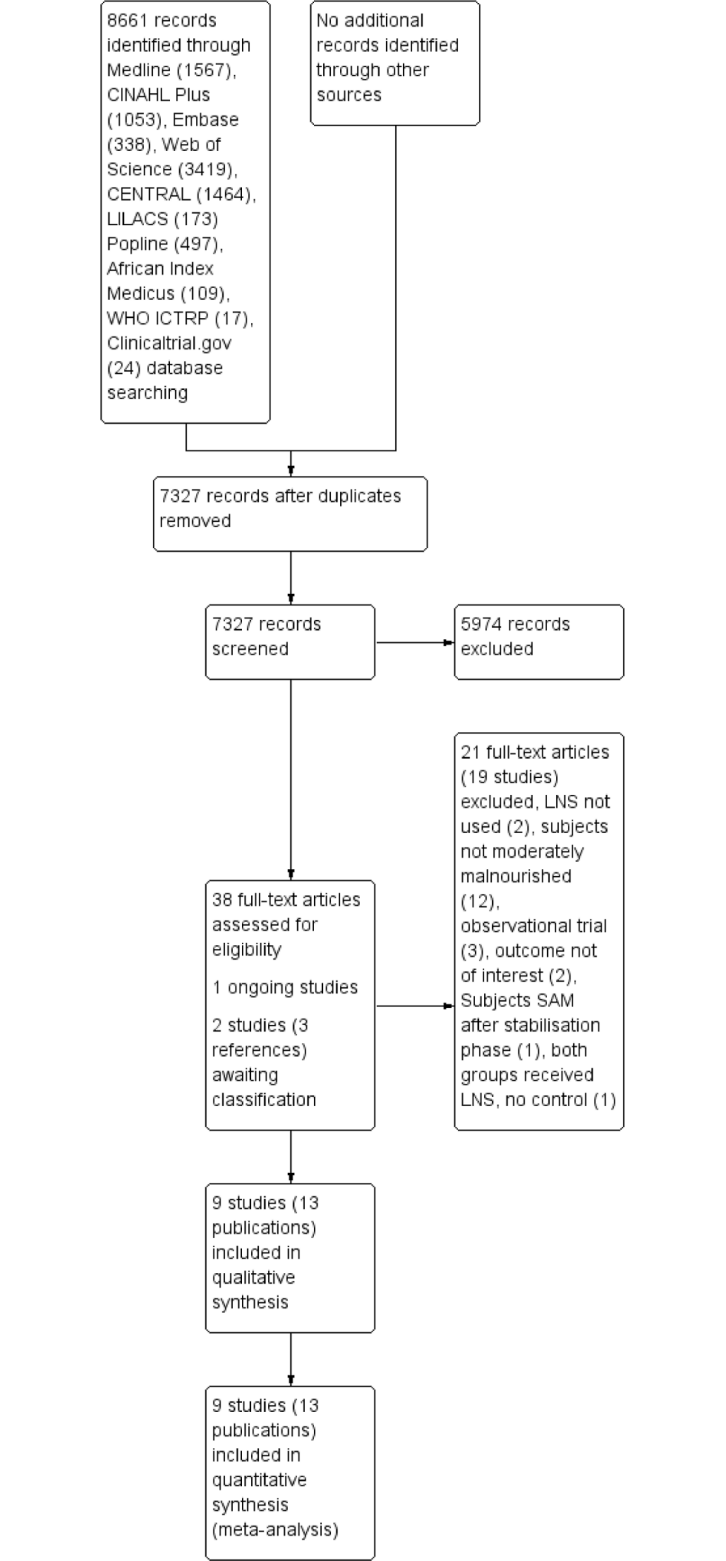
Study flow diagram.

The search results are summarised in Fig 1. We screened 8661 records, of which 38 were potentially eligible. Of these 13 publications (9 studies) were included in the final analyses [22, 35–41, 45–49] and 21 references (from 19 studies) [14, 17, 50–68] were excluded. One study is ongoing [69] and three references (from two studies) await classification [70–72] (S6 Table, S7 Table).

#### Included studies

A total of 9 trials, incorporating data on 9270 children with moderate acute malnutrition were included in the final analysis (Table 1). Four trials were cluster randomized controlled trials, four were randomized controlled trials and one was a non-randomized controlled trial. All the trials were conducted in Africa. None of the trials were conducted in an emergency/refugee setting. One trial was conducted in a hospital setting [45] while the rest were community-based trials. Only one trial included children younger than 2 years exclusively [41], while most included children between the ages of 6 months to 5 years. Authors used different definitions for recovery (Table 1; S2 Table). The intervention was ready-to-use supplementary foods in seven of the nine included trials, while the other two provided ready-to-use therapeutic foods. Three of the lipid-based nutrient supplements used were milk based. The comparator was specially formulated micronutrient fortified food in all of the nine included trials (Cereal Legume Blend in 8 and United Nations Feeding Programme Supplementations in one). One trial [41] also used nutritional counselling as a control arm which was analyzed separately. The duration of supplementation varied (from 8 to 16 weeks). Calories provided varied from 250 to 1200 kcal/day in 7 trials and from 40 to 75 kcal/kg/day in 2 trials. The important characteristics of populations, studies and interventions are summarized in Tables 1 and 2 and details of individual studies can be seen in the section S2 Table. S3 Table compares the PROGRESS-Plus equity categories in the included studies. A descriptive analysis of the PROGRESS-Plus factors that were reported is presented in S4 Table. None of the studies had reported directly on social determinants of health.

**Table 1:** Characteristics of populations from the included trials.

Study	Study design	Country	Age group	Setting	HIV status	Definition of moderate acute malnutrition	Definition of recovery
Ackatia-Armah 2015 (35–38)	Cluster randomized controlled trial	Mali, Africa	6–35 months	Community	Negative	(Weight-for-length z-score) < -2 and ≥ -3 of WHO Reference or mid-upper arm circumference < 12.5 cm and ≥ 11.5 cm or weight-for-length z-score < 80% and ≥ 70% of National Center for Health Statistics median or mid-upper arm circumference < 12.0 and ≥ 11.0 cm, without edema	Weight-for-length z-score ≥ -2.0 and mid-upper arm circumference ≥12.5 cm during at least 2 consecutive follow-up visits
**Delchevalerie 2015 (C)**	Cluster randomized controlled trial	Sierra Leone. Africa	6–59 months	Community	Not mentioned	Weight-for-height of the reference median 70 to79% without edema (Department of Health and Human Statistics, CDC, US, 2002)	Weight-for-height of the reference median > 85% for two consecutive weeks
**Karakochuk 2012 (C)**	Cluster randomized controlled trial	Ethiopia, Africa	6–60 months	Community	Not mentioned	Mid-upper arm circumference <135 mm and weight-for-height ≥ 70 to < 80% as per National Center for Health Statistics reference	2 consecutive measurements of weight-for-height > 85% within a 16-wk time period
**LaGrone 2012**	Randomized controlled trial	Malawi, Africa	6–60 months	Community	Projected HIV 0.2–2%	Weight-for-height z-score < -2 and > -3z of WHO Reference without bipedal edema	Weight-for-height z-score ≥ -2z
**Matilsky 2009**	Randomized controlled trial	Malawi, Africa	6–60 months	Community	Not mentioned (same as LaGrone 2012)	Weight-for-height z-score< -2 but ≥ -3 of WHO Reference	Weight-for-height z-score ≥ -2z
**Medoua 2016**	Randomized controlled trial	Cameroon, Africa	24–59 months	Community	Not mentioned	Weight-for-height z-score< -2 but ≥ -3 of WHO Reference	Weight-for-height z-score> -2
**Nackers 2010**	Randomized controlled trial	Niger, Africa	6–59 months^1^	Community	Not mentioned	Percent weight-for-height of the reference median from 70 to < 80% (National Center for Health Statistics reference) without oedema; mid-upper arm circumference ≥ 110 mm.	Weight-for-height of the reference median ≥ 85% for 2 consecutive weeks
**Nikiema 2014 (C)**	Cluster randomized controlled trial	Burkina Faso Africa	6–24 months	Community	Not mentioned	Weight-for-height z-score < -2 and ≥-3 of WHO Reference	Weight-for-height z-score ≥ -2
**Vanelli 2014**	Nonrandomized Controlled Trial	Sierra Leone, Africa	6–59 months	Hospital	Not mentioned	Weight-for-height z-score < -2 and > -3 of WHO Reference	Weight-for-height z-score value of -1.0 to less than -2.0

Footnotes

1. Height used as a proxy for age in inclusion criteria: 65 to < 110 cm (used as a proxy for the age of 6 to 59 months)

**Table 2:** Details of intervention.

Study	Supplement Provided	Duration	Calories	Composition of Lipid-based nutrient supplements Used	Milk Based	Control	Cost
Ackatia-Armah 2015 (C)	Ready-to-use supplementary foods (Supplementary Plumpy)	12 weeks	500 kcal/d	• Peanut paste, sugar, • vegetable oil, whey and soy protein isolates, maltodextrin and cocoa flavoring, and a vitamin-mineral complex	No	1. Corn soy blend plus plus 2. Misola 3. Cereal-legume milled flour mix	• Daily cost 1. $0.38 for Ready-to-use supplementary foods 2. $0.22 for corn soy blend plus plus 3. $0.21 for Misola 4.$0.18 for LMF.
Delchevalerie 2015 (C)	Ready-to-use supplementary foods (Supplementary Plumpy)	• until recovery, transfer, • defaulting or death • occurred	1000 kcal/day	• Peanut paste, sugar, • vegetable oil, whey and soy protein isolates, maltodextrin and cocoa flavoring, and a vitamin-mineral complex	No	Corn soy blend Oil premix	Not mentioned
Karakochuk 2012 (C)	Ready-to-use supplementary foods (Supplementary Plumpy)	16 weeks	500 kcal/day	• Peanut paste, sugar, • vegetable oil, whey and soy protein isolates, maltodextrin and cocoa flavoring, and a vitamin-mineral complex	No	Corn soy blend	Not mentioned
LaGrone 2012	• Soy ready-to-use supplementary foods • Soy/Whey ready-to-use supplementary foods (PlumpySup)	12 weeks	500 kcal/day	• Extruded soy flour, peanut paste, sugar, soy • oil, minerals and vitamins (Nutriset), and dicalcium phosphate or calcium carbonate • (Roche) • Peanut paste, sugar, • vegetable oil, whey and soy protein isolates, maltodextrin and cocoa flavoring, and a vitamin-mineral complex	• No •No	Corn soy blend plus plus	• Daily Cost • $0.22 for Soy ready-to-use supplementary foods • $0.38 for Soy/Whey • ready-to-use supplementary foods • $0.16 for corn soy blend plus plus
Matilsky 2009	• Milk Peanut Fortified Spread • Soy Peanut Fortified Spread	8 weeks	75 kcal/kg/day	• 27% peanut paste/26% soy • Flour, Vegetable Oil, Sugar • 26% peanut paste/25% dry skimmed milk, Vegetable Oil, Sugar	• Yes • No	Corn soy blend	• For 1000kj(239kcal) • Milk/peanut FS: US$ 0.16 • Soy/peanut FS:US$ 0.08 • Corn soy blend: US$ 0.04
Medoua 2016	Ready-to-use supplementary foods	8 weeks	40 kcal/kg/day	1. Precooked soy and corn flour 2. Peanut paste 3. Sugar 4. Soy Oil 5. Premix with minerals and vitamins	No	Corn soy blend plus	1·32 €/kg, or 0·080 € for an average daily ration
Nackers 2010	Ready-to-use therapeutic foods [Plumpy’Nut]	16 weeks	1000 kcal/day	Peanuts, non-hydrogenated vegetable fat (palm, rapeseed),sugar, skimmed milk powder, whey powder, maltodextrin (wheat or corn), vitamin and mineral complex, emulsifiers: vegetable lecithin (soy or sunflower), mono and diglycerides, and stabilizers: hydrogenated vegetable fat.	Yes	Corn soy blend pre-mix	Not mentioned
Nikiema 2014 (C)	Ready-to-use supplementary foods (50 g/d) (Fortified Spread)	12 weeks	250 kcal/day	• Peanut butter (26%), vegetable oil (12.5%), • sugar (25%), whole soy flour (33%), shea butter (2.0%), and • multiple micronutrients (1.5%).	No	1. Child Centred Counselling 2. Fortified Corn Soy Blend (corn soy blend plus plus)	Not mentioned
Vanelli 2014	Ready-to-use therapeutic foods (Parma Pap)^1^	12 weeks	1000–1200 kcal/day	Peanuts (25%),sugar (28%), palm oil (15%), milk powder (30%), mineral vitamin mix (1.6% weight)	Yes	United Nations World Feeding Programme Supplementations regimen only	Not mentioned

Footnotes

1. The intervention group received both ready-to-use therapeutic foods AND Food Programme Supplementation

#### Excluded studies

Reasons for the exclusion of individual trials are listed in S5 Table. Of the 21 publications from 19 trials [14, 17, 50–68] the most common reason for exclusion was that study participants did not have moderate acute malnutrition (12/21). In two trials, the intervention did not comprise lipid-based nutrient supplements, three were observational studies, one study used participants with severe acute malnutrition after the stabilization phase, in two studies the outcomes were not of interest, and in one trial both intervention arms received lipid-based nutrient supplements with no control group.

### Risk of bias in included studies

**Fig 2:**
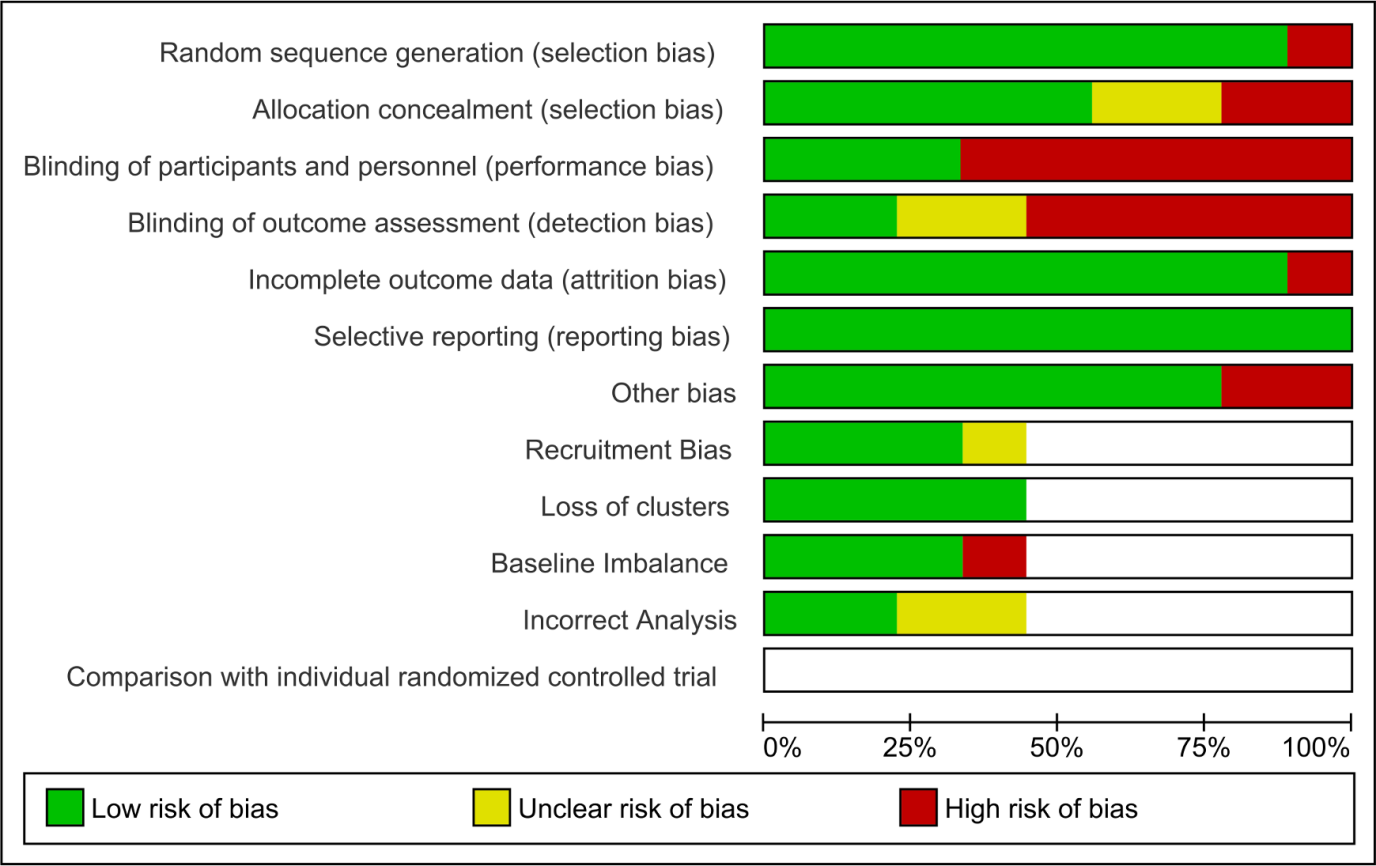
Risk of bias graph. Review authors' judgements about each risk of bias item presented as percentages across all included studies.

**Fig 3:**
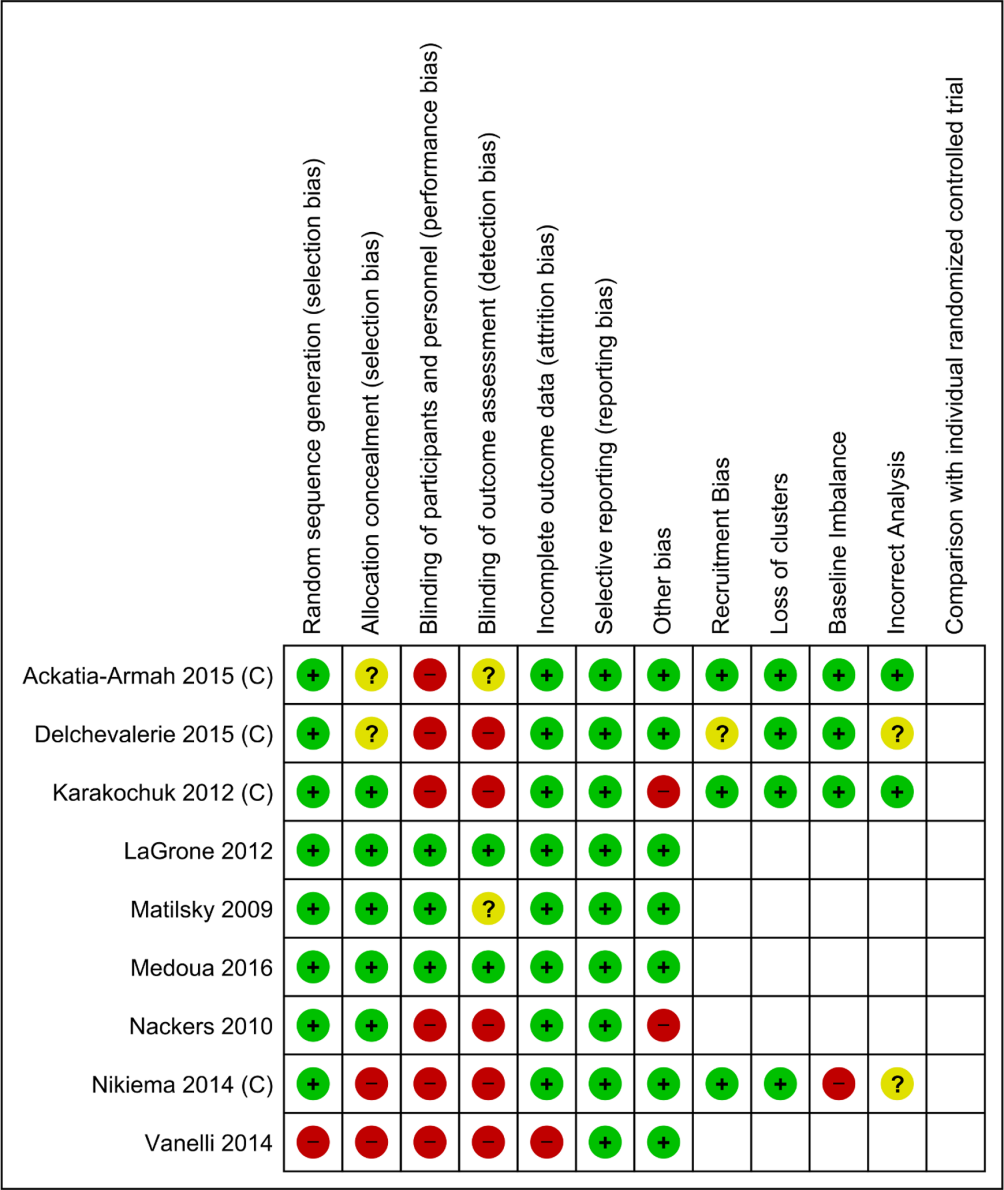
Risk of bias summary. Review authors' judgements about each risk of bias item for each included study.

Figs 2 and 3 summarises the Risk of Bias for the included studies.

#### Allocation (selection bias)

The risk of bias for the eight randomized controlled trials was low for random sequence generation. In one non-randomized trial [45], the risk of bias was high.

Allocation concealment was adequately performed in five of the nine included trials for which the risk of bias was judged to be low. The details of allocation concealment were not clear from the published text for two trials, in which the selection bias was judged to be unclear. For two trials, where allocation concealment was not done [41, 45], the risk of bias was considered to be high.

#### Blinding (performance bias and detection bias)

Lipid-based nutrient supplements are ready to eat supplements, supplied in packets to the participants. The control group, on the other hand, received supplements which required cooking for consumption or received nutritional counselling. Owing to the nature of these interventions, it was not possible for the participants to be blinded. However, some investigators ensured that field personnel and those performing anthropometry or other investigations were blinded to the allocation of study participants. Such trials were considered at low risk for performance bias [22, 39, 40, 48]. For the remaining six trials, the risk of performance bias was judged to be high.

The risk of bias for blinding for outcome assessment was low for two trials [39, 40, 48], unclear for two trials [35–38, 22] and was considered high for the remaining five trials.

#### Incomplete outcome data (attrition bias)

Attrition bias was low for eight out of the nine included trials. In one trial [45], the overall attrition was high and differed significantly in the two comparison groups.

#### Selective reporting (reporting bias)

Selective reporting was not detected in any of the included trials.

#### Other potential sources of bias

Two studies were judged to be at high risk of bias for other reasons. In Karakochuk et al. [47], the quantity of the corn soy blend ration was much higher than ready-to-use supplementary foods, because it was assumed that there would be significant household food sharing of the corn soy blend ration. Due to communication problems, during the course of the study in Nackers et al. [49], children weighing > 8 kg at any time during their treatment were provided with three daily packs of ready-to-use therapeutic foods instead of two as planned in the study protocol. Consequently, these children were excluded from the analysis.

#### Recruitment bias

Of the four cluster randomized controlled trials, recruitment bias was judged to be low in three. In Delchevalerie et al. [46], the risk of bias was considered unclear as the sequence of recruitment was not clear from the published text.

#### Loss of clusters

None of the trials reported any loss of clusters.

#### Baseline imbalance

The risk of bias due to baseline imbalance was considered high in Nikiema et al. [41], where the baseline comparability of the clusters was not reported. However, the lipid-based nutrient supplements group had a slightly better nutritional status and lower morbidity at baseline. This risk of bias for the other three trials was considered to be low.

#### Incorrect analyses

The cluster effect was clearly taken into account in two of the four trials. In the remaining two, author clarifications are awaited.

### Comparison 1: Lipid-based nutrient supplements versus specially formulated micronutrient fortified foods (9 studies, 9270 participants)

#### Proportion of children recovered

**Fig 4:**
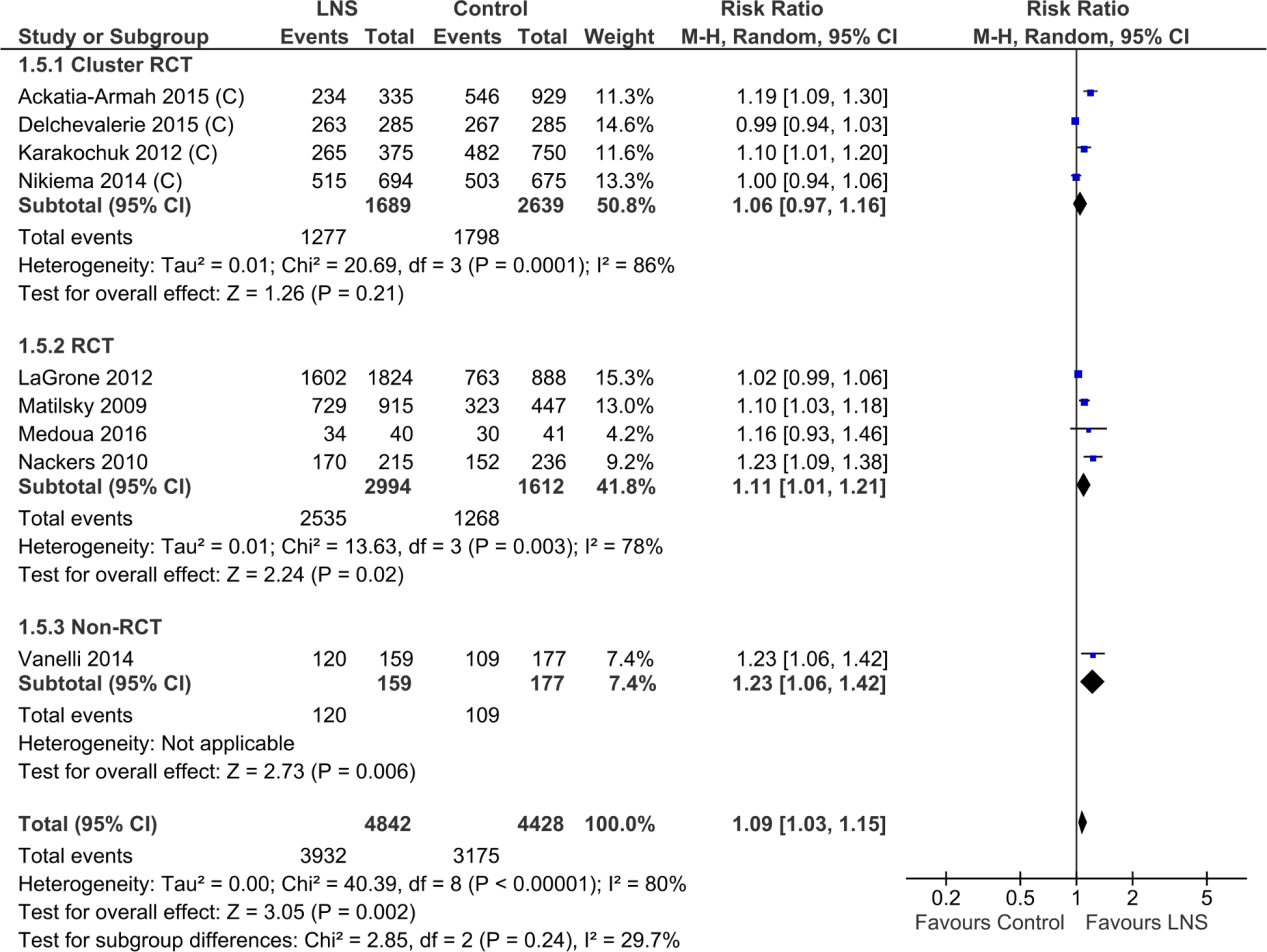
Forest plot Lipid-based nutrient supplement versus Specially formulated micronutrient fortified foods Outcome: 1.5 Recovery from moderate acute malnutrition (ALL).

All nine trials (9270 children) reported this outcome. The meta-analyses of the eight randomized controlled trials showed that the overall recovery rate was 8% higher in the lipid-based nutrient supplement group (RR 1.08; 95% CI 1.02 to 1.14, 8 studies, 8934 participants, low quality evidence). One non-randomized controlled trial also showed higher recovery rate in the lipid based nutrient supplement group (Table 3, S8 Table Analysis 1.5; Fig 4). There was significant heterogeneity between trials (I^2^ = 80%, p<0.00001). On stratified analyses based on study designs, effect size was a little lower for cluster randomized controlled trials (4 trials, 4328 participants, RR 1.06, 95% CI 0.97, 1.16) as compared to randomized controlled trials (RR 1.11, 95% CI 1.01 to 1,21, 4 studies, 4606 participants) and non-randomized controlled trials (RR 1.23, 95% CI 1.06 to 1.42, 1 study, 336 participants, very low quality of evidence). The test for subgroup difference showed low heterogeneity (I^2^ = 29.7%, p = 0.24).

**Table 3:** Summary of findings: Lipid-based nutritient supplements compared to Specially formulated micronutrient fortified foods for treatment of moderate acute malnutrition in children (6 months to 59 months) with Moderate Acute Malnutrition.

Lipid-based nutrient supplements **compared to Specially formulated micronutrient fortified foods for treatment of** moderate acute malnutrition **in children (6 months to 59 months)**						
**Patient or population:** Children (6 to 59 months) with moderate acute malnutrition in **Settings:** **Intervention:** Lipid-based nutrient supplements lipid-based nutrient supplements **Comparison:** Specially Formulated Fortified Foods						
**Outcomes**	Illustrative comparative risks* **(95% CI)**		**Relative effect** **(95% CI)**	**No of Participants** **(studies)**	**Quality of the evidence** (GRADE)**	**Comments**
	**Assumed risk**	**Corresponding risk**				
	**Specially Formulated Fortified Foods**	**Lipid-based nutrient supplements**				
**Recovery from** moderate acute malnutrition	**Study population**		**RR 1.08** (1.02 to 1.14)	8934 (8 studies)	⊕⊕⊝⊝ **low**^1^^,^^2^	
	**721 per 1000**	**779 per 1000** (736 to 822)				
	**Moderate**					
	**723 per 1000**	**781 per 1000** (737 to 824)				
**No recovery**	**Study population**		**RR 0.70** (0.58 to 0.85)	8364 (7 studies)	⊕⊕⊝⊝ **low**^2^^,^^3^	
	**184 per 1000**	**129 per 1000** (107 to 156)				
	**Moderate**					
	**148 per 1000**	**104 per 1000** (86 to 126)				
**Duration to Recovery**		The mean duration to recovery in the intervention groups was **4.77 days lower** (12.54 lower to 3 higher)		2020 (3 studies)	⊕⊝⊝⊝ **very low**^2^^,^^4^^,^^5^^,^^6^	
**Deterioration to** severe acute malnutrition	**Study population**		**RR 0.87** (0.73 to 1.03)	6788 (5 studies)	⊕⊕⊝⊝ **low**^2^^,^^7^	
	**69 per 1000**	**60 per 1000** (50 to 71)				
	**Moderate**					
	**70 per 1000**	**61 per 1000** (51 to 72)				
**Tranferred to Inpatient**	**Study population**		**RR 0.57** (0.24 to 1.34)	4939 (5 studies)	⊕⊕⊝⊝ **low**^2^^,^^8^	
	**31 per 1000**	**18 per 1000** (8 to 42)				
	**Moderate**					
	**11 per 1000**	**6 per 1000** (3 to 15)				
**Mortality**	**Study population**		**RR 0.91** (0.54 to 1.52)	8934 (8 studies)	⊕⊝⊝⊝ **very low**^1^^,^^2^^,^^9^	
	**6 per 1000**	**6 per 1000** (3 to 10)				
	**Moderate**					
	**4 per 1000**	**4 per 1000** (2 to 6)				
**Post Discharge Mortality**	**Study population**		**RR 0.62** (0.28 to 1.37)	2859 (3 studies)	⊕⊝⊝⊝ **very low**^2^^,^^5^^,^^9^^,^^10^	
	**42 per 1000**	**26 per 1000** (12 to 58)				
	**Moderate**					
	**49 per 1000**	**30 per 1000** (14 to 67)				
**Weight Gain (g/kg/d)**		The mean weight gain (g/kg/d) in the intervention groups was **0.62 higher** (0.18 to 1.06 higher)		5054 (5 studies)	⊕⊕⊝⊝ **low**^2^^,^^11^	
**Weight Gain Total**		The mean weight gain total in the intervention groups was **0.23 higher** (0.14 to 0.32 higher)		1264 (1 study)	⊕⊕⊝⊝ **low**^2^^,^^12^	
Weight-for-height z-score **End**		The mean weight-for-height z-score end in the intervention groups was **0.1 higher** (0.05 to 0.14 higher)		5443 (3 studies)	⊕⊕⊝⊝ **low**^2^^,^^13^	

Footnotes

^1^ Downgraded by 1 for Risk of Bias across the studies.Of the 9 included trials one study had a high risk of bias for random sequence generation, two for allocation concealment, 6 for blinding of participants and personnel, 5 for blinding of outcome assessment, one for attrition, 2 trials had other sources of bias and one had baseline imbalance between clusters.

^2^ Downgraded by 1 for Indirectness as all studies were conducted in Africa. Extrapolation to other areas where moderate acute malnutrition is prevalent like Asia and Latin America, with different co-morbidities and reasons for malnutrition not possible.

^3^ Downgraded by 1 for serious risk of bias. One trial had high risk of bias for random sequence generation, two for allocation concealment, 5 for blinding of participants, 4 for blinding of outcome assessment, one for attrition bias, two for other bias and one for baseline balance between clusters.

^4^ Downgraded by 1 for serious risk of bias. One trial had high risk of bias for allocation concealment, both for blinding of participants and outcome assessment, and one for baseline balance between clusters.

^5^ Downgraded by 1 for serious risk for inconsistency. The studies have widely differing estimates of the treatment effect.

^6^ Downgraded by 1 for imprecision. There are few studies, with few study participants and estimates have wide confidence intervals around the estimate of the effect

^7^ Downgraded by 1 for serious risk of bias. One (of 6 trials) had high risk of bias for random sequence generation, two for allocation concealment, three for blinding of participants and personnel, two for blinding of outcome assessment, one for attrition and one for baseline imbalance of clusters.

^8^ Downgraded by 1 for serious risk of bias. Of the four included trials, two were at high risk of bias for blinding of participants and personnel, blinding of outcome assessment and other bias.

^9^ The included studies had few events and thus wide confidence intervals around the estimate of the effect.

^10^ Downgraded by 1 for serious risk of bias. Of the three included trials two had high risk of bias for blinding of participants and personnel and blinding of outcome assessment and one for other bias.

^11^ Downgraded by 1 for serious risk of bias. Three studies were at high risk of bias for blinding of participants and personnel and outcome assessment, and one each for allocation concealment, other bias and baseline balance between clusters.

^12^ Outcome assessed included just one trial, results of which cannot be extrapolated to other settings

^13^ Downgraded by 1 for risk of bias. Of the three included trials one had high risk of bias for blinding, allocation concealment and baseline balance between clusters.

*The basis for the **assumed risk** (e.g. the median control group risk across studies) is provided in footnotes. The **corresponding risk** (and its 95% confidence interval) is based on the assumed risk in the comparison group and the **relative effect** of the intervention (and its 95% CI).

**GRADE Working Group grades of evidence

**High quality:** Further research is very unlikely to change our confidence in the estimate of effect.

**Moderate quality:** Further research is likely to have an important impact on our confidence in the estimate of effect and may change the estimate.

**Low quality:** Further research is very likely to have an important impact on our confidence in the estimate of effect and is likely to change the estimate.

**Very low quality:** We are very uncertain about the estimate.

**Fig 5:**
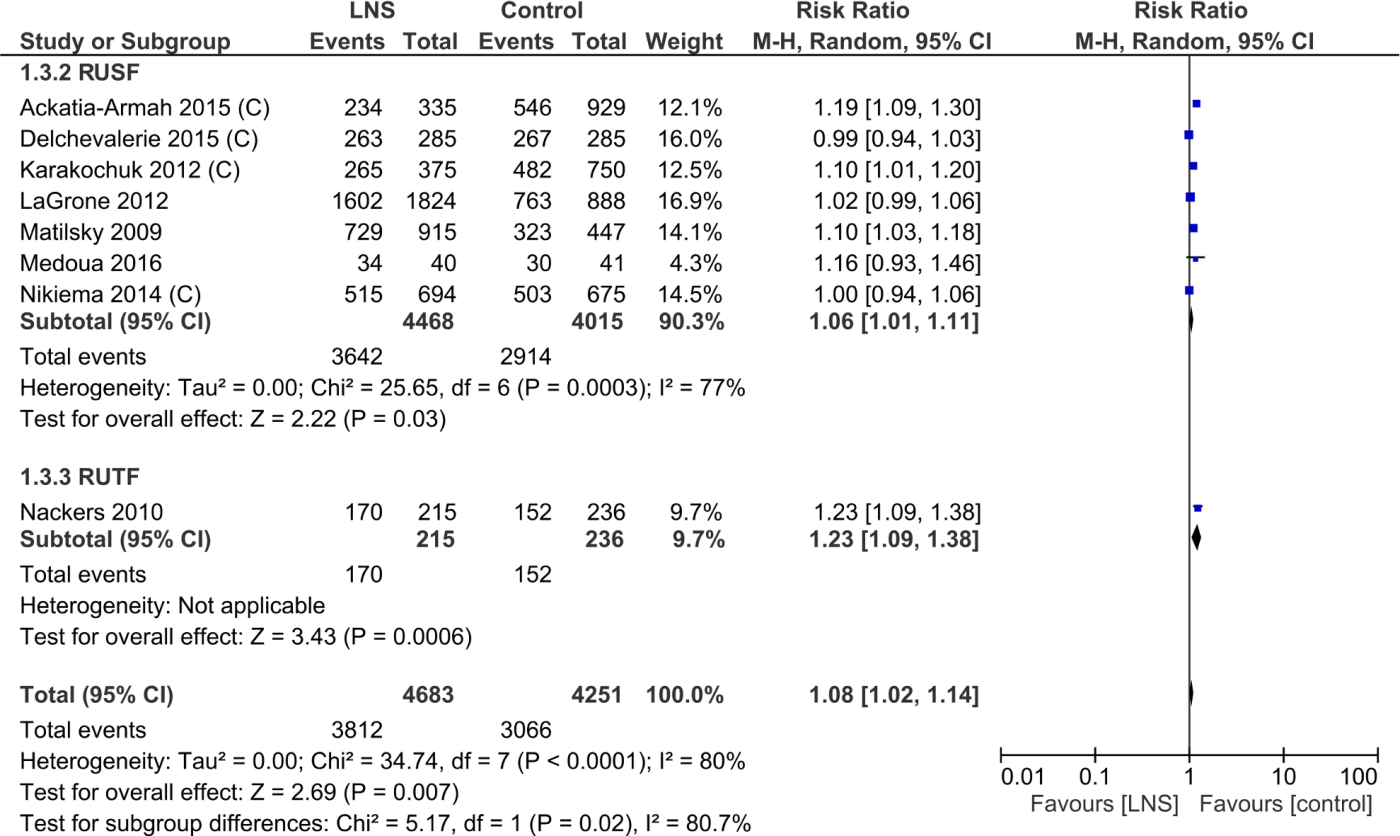
Forest plot: Lipid-based nutrient supplement versus Specially formulated micronutrient fortified foods, outcome: 1.3 Recovery from moderate acute malnutrition (SUBGROUP: by type of supplement).

**Fig 6:**
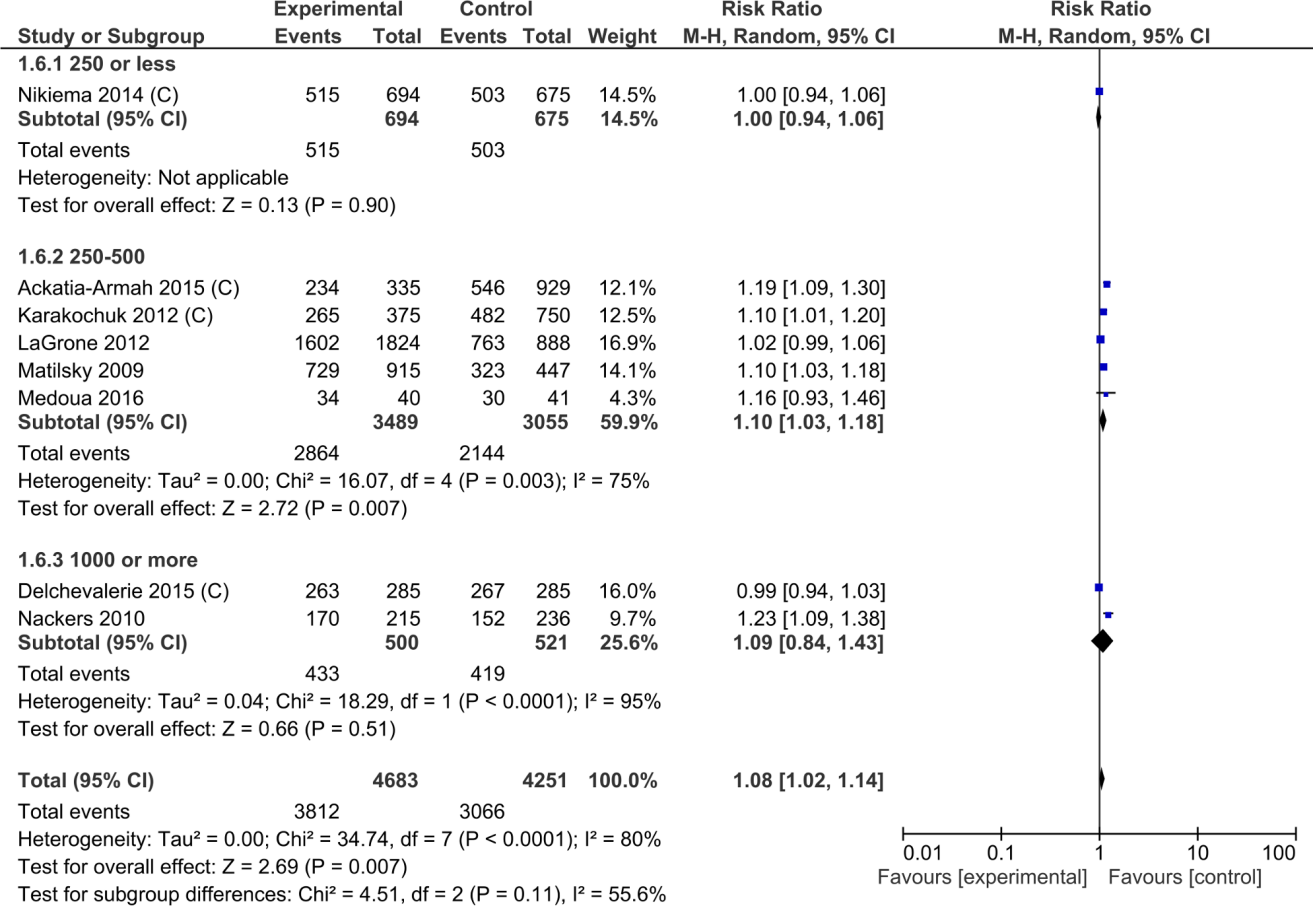
Forest plot: Lipid-based nutrient supplement versus Specially formulated micronutrient fortified foods, outcome: 1.6 Recovery from moderate acute malnutrition (SUBGROUP: Calories provided).

In subset analyses to explore heterogeneity, the type of macronutrient provided was associated with the recovery rate; ready-to-use therapeutic foods had a higher recovery rate than ready-to-use supplementary foods (RR 1.23, 95% CI 1.09, 1.38; one study for ready-to-use therapeutic foods; RR 1.06, 95% CI 1.01, 1.11 for ready-to-use supplementary foods; P = 0.02 for subgroup differences) (S8 Table, Analysis 1.3; Fig 5). Lipid-based nutrient supplements containing milk also had a higher recovery rate (RR 1.15, 95% CI 1.03, 1.29) than those without milk (RR 1.06, 95% CI 1.01, 1.11; overlapping CIs) (S8 Table, Analysis 1.7). Age group of the participants (Table 3, Analysis 1.2), duration of supplementation (S8 Table, Analysis 1.4) calories provided per day (S8 Table, Analysis 1.6; Fig 6) did not explain the heterogeneity of effect.

LaGrone et al. [39, 40] performed binary logistic regression analysis to evaluate the effect of various factors on recovery in the two treatment groups. Children with HIV recovered less frequently [53 of 84 (63%) compared with 2310 of 2628 (88%); P <0.0001]. HIV positive children also had a higher risk of developing severe wasting [19 of 31 (61%) compared with 126 of 318 (40%), P < 0.03] and development of kwashiorkor was less frequent in HIV-positive children [4 of 31 (13%) compared with 116 of 318 (36%); P <0.01]. Recovery rate was higher in children receiving antiretroviral therapy (19 of 24 (79.2%)versus 31 of 54 (57.4%); RR: 1.38; 95% CI: 1.01, 1.88). These results were not affected by the type of supplementary food the child received.

Overall time taken to recovery was a little lower in children receiving lipid-based nutrient supplements. Three trials provided the mean duration (days) taken to recover from moderate acute malnutrition. Meta-analyses of these trials showed slightly shorter duration to recovery, but with wide confidence intervals (n = 2020; MD -4.77, 95% CI -12.54 to 3.0) (Table 3, S8 Table, Analysis 1.9). LaGrone [39, 40] does not provide detailed data; however, they mention that the mean duration of treatment required to achieve recovery was 23 days in the total study cohort. Of these, the children who received corn soy blend plus plus took an average of 2 days longer to recover (ANOVA, P < 0.003). Also, more than half of the children recovered in the first two weeks of therapy itself. Nackers et al. [49] mention the length of stay (to recovery) as median and range. This was significantly lower for the lipid-based nutrient supplements group (median 4 weeks; range 2 to 16 weeks), when compared with the corn soy blend cohort (median 6 weeks; range 2 to 16 weeks). Matilsky et al. [22] also mention the length of stay as median and 25th and 75th centile. This was lower in both the lipid-based nutrient supplements groups (median 14 days; IQR 14, 42), when compared to corn soy blend (median 28 days; Interquartile Range 14, 56). The mean period of food supplementation for children receiving corn soy blend was 4.0 weeks and the mean period for children receiving soy/peanut Fortified Spread or milk/peanut Fortified Spread was 3.3 weeks. Vanelli [45] report that the children in the intervention group (who received United Nations World Food Programme Supplementation portions and lipid-based nutrient supplements) recovered faster (mean duration 5.54 weeks) than those treated with Food Programme Supplementation regimen only (mean 8.16 weeks).

#### No recovery

**Fig 7:**
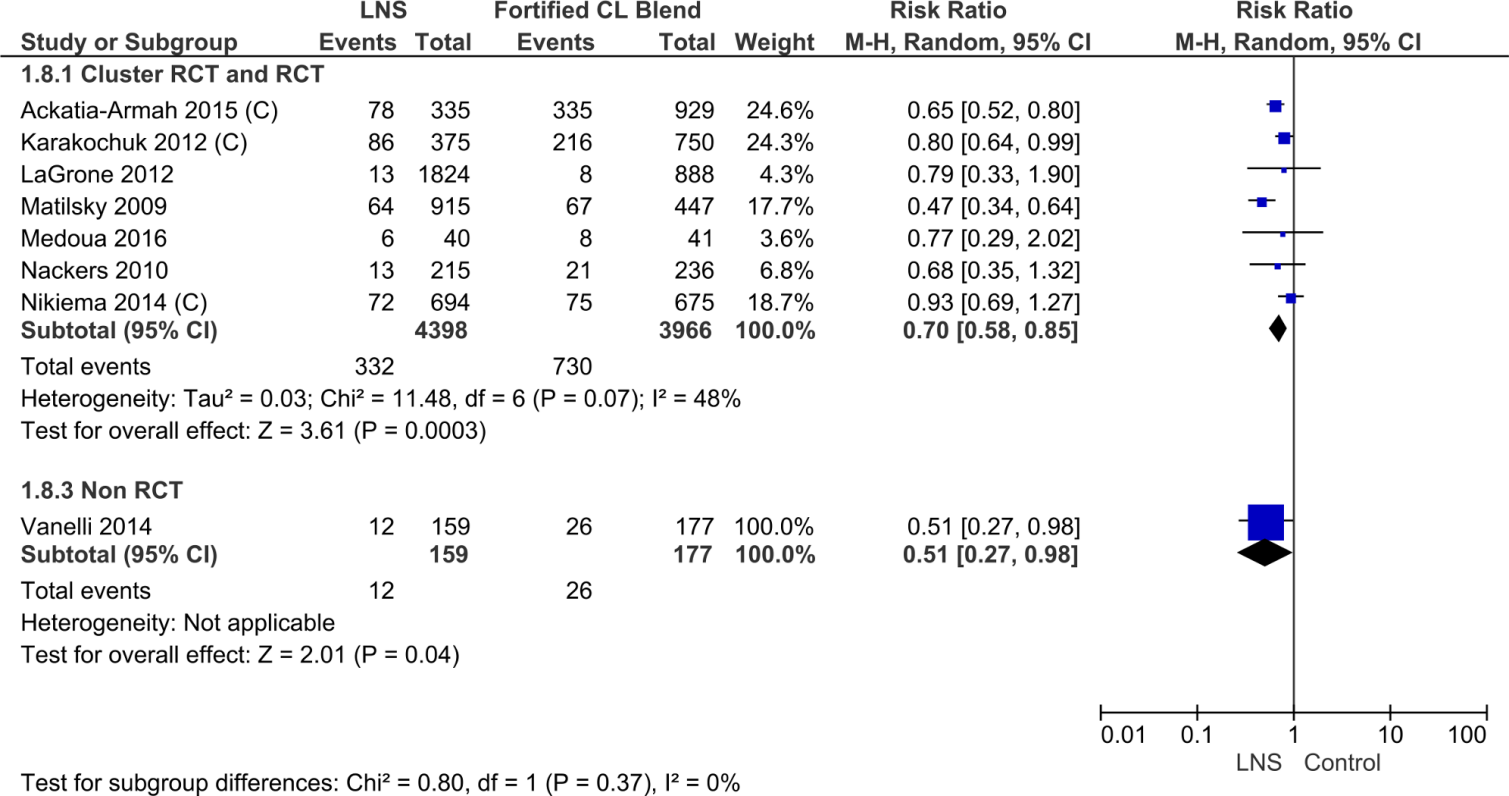
Forest plot: Lipid-based nutrient supplement versus specially formulated micronutrient fortified foods, outcome: 1.2 No recovery.

The number of children who did not recover was lower in the lipid-based nutrient supplements group (RR 0.70, 95% CI 0.58 to 0.85, 7 randomized controlled trials, 8364 participants) (Table 3, S8 Table, Analysis 1.8; Fig 7). The effect size was almost the same for the various study designs; for cluster randomized controlled trials (3 trials, 3758 study participants, RR 0.77, 95% CI 0.63, 0.94), for randomized controlled trials (4 trials, 4606 study participants, RR 0.54, 95% CI 0.41, 0.70) and for 1 non-randomized controlled trial (1 trial, 336 study participants, RR 0.51, 95% CI 0.27, 0.98) (45). There was little heterogeneity of effect among trials (I^2^ = 48%, p = 0.07) or among subgroups (I^2^ = 0%, p = 0.37).

#### Mortality

**Fig 8:**
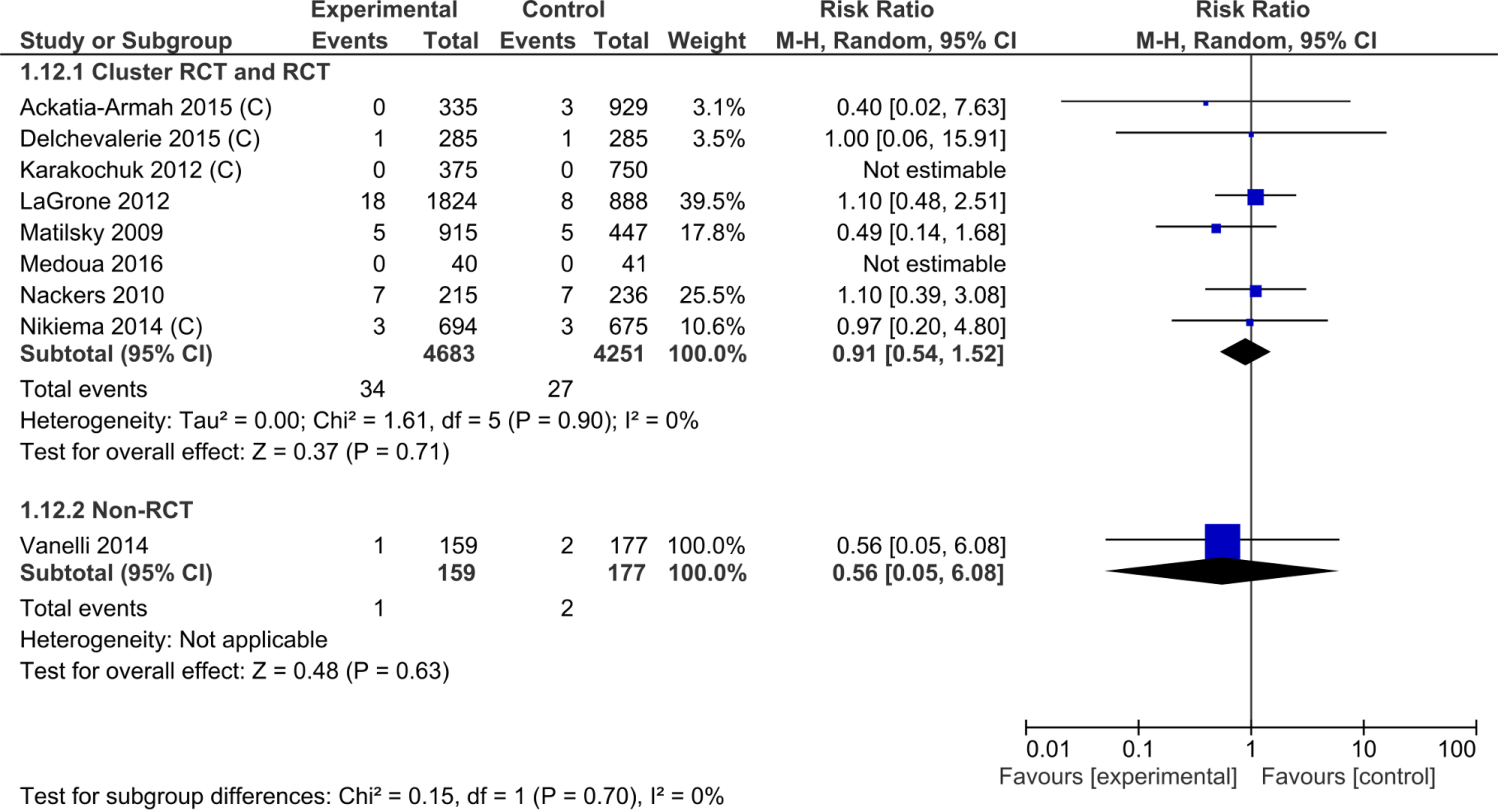
Forest plot: Lipid-based nutrient supplement versus specially formulated micronutrient fortified foods, outcome: 1.6 Mortality.

All nine trials (9270 children) reported this outcome. In the eight randomized controlled trials, the overall mortality was not affected by the type of supplement provided (RR = 0.91; 95% CI 0.54, 1.42) (Table 3, S8 Table, Analysis 1.12; Fig 8). There was no heterogeneity of effect between trials (I^2^ = 0%, p = 0.90). The effect size was similar for cluster randomized controlled trials (4 trials, 4328 study participants, RR 0.83, 95% CI 0.24, 2.91), randomized controlled trials (4 trials, 4606 study participants, RR 0.92, 95% CI 0.52, 1.63) and non-randomized controlled trial (1 trial, 336 study participants RR 0.56, 95% CI 0.05, 6.08). The tests for subgroup difference showed no heterogeneity (I^2^ = 0%, p = 0.70).

**Fig 9:**
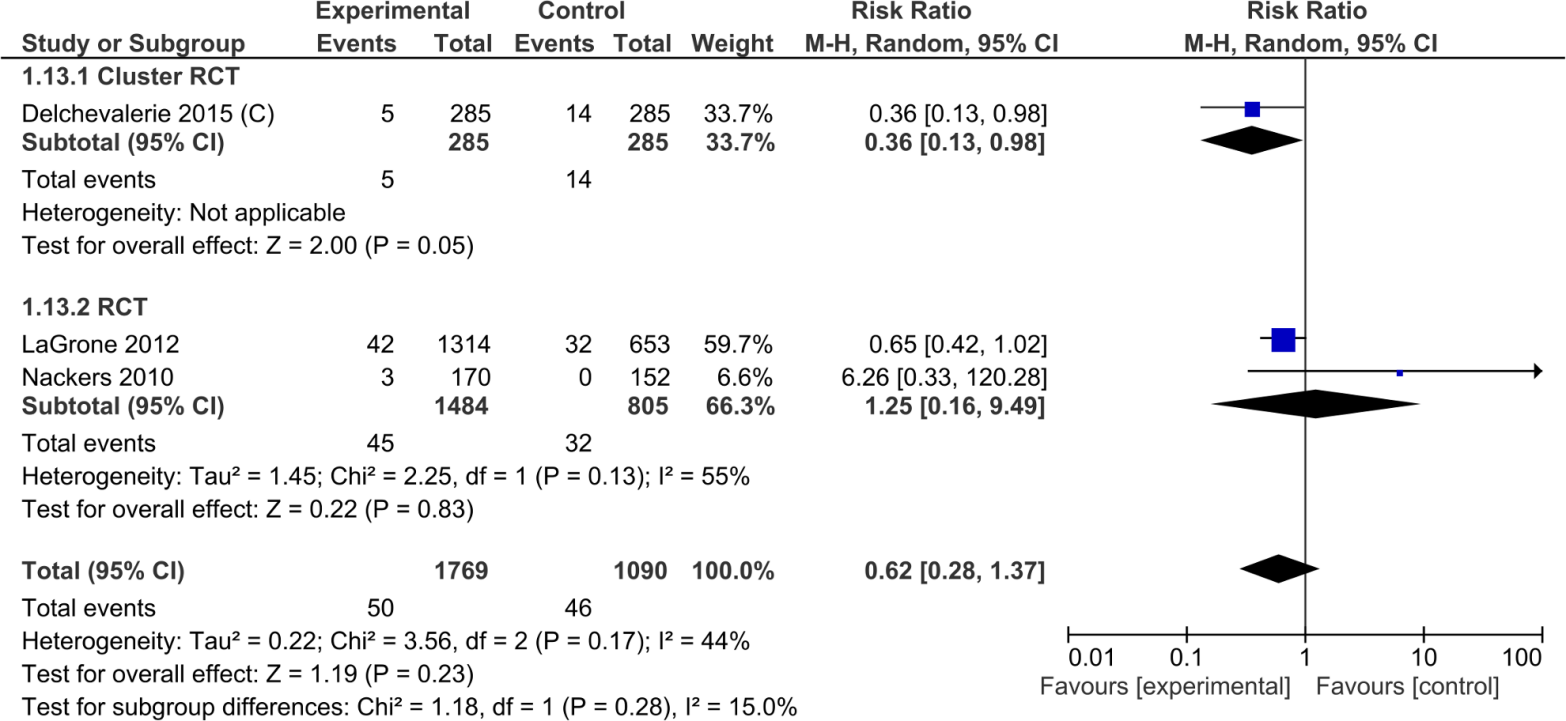
Forest plot: Lipid-based nutrient supplement versus specially formulated micronutrient fortified foods, outcome: 1.7 Post Discharge Mortality.

Long term mortality after supplementation was stopped (6 months to 1 year later) was studied by 3 trials (2859 study participants) (RR 0.62, 95% CI 0.28, 1.37). The results were variable across different study designs. The 1 cluster randomized controlled trial (570 study participants) showed reduction in long term mortality in the lipid-based nutrient supplement group (RR 0.36, 95% CI 0.13, 0.98) while the 2 randomized controlled trials did not show any difference (RR 1.25, 95% CI 0.16, 9.49). There was significant heterogeneity of effect among the subgroups (Table 3, S8 Table, Analysis 1.13; Fig 9).

#### Deterioration to severe acute malnutrition

**Fig 10:**
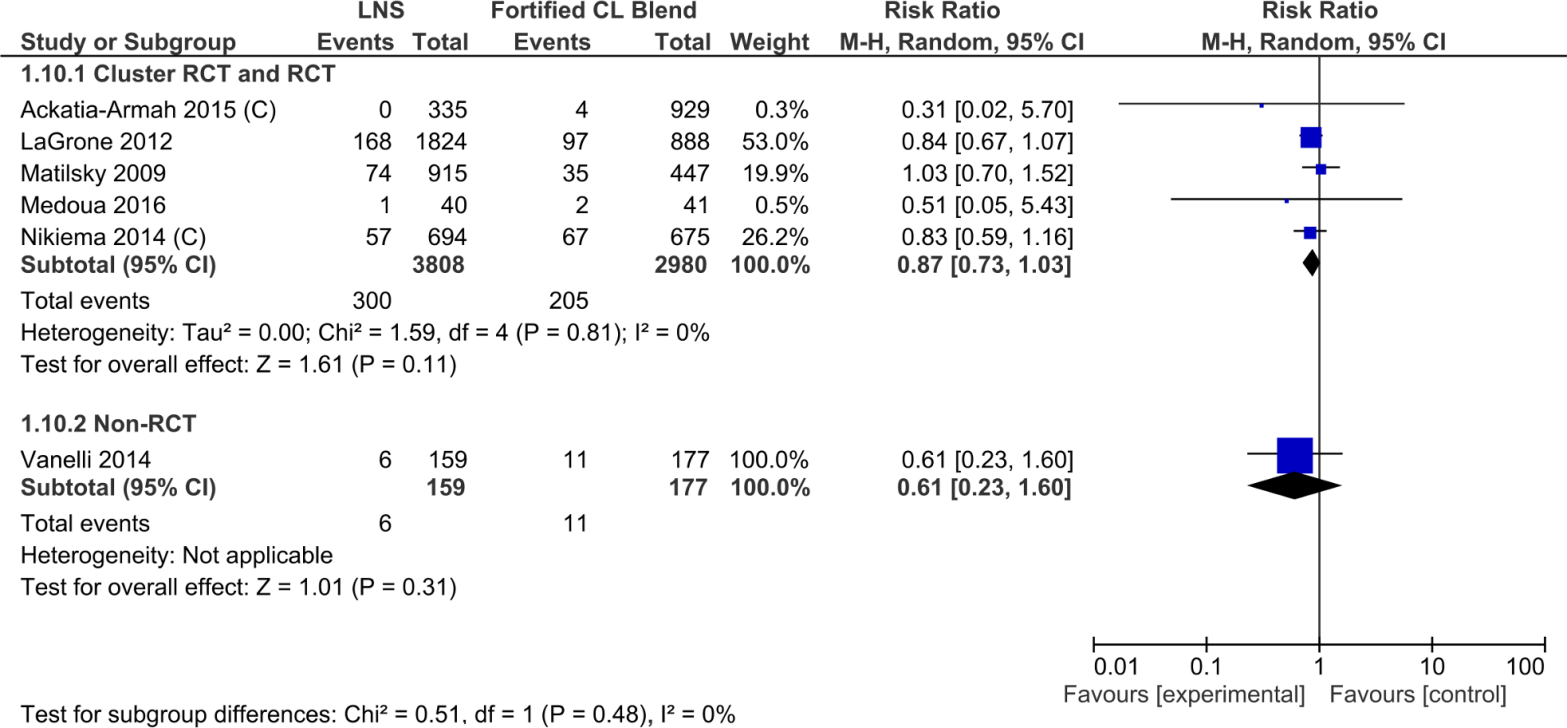
Forest plot: Lipid-based nutrient supplement versus Specially formulated micronutrient fortified foods, outcome: 1.10 Deterioration to severe acute malnutrition.

Data from 6 trials (7124 children) showed that children with moderate acute malnutrition receiving lipid-based nutrient supplements had a lower risk of developing severe acute malnutrition when compared to those receiving Specially Formulated Foods. The meta-analyses of the five randomized controlled trials showed lower risk of developing severe acute malnutrition with lipid based nutrient supplement (RR 0.87, 95% CI 0.73, 1.03). The effect size was almost similar for the two cluster randomized controlled trials (2633 study participants; RR 0.82, 95% CI 0.58, 1.14) and the three randomized controlled trials (4155 study participants; RR 0.89, 95% CI 0.73, 1.09). The effect size was larger and more imprecise for the non-randomized trial (45). There was no significant heterogeneity of effect among the included trials (Table 3, S8 Table, Analysis 1.10; Fig 10)

#### Transferred to inpatient facility

**Fig 11:**
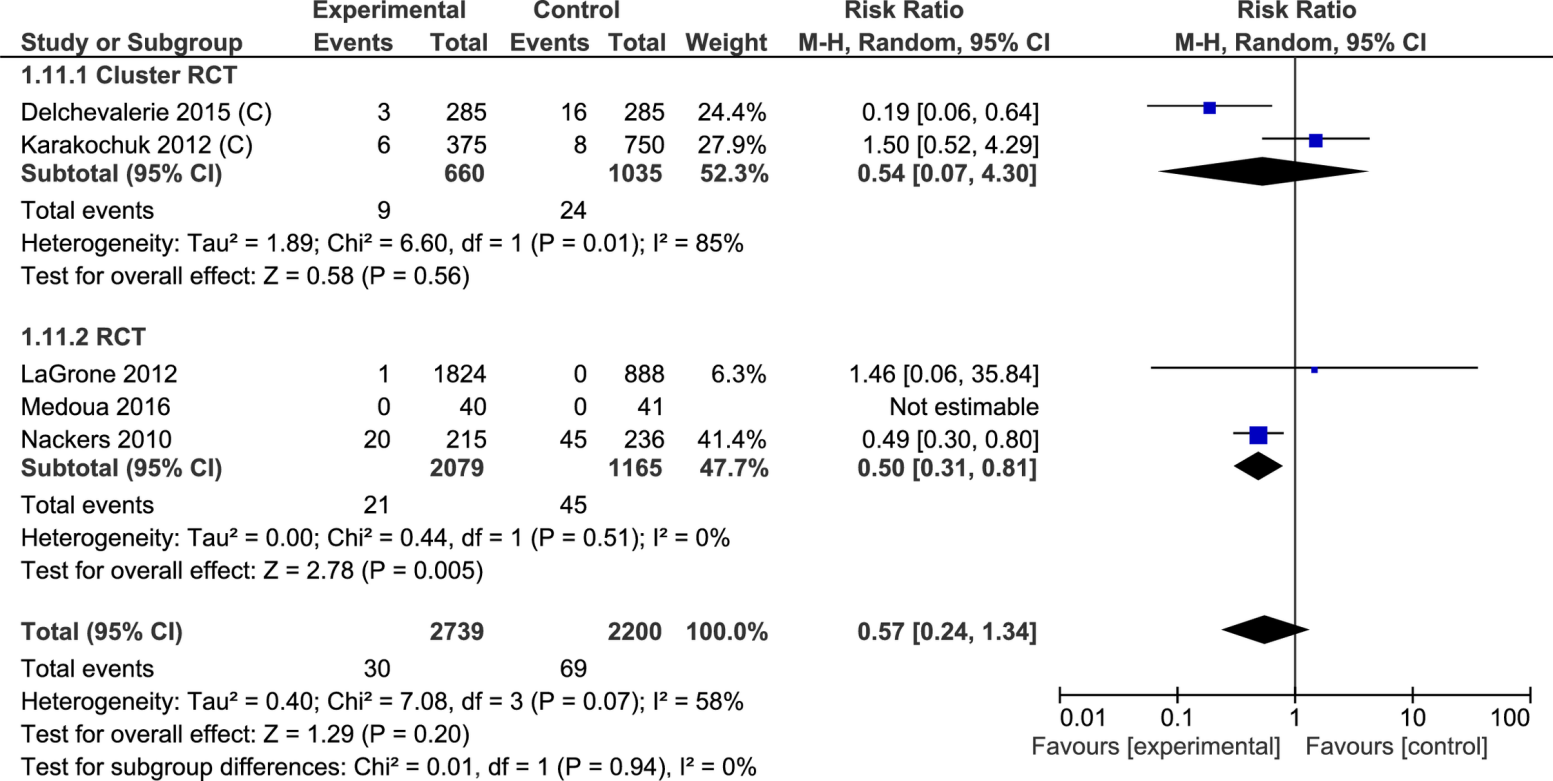
Forest plot: Lipid-based nutrient supplement versus Specially formulated micronutrient fortified foods, outcome: 1.11 transferred to Inpatient.

The patients were transferred to inpatient facilities either because of severe acute malnutrition or development of medical complications. Data for this outcome was reported in 5 trials (2 cluster randomized controlled trials and 3 randomized controlled trials) (Table 3, S8 Table, Analysis 1.11; Fig 11). Children receiving lipid-based nutrient supplements had a lower chance of transfer to in-patient treatment (4939 study participants; RR 0.57, 95% CI 0.24, 1.34). The effect estimate was imprecise with wider confidence intervals for the 2 cluster randomized controlled trials (1695 study participants; RR 0.54, 95% CI 0.07, 4.30) but more precise in the 3 randomized controlled trial (3244 study participants; RR 0.50, 95% CI 0.31, 0.81). There was no significant heterogeneity of effect among the included trials (I^2^ = 58%, p = 0.07) or across the subgroup (I^2^ = 0%, p = 0.94).

#### Relapse after discharge

**Fig 12:**
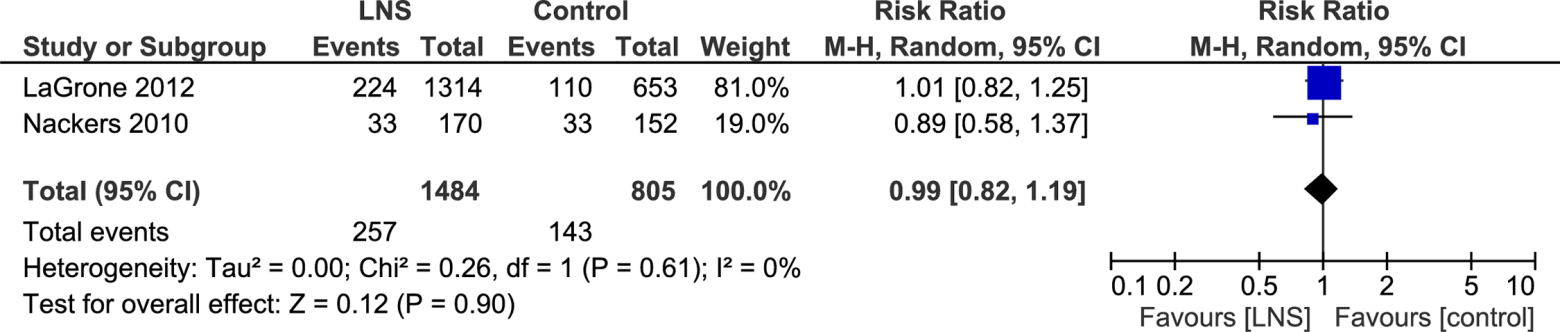
Forest plot: Lipid-based nutrient supplement versus Specially formulated micronutrient fortified foods, outcome: 1.23 Relapse after discharge.

In two randomized controlled trials the risk of relapse was almost 20% in both the groups (n = 2289, RR = 0.99, 95% CI 0.82, 1.19) (Table 4, S8 Table, Analysis 1.23) (Fig 12).

**Table 4:** Summary of findings: Lipid-based nutrient supplements compared to Specially formulated micronutrient fortified foods for treatment of moderate acute malnutrition in children (6 months to 59 months).

Lipid-based nutrient supplements **compared to Specially formulated micronutrient fortified foods for treatment of** moderate acute malnutrition **in children (6 months to 59 months)**						
**Patient or population:** Children (6 to 59 months) with Moderate Acute Malnutrition in **Settings:** **Intervention:** Lipid-based nutrient supplements **Comparison:** Specially Formulated Fortified Foods						
**Outcomes**	Illustrative comparative risks* **(95% CI)**		**Relative effect** **(95% CI)**	**No of Participants** **(studies)**	**Quality of the evidence** (GRADE)**	**Comments**
	**Assumed risk**	**Corresponding risk**				
	**Specially Formulated Fortified Foods**	**Lipid-based nutrient supplements**				
Weight-for-length z-score **Gain**		The mean weight-for-length z-score gain in the intervention groups was **0.28 higher** (0.15 to 0.41 higher)		1264 (1 study)	⊕⊕⊝⊝ **low**^1^^,^^2^	
**Length Gain**		The mean length gain in the intervention groups was **0.16 higher** (0.02 lower to 0.34 higher)		1264 (1 study)	⊕⊝⊝⊝ **very low**^1^^,^^2^^,^^3^	
**Length Gain (mm/d)**		The mean length gain (mm/d) in the intervention groups was **0 higher** (0.03 lower to 0.02 higher)		4081 (2 studies)	⊕⊕⊝⊝ **low**^1^^,^^4^	
Height-for-age z-score **End**		The mean height-for-age z-score end in the intervention groups was **0.16 higher** (0.03 lower to 0.34 higher)		2731 (2 studies)	⊕⊕⊝⊝ **low**^1^^,^^4^	
Mid-upper arm circumference **Gain**		The mean mid-upper arm circumference gain in the intervention groups was **0.29 higher** (0.17 to 0.41 higher)		1264 (1 study)	⊕⊕⊝⊝ **low**^1^^,^^2^	
Mid-upper arm circumference **Gain (mm/day)**		The mean mid-upper arm circumference gain (mm/day) in the intervention groups was **0.04 higher** (0.02 to 0.06 higher)		4474 (4 studies)	⊕⊕⊝⊝ **low**^1^^,^^5^	
**Relapse after discharge**	**Study population**		**RR 0.99** (0.82 to 1.19)	2289 (2 studies)	⊕⊕⊝⊝ **low**^1^^,^^6^	
	**178 per 1000**	**176 per 1000** (146 to 211)				
	**Moderate**					
	**193 per 1000**	**191 per 1000** (158 to 230)				
**Default Rate**	**Study population**		**RR 1.32** (0.73 to 2.4)	7570 (7 studies)	⊕⊕⊝⊝ **low**^1^^,^^7^	
	**22 per 1000**	**29 per 1000** (16 to 52)				
	**Moderate**					
	**16 per 1000**	**21 per 1000** (12 to 38)				
**Hemoglobin (Final)**		The mean hemoglobin (final) in the intervention groups was **0.25 higher** (0.06 to 0.44 higher)		1154 (1 study)	⊕⊕⊝⊝ **low**^1^^,^^2^	
**Change in Hemoglobin**		The mean change in hemoglobin in the intervention groups was **0.36 higher** (0.34 lower to 1.05 higher)		1357 (2 studies)	⊕⊝⊝⊝ **very low**^**8**^^**,**^^**9**^^**,**^ ^1^	

Footnotes

^1^ Downgraded by 1 for indirectness as all studies were conducted in Africa. Extrapolation to other areas where moderate acute malnutrition is prevalent like Asia and Latin America, with different co-morbidities and reasons for malnutrition not possible.

^2^ Outcome assessed included just one trial, results of which cannot be extrapolated to other settings

^3^ Downgraded by 1 for imprecision. There are few studies, with few study participants and estimates have wide confidence intervals around the estimate of the effect

^4^ Downgraded by 1 for risk of bias. Of the two included trials one had high risk of bias for blinding, allocation concealment and baseline balance between clusters.

^5^ Downgraded by 1 for risk of bias. Of the three included trials one was at high risk of bias for allocation concealment, two for blinding of participants and personnel, two for blinding of outcome assessment, one for other bias and one for baseline balance between clusters.

^6^ Downgraded by 1 for seious risk of bias. Of the two included trials one trial was at high risk of bias for blinding of participants and personnel, blinding of outcome assessment, and other bias.

^7^ Downgraded by 1 for serious risk of bias. Of the six included trials one was at high risk of bias for allocation concealment, 4 for blinding of participants and personnel, 4 for blinding of outcome assessment, two for other bias and one for baseline balance between clusters.

^8^ Downgraded by 1 for serious risk of bias. Of the two included trials both were at risk of bias for blinding of participants and personnel, one for blinding of outcome assessment and one for other bias.

^9^ No explanation was provided

*The basis for the **assumed risk** (e.g. the median control group risk across studies) is provided in footnotes. The **corresponding risk** (and its 95% confidence interval) is based on the assumed risk in the comparison group and the **relative effect** of the intervention (and its 95% CI).

**GRADE Working Group grades of evidence

**High quality:** Further research is very unlikely to change our confidence in the estimate of effect.

**Moderate quality:** Further research is likely to have an important impact on our confidence in the estimate of effect and may change the estimate.

**Low quality:** Further research is very likely to have an important impact on our confidence in the estimate of effect and is likely to change the estimate.

**Very low quality:** We are very uncertain about the estimate.

#### Weight gain

**Fig 13:**
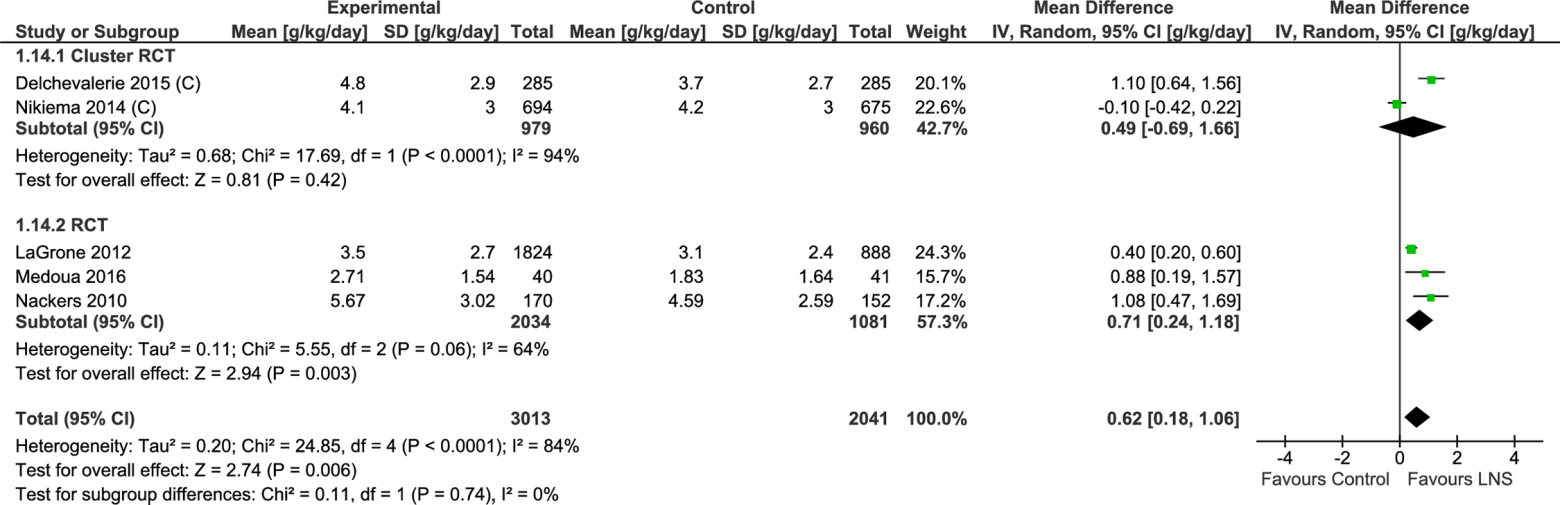
Forest plot: Lipid-based nutrient supplement versus Specially formulated micronutrient fortified foods, outcome: 1.14 Weight Gain (g/kg/d) [g/kg/day].

Seven trials reported data on weight gain in the study participants. Of these, 6 trials reported this as g/kg/day. In quantitative synthesis from 5 trials (Table 3, S8 Table, Analysis 1.14; Fig 13), weight gain was higher with lipid-based nutrient supplements (5054 study participants; MD = 0.62 gm/kg/day; 95% CI = 0.18, 1.06). The effect size was similar but with wider confidence intervals for the cluster randomized controlled trials (2 trials, 1939 study participants; MD = 0.49 gm/kg/day; 95% CI = -0.69, 1.66) as compared to randomized controlled trials (3 trials; 3115 study participants; MD = 0.71 gm/kg/day; 95% CI = 0.24, 1.18). Matilsky et al. [22] compared the weight gain (g/kg/day) at each two week interval in the lipid-based nutrient supplements and control group and depicted the data as a figure (not included in the meta-analysis). Rates of weight gain were greater (P< 0.05) with lipid-based nutrient supplements compared to corn soy blend, except in the 6 to 8 week interval.

**Fig 14:**
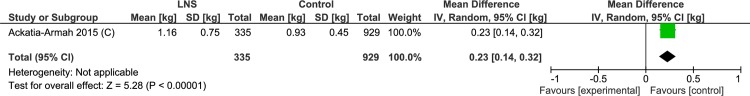
Forest plot: Lipid-based nutrient supplement versus Specially formulated micronutrient fortified foods, outcome: 1.15 Weight Gain Total [kg].

One cluster randomized controlled trial [35–38] provided information on the absolute weight gain after the supplementation period and showed a differential weight gain of 0.23 kg (95% CI 0.14, 0.32) with lipid-based nutrient (Tables 3, S8, Analysis 1.15; Fig 14).

#### Weight-for-height z-score

**Fig 15:**
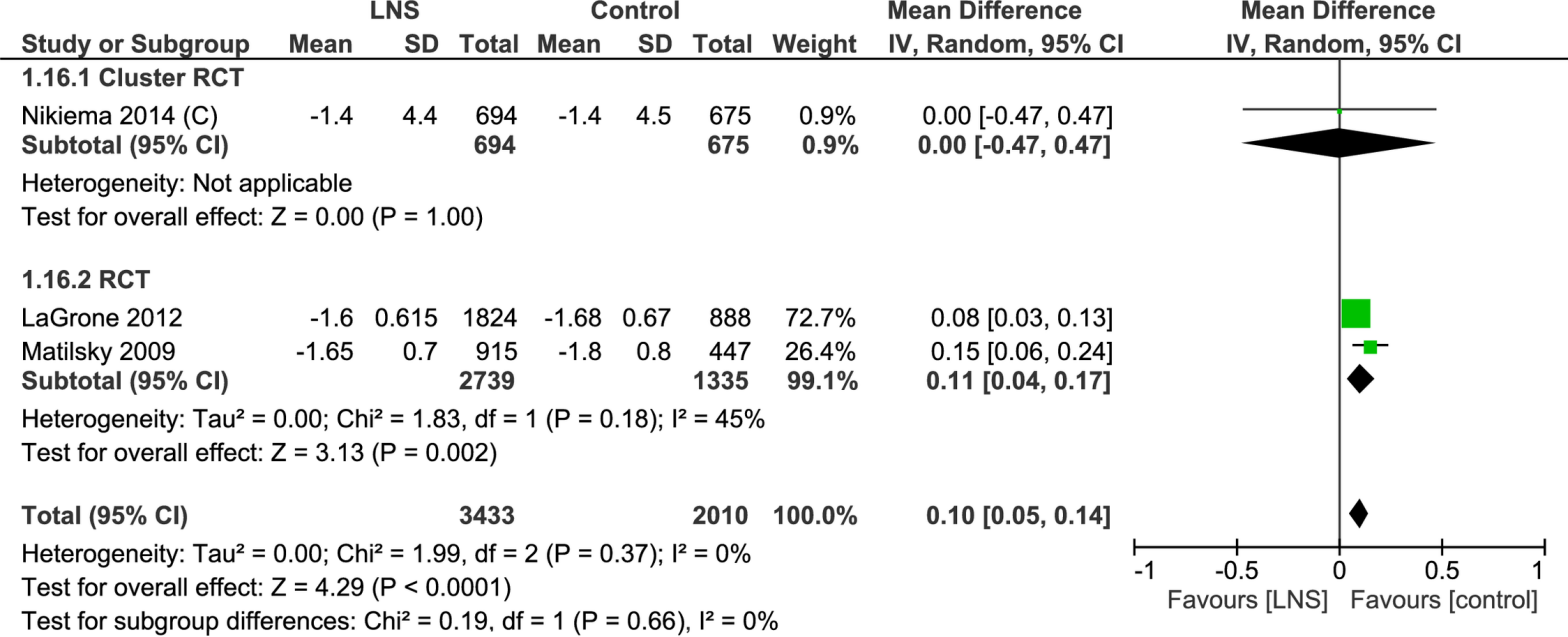
Forest plot: Lipid-based nutrient supplement versus Specially formulated micronutrient fortified foods, outcome: 1.16 weight-for-height z-score End.

**Fig 16:**
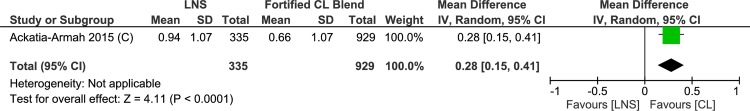
Forest plot: Lipid-based nutrient supplement versus Specially formulated micronutrient fortified foods, outcome: 1.17 Weight-for-length z-score Gain.

Three trials provided weight-for-height z-scores at the end of the study period (Table 3, S8 Table, Analysis 1.16; Fig 15). Of these two were randomized controlled trials and one was a cluster randomized controlled trial. Children receiving lipid-based nutrient supplement had a higher weight-for-height z-score (n = 5442; MD = 0.10, 95% CI 0.05, 0.14). The cluster randomized controlled trial showed no evidence of better effect (n = 1369; MD = 0.00, 95% CI -0.47, 0.47), while the two randomized controlled trials showed a benefit (n = 4074, MD = 0.11, 95% CI = 0.04, 0.17). One trial studied the impact of lipid-based nutrient supplements on the change in weight-for-height z-scores. This cluster randomized controlled trial [35–38] showed that while both groups had improvement in weight-for-height z-scores, the lipid-based nutrient supplements group effect was higher (MD = 0.28, 95% CI0.15, 0.41) (Table 3, S8 Table, Analysis 1.17; Fig 16).

#### Height/ length

**Fig 17:**
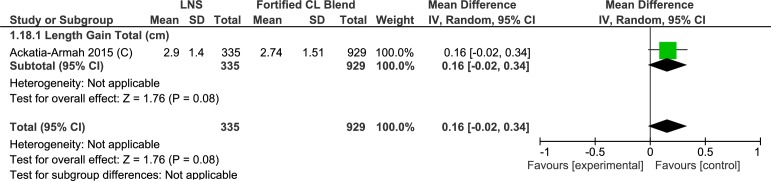
Forest plot: Lipid-based nutrient supplement versus Specially formulated micronutrient fortified foods, outcome: 1.18 Length Gain.

**Fig 18:**
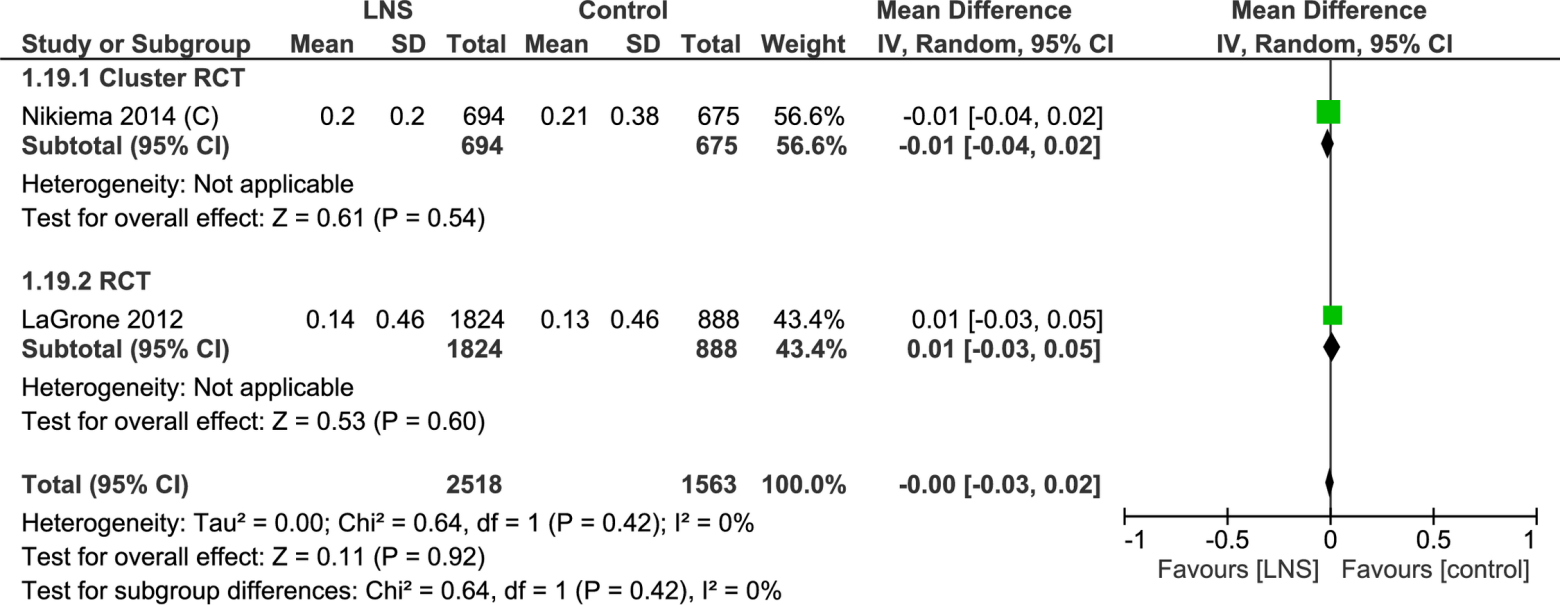
Forest plot: Lipid-based nutrient supplement versus Specially formulated micronutrient fortified foods, outcome: 1.19 Length Gain (mm/d).

One trial studied the change in height (cm). This cluster randomized controlled trial (35–38) showed that while both groups showed increase in height, lipid-based nutrient supplement group had better improvement (MD = 0.16cm, 95% CI-0.02, 0.34) (Table 4, S8 Table, Analysis 1.18; Fig 17). Two trials evaluating rate of change in height (mm/day)found no difference between the two groups (MD = 0.00, 95% CI -0.03, 0.02) (Table 4, Analysis 1.19; Fig 18). Matilsky et al. [22] compared the height gain (mm/day) at each two-week interval and depicted the data as a figure (not included in the meta-analysis). Rates of height gain were similar in children receiving either lipid-based nutrient supplements or corn soy blend.

#### Height-for-age z-scores

**Fig 19:**
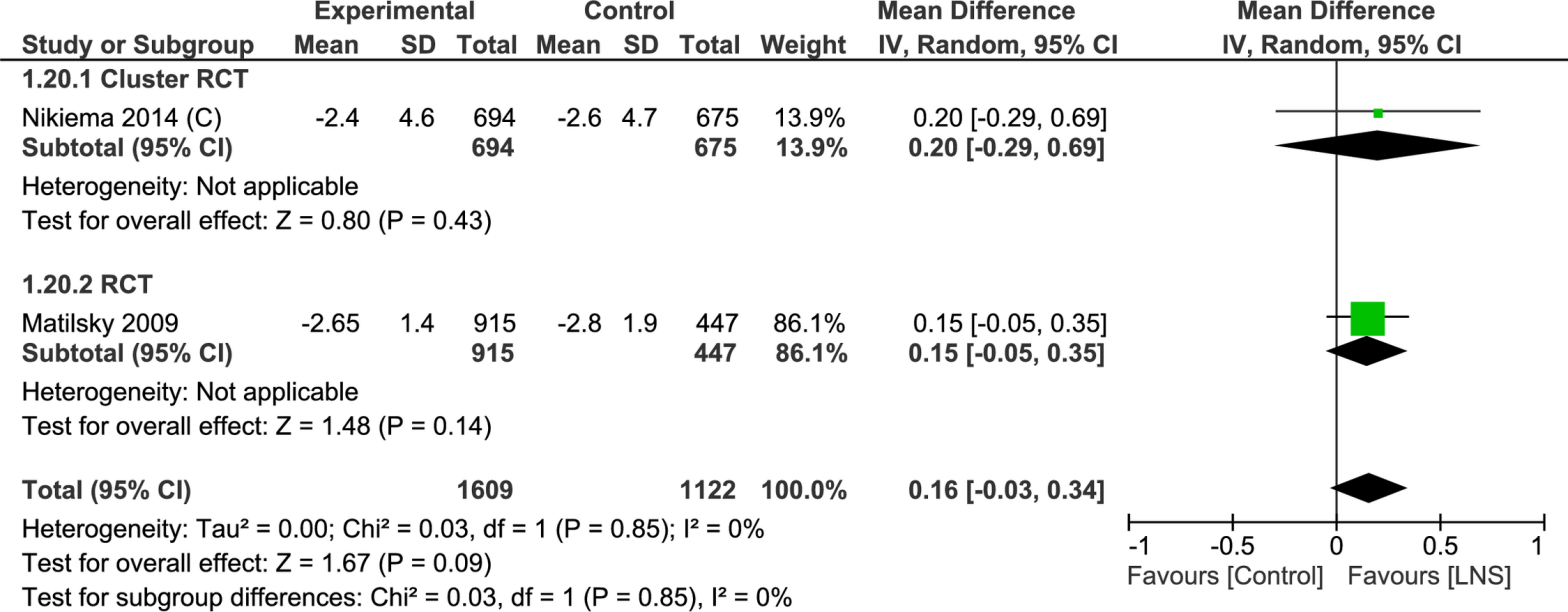
Forest plot: Lipid-based nutrient supplement versus Specially formulated micronutrient fortified foods, outcome: 1.20 height-for-age z-score End.

Two trials (n = 2731) were evaluated this outcome. Height-for-age z-scores at the end of the study period were a little higher with the lipid-based nutrient supplements (MD = 0.16, 95% CI = -0.03, 0.34) (Fig 19). There was one cluster randomized controlled trial (n = 1369, MD = 0.20, 95% CI -0.29, 0.69) and one randomized controlled trial (n = 1362; MD = 0.15, 95% CI -0.05, 0.35) for this analysis.

#### Mid upper arm circumference

**Fig 20:**
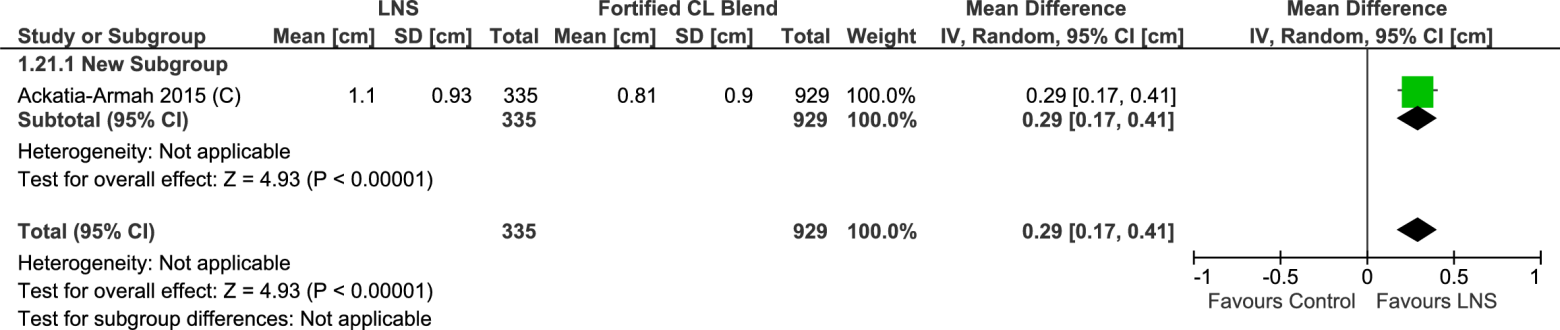
Forest plot: Lipid-based nutrient supplement versus Specially formulated micronutrient fortified foods, outcome: 1.21 mid-upper arm circumference Gain [cm].

**Fig 21:**
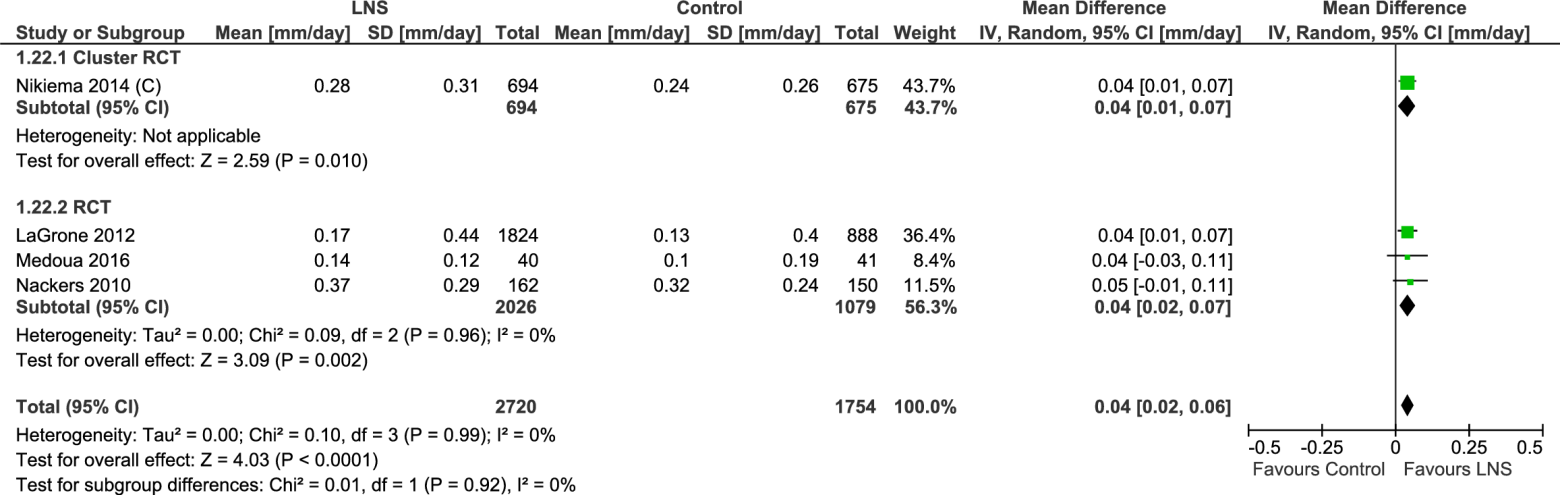
Forest plot: Lipid-based nutrient supplement versus Specially formulated micronutrient fortified foods, outcome: 1.22 Mid-upper arm circumference Gain (mm/day) [mm/day].

One trial studied the change in mid-upper arm circumference (cm). In this cluster randomized controlled trial [35–38] lipid-based nutrient supplement group had higher increase (MD = 0.29 cm, 95% CI 0.17, 0.41) (Table 4, S8 Table, Analysis 1.21; Fig 20). Four trials evaluating rate of change in mid-upper arm circumference (mm/day) showed slightly better improvement with lipid-based nutrient supplement (n = 4474; MD = 0.04, 95% CI 0.02, 0.06) (S8 Table, Analysis 1.22; Fig 21). The cluster randomized controlled trial (MD = 0.04, 95% CI 0.01, 0.07) and three randomized controlled trials showed similar effect sizes (MD = 0.04, 95% CI 0.02, 0.07). Matilsky et al. [22] compared the mid-upper arm circumference gain (mm/day) at each two week interval and depicted the data as a figure (not included in the meta-analysis). Rates of mid-upper arm circumference gain were greater (P< 0.05) with lipid-based nutrient supplement in comparison to corn soy blend, except with soy/peanut Fortified Spread at 6–8 week interval.

#### Default rate

**Fig 22:**
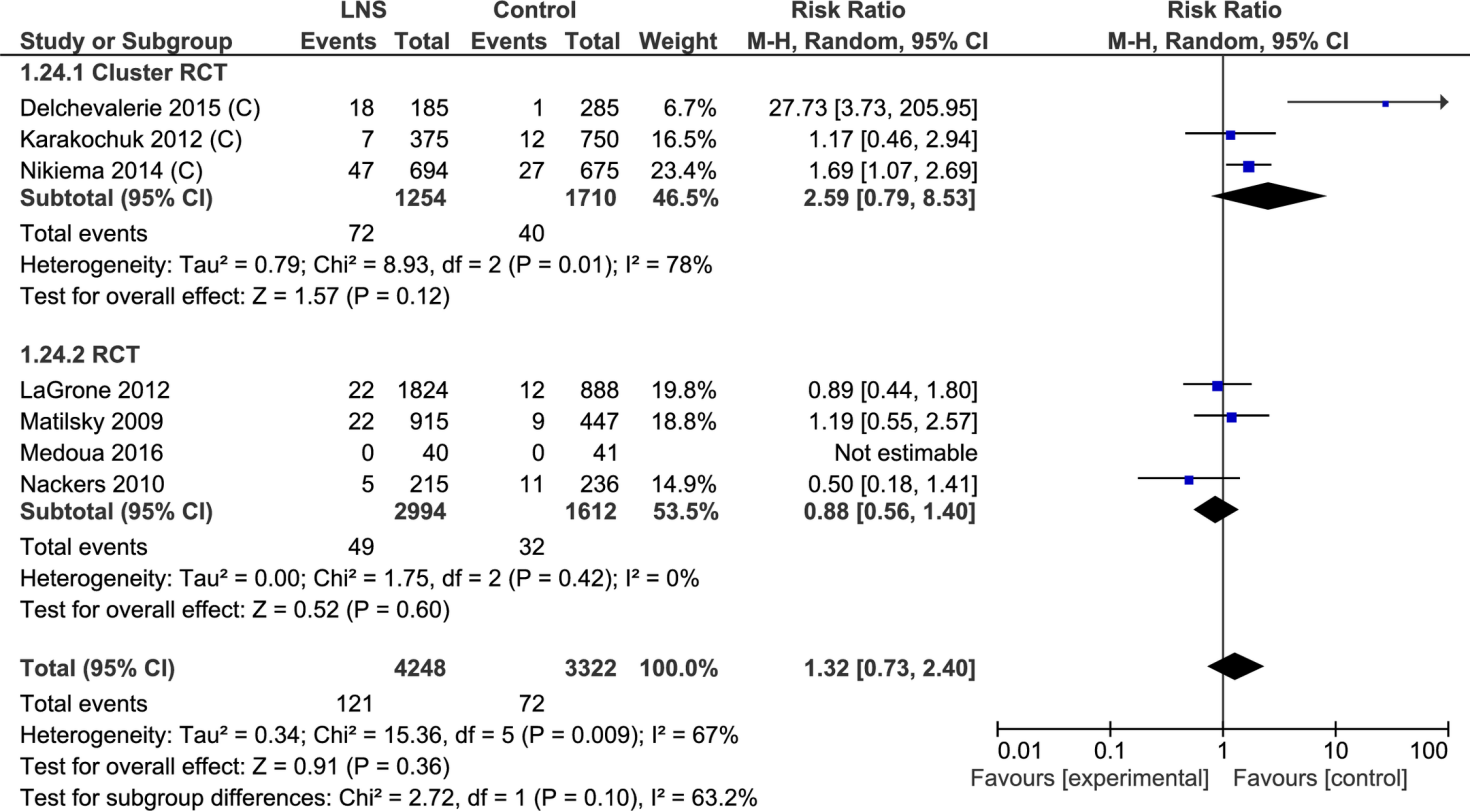
Forest plot: Lipid-based nutrient supplement versus Specially formulated micronutrient fortified foods, outcome: 1.24 Default Rate.

Default rate was reported in 7 trials (7570 study participants). Of these, three were cluster randomized controlled trials and four were randomized controlled trials. Lipid-based nutrient supplements had a higher risk of default (RR = 1.32, 95% CI 0.73, 2.40). There was significant heterogeneity of effect across the studies. Cluster randomized controlled trials showed high risk of default (n = 2964; RR = 2.59, 95% CI 0.79, 8.53), while randomized controlled trials showed lower risk of default with lipid-based nutrient supplements (n = 4606; RR 0.88, 95% CI 0.56, 1.40) (Table 4, S8 Table, Analysis 1.24; Fig 22).

#### Hemoglobin (g/dL)

**Fig 23:**
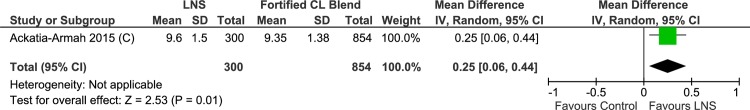
Forest plot: Lipid-based nutrient supplement versus Specially formulated micronutrient fortified foods, outcome: 1.25 Hemoglobin (Final).

**Fig 24:**
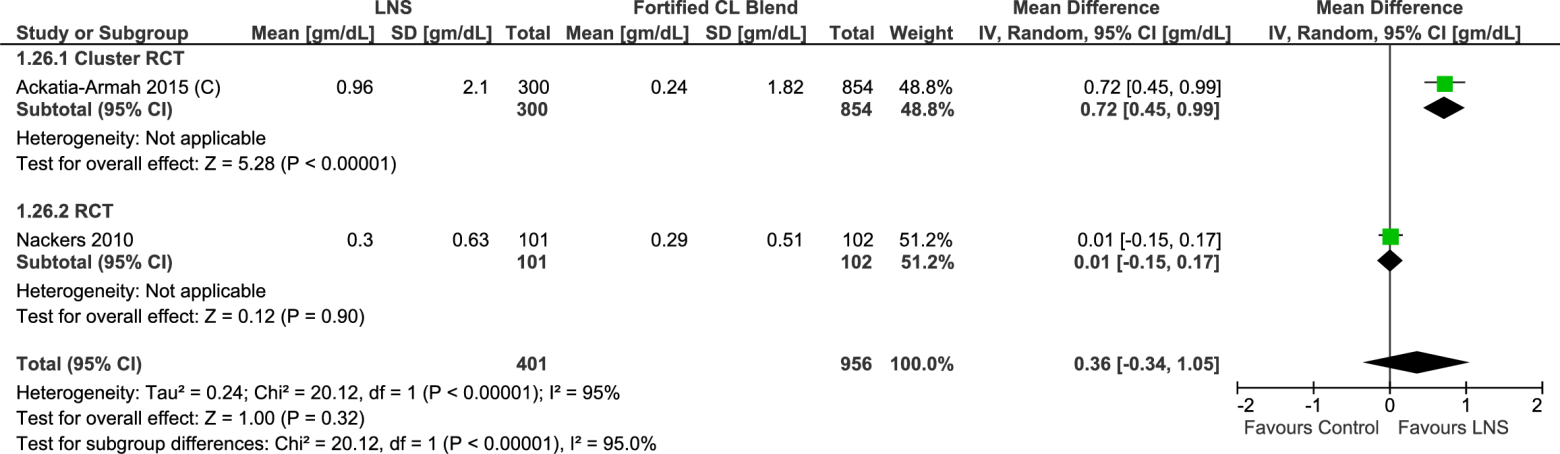
Forest plot: Lipid-based nutrient supplement versus Specially formulated micronutrient fortified foods, outcome: 1.26 Change in Hemoglobin [gm/dL].

In one cluster randomized controlled trial, lipid-based nutrient supplement group showed slightly higher hemoglobin (n = 1154, MD = 0.25 g/dL; 95% CI 0.06 to 0.44) (Table 4, S8 Table, Analysis 1.25; Fig 23). Two trials, one cluster randomized controlled trial and one randomized controlled trial, provided information on the change in hemoglobin levels. The cluster randomized controlled trial showed that although both the lipid-based nutrient supplement and control groups showed a positive change in hemoglobin levels, the effect was higher with lipid-based nutrient supplements (1154 study participants; MD = 0.72 gm/dL, 95% CI 0.45 to 0.99). The one randomized controlled trial included, however, showed similar change in hemoglobin levels in the two groups (203 study participants; MD = 0.01 gm/dL, 95% CI -0.15 to 0.17). On pooling, there was a higher change with the lipid-based nutrient supplement group with imprecise confidence intervals (1357 study participants; MD = 0.36, 95% CI -0.34 to 1.05) (Table 4, S8 Table, Analysis 1.26; Fig 24).

#### Adverse effects

None of the included trials reported systematically on the adverse effects of the dietary supplements provided. All of them reported 'no significant adverse effects'.

#### Morbidity

In one trial, there was a higher risk of vomiting (RR 1.37, 95% CI 1.09, 1.72) and a slightly higher risk of diarrhea (RR 1.10, 95% CI 0.98, 1.24) (Table 5) in the lipid-based nutrient supplement group.

**Table 5:** Summary of findings: Lipid-based nutrient supplements compared to specially formulated micronutrient fortified foods for treatment of moderate acute malnutrition in children (6 months to 59 months) with moderate acute malnutrition.

**Lipid-based nutrient supplement compared to specially formulated micronutrient fortified foods for treatment of moderate acute malnutrition in infants and children 6 months to 59 months with moderate acute malnutrition**						
**Patient or population:** Infants and children 6 to 59 months of age with moderate acute malnutrition **Settings:** **Intervention: Lipid-based nutrient supplement** **Comparison:** Specially formulated micronutrient fortified foods						
**Outcomes**	Illustrative comparative risks* **(95% CI)**		**Relative effect** **(95% CI)**	**No of Participants** **(studies)**	**Quality of the evidence** (GRADE)**	**Comments**
	**Assumed risk**	**Corresponding risk**				
	**Specially Formulated Fortified Foods**	**Lipid-based nutrient supplements**				
**Vomiting**	**Study population**		**RR 1.37** (1.09 to 1.72)	2712 (1 study)	⊕⊝⊝⊝ **very low**^1^^,^^2^	
	**100 per 1000**	**137 per 1000** (109 to 172)				
	**Moderate**					
	**100 per 1000**	**137 per 1000** (109 to 172)				
**Diarrhea**	**Study population**		**RR 1.1** (0.98 to 1.24)	2712 (1 study)	⊕⊝⊝⊝ **very low**^1^^,^^2^	
	**305 per 1000**	**336 per 1000** (299 to 378)				
	**Moderate**					
	**305 per 1000**	**335 per 1000** (299 to 378)				

Footnotes

^1^ Downgraded by 2 for serious indirectness as there was only one study that studied this outcome, conducted in Africa. Extrapolation to other settings and areas where moderate acute malnutrition is prevalent like Asia and Latin America, with different co-morbidities and reasons for malnutrition not possible.

^2^ Downgraded by 1 for imprecision. There are few studies, with few study participants and estimates have wide confidence intervals around the estimate of the effect

*The basis for the **assumed risk** (e.g. the median control group risk across studies) is provided in footnotes. The **corresponding risk** (and its 95% confidence interval) is based on the assumed risk in the comparison group and the **relative effect** of the intervention (and its 95% CI).

**GRADE Working Group grades of evidence

**High quality:** Further research is very unlikely to change our confidence in the estimate of effect.

**Moderate quality:** Further research is likely to have an important impact on our confidence in the estimate of effect and may change the estimate.

**Low quality:** Further research is very likely to have an important impact on our confidence in the estimate of effect and is likely to change the estimate.

**Very low quality:** We are very uncertain about the estimate.

#### Change in body composition

Only one cluster randomized controlled trial [35–38] reported on the change in body composition (total body water, fat mass and % fat mass). At baseline, total body water, fat mass, and percent fat Mass across all study participants was 7.03 ± 1.12 kg, 3.94 ± 0.76 kg, and 29.0 ± 6.32%, respectively. At 12 weeks, fat mass increased by 0.35 kg, 0.29 kg, 0.25 kg, and 0.41 kg (p = 0.02) among children in the Supplementary Plumpy, corn soy blend plus plus, Misola, and locally milled flours groups, respectively; however, the change in percent fat mass did not differ between groups (p = 0.13). Gains in total body water, fat mass, and percent fat mass were greater in children who recovered from moderate acute malnutrition (p<0.0001).

#### Infant and young child feeding practices

There was no detailed data studying the impact of lipid-based nutrient supplementation on infant and young child feeding practices. One trial [35–38] scheduled home visits (not for all included study participants). During the 8 hours of in-home observation (n = 164), > 70% of mothers in all groups breastfed the study child at least once.

#### Intra-household sharing of the provided supplement

In Matilsky et al. [22] caretakers receiving any supplement believed that the foods were a medical treatment and only four of them reported sharing the food with others. During home visits Ackatia-Armah et al. (35–38) noted that the supplement was almost always consumed by the study child (> 94% of observation days) and was shared with other children during fewer than 4% of home visits.

There was no data on cardio-metabolic risk factors, environmental impact of lipid-based nutrient supplementation and cost effectiveness in the included trials.

### Comparisons 2 and 3

We did not identify any studies comparing the use of lipid = based nutrient supplements with no intervention or with improved adequacy of local diet.

### Comparison 4. Lipid-based nutrient supplements versus counselling on feeding best possible local diet—Home-based foods (One study, 1299 participants) (Tables 6 and 7)

One available trial [41] had three arms—lipid-based nutrient supplements, cereal-legume blend and nutritional counselling. We compared the lipid-based nutrient supplements and counselling arms (1299 study participants) (Tables 6 and 7). Children receiving lipid-based nutrient supplements had a higher chance of recovery (RR 1.28, 95% CI 1.18, 1.39). The proportion of children not recovering from moderate acute malnutrition were similar (RR 0.94, 95% CI 0.68, 1.28). Children receiving lipid-based nutrient supplements had 30% lower risk of developing severe acute malnutrition (RR 0.71, 95% CI 0.51, 0.99). Mortality was lower in the lipid-based nutrient supplements group, but with imprecise confidence intervals (RR 0.44, 95% CI 0.11, 1.74). Default was much lower in the lipid-based nutrient supplements group (RR 0.37, 95% CI 0.26, 0.51). Weight gain was higher in the lipid-based nutrient supplements group (0.50 g/kg/day, 95% CI 0.18, 0.82). Weight-for-height z-scores were higher in the lipid-based nutrient supplements group, with imprecise confidence intervals (MD 0.20, 95% CI -0.13, 0.53). There was no difference in the length gain (MD 0.02; 95% CI -0.00, 0.04). Height-for-age z-scores were higher in the lipid-based nutrient supplements group compared to the counselling group (0.50, 95% CI 0.14, 0.86). Mid-upper arm circumference gain was similar in both the groups (MD 0.02, 95% CI -0.02, 0.06).

**Table 6:** Summary of findings: Lipid-based nutrient supplements compared to Counselling for treatment of children (6 months to 59 months) with moderate acute malnutrition.

Lipid-based nutrient supplements **compared to Counselling for treatment of children (6 months to 59 months) with moderate acute malnutrition**						
**Patient or population:** Children (6 months to 59 months) with moderate acute malnutrition **Settings:** **Intervention:** Lipid-based nutrient supplements **Comparison:** Counselling						
**Outcomes**	Illustrative comparative risks* **(95% CI)**		**Relative effect** **(95% CI)**	**No of Participants** **(studies)**	**Quality of the evidence** (GRADE)**	**Comments**
	**Assumed risk**	**Corresponding risk**				
	**Counselling**	**Lipid-based nutrient supplements**				
**Recovery from** moderate acute malnutrition	**Study population**		**RR 1.28** (1.18 to 1.39)	1299 (1 study)	⊕⊝⊝⊝ **very low**^1^^,^^2^	
	**579 per 1000**	**740 per 1000** (683 to 804)				
	**Moderate**					
	**579 per 1000**	**741 per 1000** (683 to 805)				
**No Recovery**	**Study population**		**RR 0.94** (0.68 to 1.28)	1299 (1 study)	⊕⊝⊝⊝ **very low**^1^^,^^2^^,^^3^	
	**111 per 1000**	**104 per 1000** (75 to 142)				
	**Moderate**					
	**111 per 1000**	**104 per 1000** (75 to 142)				
**Deterioration to** severe acute malnutrition	**Study population**		**RR 0.71** (0.51 to 0.99)	1299 (1 study)	⊕⊝⊝⊝ **very low**^1^^,^^2^	
	**116 per 1000**	**82 per 1000** (59 to 115)				
	**Moderate**					
	**116 per 1000**	**82 per 1000** (59 to 115)				
**Mortality**	**Study population**		**RR 0.44** (0.11 to 1.74)	1299 (1 study)	⊕⊝⊝⊝ **very low**^1^^,^^2^^,^^3^	
	**10 per 1000**	**4 per 1000** (1 to 17)				
	**Moderate**					
	**10 per 1000**	**4 per 1000** (1 to 17)				
**Defaulter**	**Study population**		**RR 0.37** (0.26 to 0.51)	1299 (1 study)	⊕⊝⊝⊝ **very low**^1^^,^^2^	
	**185 per 1000**	**68 per 1000** (48 to 94)				
	**Moderate**					
	**185 per 1000**	**68 per 1000** (48 to 94)				

Footnotes

^1^ Downgraded by 1 for Risk of Bias. Only one trial included which was at high risk of bias for blinding of participants and personnel, blinding of outcome assessment, allocation concealment and baseline balance between clusters.

^2^ Downgraded by 2 for indirectedness. Only one study, from Africa, from rural setting that cannot be extrapolated to other settings, countries or populations

^3^ Downgraded by 1 for imprecision: Sample size low with wide imprecise confidence interval

*The basis for the **assumed risk** (e.g. the median control group risk across studies) is provided in footnotes. The **corresponding risk** (and its 95% confidence interval) is based on the assumed risk in the comparison group and the **relative effect** of the intervention (and its 95% CI).

**GRADE Working Group grades of evidence

**High quality:** Further research is very unlikely to change our confidence in the estimate of effect.

**Moderate quality:** Further research is likely to have an important impact on our confidence in the estimate of effect and may change the estimate.

**Low quality:** Further research is very likely to have an important impact on our confidence in the estimate of effect and is likely to change the estimate.

**Very low quality:** We are very uncertain about the estimate.

**Table 7:** Summary of findings: Lipid-based nutrient supplements compared to Counselling for treatment of children (6 months to 59 months) with moderate acute malnutrition.

Lipid-based nutrient supplements **compared to Counselling for treatment of children (6 months to 59 months) with** moderate acute malnutrition						
**Patient or population:** Children (6 months to 59 months) with moderate acute malnutrition **Settings:** **Intervention:** Lipid-based nutrient supplements **Comparison:** Counselling						
**Outcomes**	Illustrative comparative risks* **(95% CI)**		**Relative effect** **(95% CI)**	**No of Participants** **(studies)**	**Quality of the evidence** (GRADE)**	**Comments**
	**Assumed risk**	**Corresponding risk**				
	**Counselling**	**Lipid-based nutrient supplements**				
**Weight Gain**		The mean weight gain in the intervention groups was **0.5 higher** (0.18 to 0.82 higher)		1299 (1 study)	⊕⊝⊝⊝ **very low**^1^^,^^2^^,^^3^	
**Weight Gain—Weight Gain (g/kg/d)**		The mean weight gain—weight gain (g/kg/d) in the intervention groups was **0.5 higher** (0.18 to 0.82 higher)		1299 (1 study)	⊕⊝⊝⊝ **very low**^1^^,^^2^^,^^3^	
Weight-for-height z-score **End**		The mean weight-for-height z-score end in the intervention groups was **0.2 higher** (0.13 lower to 0.53 higher)		1299 (1 study)	⊕⊝⊝⊝ **very low**^1^^,^^2^	
**Length Gain**		The mean length gain in the intervention groups was **0.02 higher** (0 to 0.04 higher)		1299 (1 study)	⊕⊝⊝⊝ **very low**^1^^,^^2^	
**Length Gain—Length Gain (mm/d)**		The mean length gain—length gain (mm/d) in the intervention groups was **0.02 higher** (0 to 0.04 higher)		1299 (1 study)	⊕⊝⊝⊝ **very low**^1^^,^^2^	
**Height-for-age z-score End**		The mean height-for-age z-score end in the intervention groups was **0.5 higher** (0.14 to 0.86 higher)		1299 (1 study)	⊕⊝⊝⊝ **very low**^1^^,^^2^^,^^3^	
**Mid-upper arm circumference Gain**		The mean mid-upper arm circumference gain in the intervention groups was **0.02 higher** (0.02 lower to 0.06 higher)		1299 (1 study)	⊕⊝⊝⊝ **very low**^1^^,^^2^	
**Mid-upper arm circumference Gain—Mid-upper arm circumference Gain (mm/d)**		The mean mid-upper arm circumference gain—mid-upper arm circumference gain (mm/d) in the intervention groups was **0.02 higher** (0.02 lower to 0.06 higher)		1299 (1 study)	⊕⊝⊝⊝ **very low**^1^^,^^2^	

Footnotes

^1^ Downgraded by 1 for Risk of Bias. Only one trial included which was at high risk of bias for blinding of participants and personnel, blinding of outcome assessment, allocation concealment and baseline balance between clusters.

^2^ Downgraded by 2 for indirectedness. Only one study, from Africa, from rural setting that cannot be extrapolated to other settings, countries or populations

^3^ Downgraded by 1 for imprecision: Sample size low with wide imprecise confidence interval

*The basis for the **assumed risk** (e.g. the median control group risk across studies) is provided in footnotes. The **corresponding risk** (and its 95% confidence interval) is based on the assumed risk in the comparison group and the **relative effect** of the intervention (and its 95% CI).

**GRADE Working Group grades of evidence

**High quality:** Further research is very unlikely to change our confidence in the estimate of effect.

**Moderate quality:** Further research is likely to have an important impact on our confidence in the estimate of effect and may change the estimate.

**Low quality:** Further research is very likely to have an important impact on our confidence in the estimate of effect and is likely to change the estimate.

**Very low quality:** We are very uncertain about the estimate.

No subset or sensitivity analyses was possible for this comparison.

## Discussion

### Summary of main results

We identified nine eligible controlled trials (4 cluster randomized controlled trials, 4 randomized controlled trials and 1 non-randomized controlled trial), with 9270 study participants. All nine included trials compared lipid-based nutrient supplements with specially formulated fortified foods (cereal legume blends in eight trials and United Nations Food supplement in one) (Summary of Findings Tables 3, 4 and 5). One study compared lipid-based nutrient supplements to nutritional counselling (Tables 6, and 7). No studies were identified that compared lipid-based nutrient supplements with either no intervention or improved adequacy of local foods.

The provision of lipid-based nutrient supplements, in comparison to specially formulated foods, improved the recovery rate by 8% (RR 1.08; 95% CI 1.02 to 1.14, low quality evidence) and decreased the chances of no recovery by 30% (RR 0.70; 95% CI 0.58 to 0.85, low quality evidence). The risk of deterioration into severe acute malnutrition decreased by 13% (RR 0.87; 95% CI 0.73 to 1.03,low quality evidence). There was no impact on overall mortality (RR 0.91, 95% CI 0.54 to 1.52,very low quality evidence) or the default rate (RR 1.32; 95% CI 0.73 to 2.4,low quality evidence). There was higher weight gain (MD 0.62 g/kg/d higher; 95% CI 0.18 to 1.06), weight-for-height z-score (MD 0.10 higher; 95% CI 0.05, 0.14), height-for-age z-score (MD 0.16; 95% CI -0.03, 0.34) and mid-upper arm circumference gain (MD 0.04 mm/d; 95% CI 0.02, 0.06), (low or very low quality evidence). A high relapse rate was noted following discharge, and it was not affected by the type of supplement provided. Lipid-based nutrient supplements had a beneficial effect on hemoglobin (low to very low quality evidence). One study showed increased risk of vomiting and diarrhea with lipid-based nutrient supplements. Subset analyses suggested higher recovery rates with greater amounts of calories provided and with ready-to-use therapeutic foods in comparison to ready-to-use supplementary foods.

Only one study compared lipid-based nutrient supplements and nutritional counselling. The quality of evidence for this comparison was very low (Tables 6, and 7). Use of lipid-based nutrient supplements was associated with 28% higher chance of recovery, lower risk of deteriorating into severe acute malnutrition and lower default rate. There was no impact on mortality and no recovery. There was some benefit on weight gain and height-for-age z-score, while there was no effect on weight-for-height z-score, length gain and mid-upper arm circumference.

### Overall completeness and applicability of evidence

One major limitation of the review is that all the included trials are from Africa. There are significant regional variations in the prevalence of moderate acute malnutrition and the etiological factors contributing to it. The largest prevalence of moderate acute malnutrition is seen in South Asia (17%) followed by Africa (8 to 11%) [73]. The larger prevalence of malnutrition in South Asia still remains an enigma. Some postulated etiologies leading up to malnutrition (i.e., poverty, food insecurity, socio-economic disparities, famines or war) seem to operate to a greater extent in sub-Saharan Africa than in South Asia. This may explain the effectiveness of food supplements in treating malnutrition in this region. Some investigators on the other hand attribute the higher malnutrition rates in South Asia to other factors like lower birth weight, poorer sanitation and hygiene and poor feeding practices [74]. Since the profile of malnutrition in the two areas is so disparate, it may not be pragmatic to directly extrapolate these findings from one geographical region to the other.

Infectious diseases like tuberculosis and malaria, that have an impact on etiology and prevalence of moderate acute malnutrition, were not evaluated by any of the included trials. HIV status is likely to have an influence on causation of malnutrition as well as response to therapy. This issue was also inadequately addressed by the investigators. Only one trial [39, 40] studied the impact of HIV infection and antiretroviral therapy on recovery from moderate acute malnutrition. There is thus a need to comprehensively evaluate the impact of HIV and other infections.

None of the studies reported directly on social determinants of health suggesting that variables and analyses related to equity are not commonly considered by lipid-based nutrient supplement trials. Overlooking these factors leads to paucity of evidence on how inequities are identified and addressed, and how interventions can contribute to mitigate or exacerbate them.

Moderate acute malnutrition is a condition of multifactorial etiology. It is unlikely that administration of food supplements alone without addressing the issues of food security, poverty, adequate sanitation and drinking water, and infectious morbidities would lead to long term amelioration of the problem. To be considered an effective strategy, it is important to evaluate the effectiveness and relapse rate of the study populations over a longer period of time. However, only two trials evaluated the long-term relapse rate.

Comparison of the products appropriately (in terms of equal calories provided to the study and control groups) has been an important methodological issue in a large number of included trials. While three trials provided higher amounts to children receiving cereal legume blends, one trial Vanelli et al. [45] provided ready-to-use therapeutic foods in addition to food supplement being provided to the two comparison groups. Deriving conclusions can be tricky when the caloric content of the supplement provided varies in the treatment and control groups.

It has been postulated that inclusion of dairy in the nutrient supplements may enhance recovery rates. We found some evidence of a higher effect size with lipid-based nutrient supplements containing dairy products (Table 4, Analysis 1.7). However, two of the three studies providing dairy based lipid-based nutrient supplements used ready-to-use therapeutic foods (implying more calorie dense foods) to the malnourished children. In the single study with a direct comparison between milk and soy based lipid-based nutrient supplements, the recovery rates were comparable [22]. There is thus no good evidence of better recovery rates with dairy based supplements.

Both lipid based nutrient supplement and fortified foods provide micronutrients, but the LNS group (2 trials) reported increase in the hemoglobin levels from baseline. The lower rise in specially formulated fortified foods when compared to LNS (very low quality evidence) may be due to lower absorption of iron because of difference in the iron salts or the inhibitory effect of cereal based vehicle in specially formulated fortified foods.

Although all studies reported ‘no significant adverse effects’, one study showed increased risk of vomiting and marginally increased risk of diarrhea with the use of lipid-based nutrient supplements [39, 40]. Diarrhea may represent an adverse effect of the lipid-based nutrient supplements or may be reflective of an increase in the risk of gastrointestinal infection. One of the postulated advantages of lipid-based nutrient supplements is a reduced risk of gastrointestinal infections because it does not require water for constitution. This paradoxical finding therefore needs to be addressed with better designed studies in future.

Overall, currently available evidence is far from complete for taking robust decisions on applicability. The trials awaiting publication and the ongoing ones (S6 Table, S7 Table) may address some of these issues.

### Quality of the evidence

Except for three trials [41, 45, 48], the risk of bias for the included trials was moderate to low (S2 Table, Figs 2 and 3). The quality of evidence for all the evaluated outcomes was assessed using the GRADE methodology [75]. The overall quality of evidence for the various outcomes in the two comparisons varied from very low to low.

### Potential biases in the review process

We could not definitely exclude a publication bias because of paucity of included trials.

### Agreements and disagreements with other studies or reviews

We identified three previously conducted systematic reviews [32, 76, 77] evaluating the effectiveness of lipid-based nutrient supplements for the treatment of moderate acute malnutrition in children. Interestingly, all three reviews were published in the same year. All three reviews identified five trials for inclusion and reported 10–11% better recovery rate with the use of LNS when compared with fortified blended foods. Our review identified more studies for the analyses, with similar results. There were some differences from the Cochrane review [32] in the Risk of Bias for the included trials and the grading of the quality of evidence. We did stratified analyses for different study designs in the included trials, i.e., randomized controlled trials, cluster randomized controlled trials and non randomized controlled trials. We were also able to do more detailed subset and sensitivity analyses to explore heterogeneity.

### Implications for practice

Evidence restricted to the African regions suggests that lipid-based nutrient supplements may be slightly more effective than specially formulated fortified foods or nutritional counselling in recovery from moderate acute malnutrition, lowering the risk of deterioration into severe acute malnutrition, and improving weight gain. However, there is little impact on overall mortality or the default rate. Further, there is little or no information on adverse effects, body composition changes, relative cost-effectiveness and equity aspects of different approaches for formulating policy. It may thus be tricky to unequivocally recommend the routine use of lipid-based nutrient supplements in preference to other approaches or products for the treatment of moderate acute malnutrition in children (6 to 59 months age) in all settings.

### Implications for research

There is need for better designed studies using standard definitions and robust methodology from varied settings to evaluate the efficacy and safety of lipid-based nutrient supplements in comparison to other dietary therapies and nutrition counselling. All the studies are from Africa and it is possible that the profile and response of moderate acute malnutrition in South Asian settings, with the greatest burden, may vary. Surprisingly there is little evidence on adverse effects; the potential risk of increased diarrhoea in some studies needs further evaluation. There is an urgent need to generate evidence for other important outcomes necessary for decision making, including the effect on feeding practices, intrahousehold sharing of supplements, body composition changes, micronutrient status, cognition, equity aspects and cost effectiveness.

## Supporting information

S1 Appendix Appendix 1. (DOCX)

S1 Table Prisma checklist. (DOCX)

S2 Table Characteristics of included studies. (DOCX)

S3 Table PROGRESS plus. (DOCX)

S4 Table Account of PROGRESS. (DOCX)

S5 Table Characteristics of excluded studies. (DOCX)

S6 Table Characteristics of studies awaiting classification. (DOCX)

S7 Table Characteristics of ongoing studies. (DOCX)

S8 Table Analysis—LNS versus specially formulated foods. (DOCX)

S9 Table Analysis–LNS versus counselling. (DOCX)

S10 Table Sensitivity analyses for recovery from moderate acute malnutrition. (DOCX)
