# Supplementary material for: Lipid based nutrient supplements (LNS) for treatment of children (6 months to 59 months) with moderate acute malnutrition (MAM): A systematic review
Source: PLoS One. 2017 Sep 21;12(9):e0182096. doi: 10.1371/journal.pone.0182096 (PMC5608196; doi:10.1371/journal.pone.0182096)
Supplement: S1 Appendix — (DOCX) [file pone.0182096.s001.docx]

## Appendix 1. Medline Search Strategy

Search Strategy for Medline

Mesh Words

Nutrition Disorders

Child Nutrition Disorders

Infant Nutrition Disorders

Malnutrition

Wasting Syndrome

Protein Energy Malnutrition

Kwashiorkor

Weight Loss

Emaciation

Cachexia

Nutritional Status

Text Words

Moderate Acute Malnutrition

Acute Malnutrition

MAM

Wasting or wasted

Stunting or stunted

Undernutrition or undernourished

Underweight

Mesh Words

Child, Preschool

Infant

Text Words

Child or Children

Infant

Under-5 Children

Mesh Words

Nutrition Therapy

Diet Therapy

Nutritional Support

Food, Formulated

Dietary Supplements

Foods, specialized

Weight Gain

Text Words

LNS

RUTF

RTUF

RUF

RUSF

Medical Nutrition Therapy

Therapeutic Nutrition Product

Mesh Words

Clinical Trial

Controlled Clinical Trial

Randomized Controlled Trial

Pragmatic Clinical Trial

EMBASE

(Nutrition Disorder or Kwashiorkor or Protein Calorie Malnutrition or Anthropometry or Nutritional Status or Wasting Syndrome or Malnutrition or Cachexia).sh. or (Moderate Acute Malnutrition or Acute Malnutrition or MAM or Wasting or wasted or Stunting or stunted or Undernutrition or undernourished or Underweight).tw.

AND

(Child or Children or Infant or Under-5 Children).sh.

AND

(LNS or RUTF or RUF or RUSF or medical nutritional therapy or therapeutic nutrition product).tw. or (Nutrition Therapy or Diet Therapy or Nutritional Support or Food, Formulated or Dietary Supplements or Foods, specialized or Weight Gain).sh.

AND

(Clinical Trial or Controlled Clinical Trial or Randomized Controlled Trial or Pragmatic Clinical Trial).sh.

CENTRAL

'LNS or RUTF or RUF or RUSF or medical nutritional therapy or therapeutic nutrition product or Nutrition Therapy or Diet Therapy or Nutritional Support or Food, Formulated or Dietary Supplements or Foods, specialized or Weight Gain in Title, Abstract, Keywords and Nutrition Disorder or Kwashiorkor or Protein Calorie Malnutrition or Anthropometry or Nutritional Status or Wasting Syndrome or Malnutrition or Cachexia or Moderate Acute Malnutrition or Acute Malnutrition or MAM or Wasting or wasted or Stunting or stunted or Undernutrition or undernourished or Underweight in Title, Abstract, Keywords and Child or Children or Infant or Under-5 Children in Title, Abstract, Keywords in Trials'

Web of Science

(Nutrition Disorder or Kwashiorkor or Protein Calorie Malnutrition or Anthropometry or Nutritional Status or Wasting Syndrome or Malnutrition or Cachexia or Moderate Acute Malnutrition or Acute Malnutrition or MAM or Wasting or wasted or Stunting or stunted or Undernutrition or undernourished or Underweight) *AND* **TOPIC:** (LNS or RUTF or RUF or RUSF or medical nutritional therapy or therapeutic nutrition product or Nutrition Therapy or Diet Therapy or Nutritional Support or Food, Formulated or Dietary Supplements or Foods, specialized or Weight Gain) *AND* **TOPIC:** (Child or Children or Infant or Under-5 Children) *AND* **TOPIC:** (Clinical Trial or Controlled Clinical Trial or Randomized Controlled Trial or Pragmatic Clinical Trial)

lilacs

**Nutrition Disorder or Kwashiorkor or Protein Calorie Malnutrition or Anthropometry or Nutritional Status or Wasting Syndrome or Malnutrition or Cachexia or Moderate Acute Malnutrition or Acute Malnutrition or MAM or Wasting or wasted or Stunting or stunted or Undernutrition or undernourished or Underweight [Words] and LNS or RUTF or RUF or RUSF or medical nutritional therapy or therapeutic nutrition product or Nutrition Therapy or Diet Therapy or Nutritional Support or Food, Formulated or Dietary Supplements or Foods, specialized or Weight Gain [Words] and Child or Children or Infant or Under-5 Children [Words]**

**POPLine**

(Nutrition Disorder or Kwashiorkor or or Anthropometry or Wasting Syndrome or Malnutrition) AND (LNS or RUTF or RUSF or RUF or Diet Therapy or Nutrition Therapy)
