# Supplementary material for: Lipid based nutrient supplements (LNS) for treatment of children (6 months to 59 months) with moderate acute malnutrition (MAM): A systematic review
Source: PLoS One. 2017 Sep 21;12(9):e0182096. doi: 10.1371/journal.pone.0182096 (PMC5608196; doi:10.1371/journal.pone.0182096)
Supplement: S2 Table — (DOCX) [file pone.0182096.s003.docx]

## S2: Characteristics of included studies

### Ackatia-Armah 2015 (C)

| **Methods** | Cluster Randomized Controlled Trial |
| --- | --- |
| **Participants** | Children 6 to 35 months old residing in Diolia Health District of Mali. The study was based in 12 community centres and their surrounding communities, which were selected based on their accessibility, population size, and history of collaboration with external projects.  Inclusion Criteria: Weight-for-length z-score < -2 and > -3 or mid-upper arm circumference < 12.5 cm and > 11.5 cm or weight-for-length z-score < 80% and > 70% of the National Center for Health Statistics median or mid-upper arm circumference < 12.0 and > 11.0 cm, without edema.  Exclusion Criteria: Severe anemia (hemoglobin <50 g/L); severe acute malnutrition; other acute illnesses requiring inpatient treatment; congenital abnormalities or underlying chronic diseases, including known HIV infection that might interfere with nutritional recovery; or a history of allergy to peanuts or previous serious allergic reactions to any substance and requiring emergency medical care. |
| **Interventions** | Group 1: Ready-to-use supplementary foods  Group 2: Corn-soy blend plus plus (supplied by the World Food Program), a specially formulated refined cereal-legume-milk blend for children with moderate acute malnutrition  Group 3: Misola (supplied by Misola, Mali), a less-refined micronutrient-fortified cereal-legume blend  Group 4: A less-refined cereal-legume milled flour mix  Each supplement was supplied in an amount to provide 500 kcal/d, which was to be consumed in addition to the usual home diet for 12 weeks. |
| **Outcomes** | 1. Weight Gain  2. Recovery from moderate acute malnutrition  3. No recovery  4. Deterioration to severe acute malnutrition  5. Length Gain  6. Weight-for-length z-score change  7. Mid-upper arm circumference Change  8. Hemoglobin (Final)  9. Change in Hemoglobin |
| **Notes** | Group 2,3 and 4 were combined since they all received a fortified cereal legume blend. |

#### Risk of bias table

| **Bias** | **Authors' judgement** | **Support for judgement** |
| --- | --- | --- |
| Random sequence generation (selection bias) |  | CSComs were randomly assigned to one of 4 dietary interventions within each stratum at the beginning of the study, and they were randomly reassigned, within stratum, to a different dietary group after the first 3 rounds of screening. |
| Allocation concealment (selection bias) |  | Not mentioned |
| Blinding of participants and personnel (performance bias) |  | Because of the different physical nature of the dietary supplements and product-specific counseling on their preparation and serving methods, the study was not masked. |
| Blinding of outcome assessment (detection bias) |  | Not Mentioned |
| Incomplete outcome data (attrition bias) |  | "Of the 1264 children originally enrolled, 1175 (93.3%) completed the full 12 weeks of treatment and scheduled observations. The overall decline in participation did not differ by study group". |
| Selective reporting (reporting bias) |  | None detected |
| Other bias |  | None detected |
| Recruitment Bias |  | Study participants recruited after the randomization of the community centres |
| Loss of clusters |  | No loss of clusters |
| Baseline Imbalance |  | All clusters lie in one health district, served by the same district hospital, with similar accessibility, and population size. |
| Incorrect Analysis |  | To account for the cluster sampling design, the sample size calculated was multiplied by 1.5, assuming a minimal degree of intracluster correlation of major outcomes. In the analyses, groups were compared by using ANCOVA models after adjusting for cluster site (community health clinic), baseline anthropometric characteristic (weight, length, mid-upper arm circumference, and weight-for-height z-score), age, sex, food security status, and month of enrolment. |
| Comparison with individual randomized controlled trial |  |  |

### Delchevalerie 2015 (C)

| **Methods** | Unblinded, cluster randomized controlled trial |
| --- | --- |
| **Participants** | The trial was conducted from April 2007 to April 2008 in a rural setting of Bo district in Sierra Leone. The prevalence of wasting in the area was 9.2% among children aged ≤ 24 months.  Inclusion Criteria  1) Weight-for-height of the reference median at 70-79% without edema  2. Approval from the caretaker. |
| **Interventions** | Group 1: Corn soy blend-oil premix (226g corn soy blend, 37g oil, and 23g sugar) at 1277 kcal/day  Group 2: Ready-to-use supplementary foods (Supplementary-plumpy®) at 1000 kcal/day  Children from both groups received a standard treatment protocol including measles vaccination, vitamin A supplementation, folic acid supplementation and deworming. As part of the clinical examination, a rapid malaria diagnostic test (Paracheck-Pf®) was performed at admission,and artemisinin combination therapy was given when indicated. |
| **Outcomes** | 1. Recovery (weight-for-height of the reference median > 85% for two consecutive weeks)  2. Weight gain  3. Duration to recovery  4. Death  5. Default (Supplementary Feeding Centre absence for two consecutive weeks)  6. Transfer to in-patient facility (bilateral edema, mid-upper arm circumference< 110 mm, weight-for-height of the reference median < 70%, or medical complications requiring hospitalization)  7. Number of morbidity episodes during the treatment period and six months thereafter  8. Relapse following discharge |
| **Notes** |  |

#### Risk of bias table

| **Bias** | **Authors' judgement** | **Support for judgement** |
| --- | --- | --- |
| Random sequence generation (selection bias) |  | Randomized trial |
| Allocation concealment (selection bias) |  | Not mentioned |
| Blinding of participants and personnel (performance bias) |  | Unblinded trial |
| Blinding of outcome assessment (detection bias) |  | Unblinded trial |
| Incomplete outcome data (attrition bias) |  | Data regarding all outcomes reported for all included children. |
| Selective reporting (reporting bias) |  | Not detected |
| Other bias |  | Not detected |
| Recruitment Bias |  | Not mentioned |
| Loss of clusters |  | No loss of clusters |
| Baseline Imbalance |  | As socio-economic conditions across Supplementary Feeding Centres differed, a two-step procedure was used to ensure similar representation for the poorest areas in both groups. Centers were first categorized according to their location in either mining or agricultural areas, followed by randomization of the treatment by geographical area. |
| Incorrect Analysis |  | Author clarification sought, no response |
| Comparison with individual randomized controlled trial |  |  |

### Karakochuk 2012 (C)

| **Methods** | Cluster-randomized effectiveness trial embedded in a conventional supplementary feeding program |
| --- | --- |
| **Participants** | The current study presents data from 10 health centers and health posts in the northern region of the Sidama zone, Ethiopia, where there was a “priority 2” level of food and nutrition insecurity and where no other food assistance programs were implemented. Ethiopian children aged 6 to 60 months were screened by midupper arm circumference. Children with mid-upper arm circumference ,135 mm were referred for second-stage assessment using weight-for-height > 70 to < 80% according to National Center for Health Statistics growth references.  **Exclusion criteria**  1) children with mid-upper arm circumference **<** 110 mm, bilateral pitting edema, or other complications;  2) children transferred from therapeutic feeding programs; and  3) children with any condition preventing safe ingestion of either food (i.e., peanut allergy). |
| **Interventions** | Group 1: Ready-to-use supplementary foods (Supplementary Plumpy; Nutriset)  Group 2: Corn soy blend  Children received biweekly rations of either corn soy blend or ready-to-use supplementary foods for 16 weeks. The daily rations were equivalent to 300 g corn soy blend and 32 g vegetable oil (1413 kcal, 47 g protein) or 92 g ready-to-use supplementary foods (500 kcal, 13 g protein). The quantity of the corn soy blend ration was purposely higher because of expected household food sharing of the corn soy blend ration. |
| **Outcomes** | 1. Recovery (2 consecutive measurements of weight-for-height > 85% within a 16 weeks time period)  2. Default (Child is absent for 2 visits or does not return to the program and is lost to follow-up)  3. Non-response (Child does not reach recovery after 16 wk of treatment)  4. Transfer to inpatient (weight-for-height decreases to < 70% or child becomes ill or has complications and requires transfer to inpatient care  5. Mortality |
| **Notes** |  |

#### Risk of bias table

| **Bias** | **Authors' judgement** | **Support for judgement** |
| --- | --- | --- |
| Random sequence generation (selection bias) |  | Cluster randomized trial |
| Allocation concealment (selection bias) |  | Blinded draw done from an opaque bag. |
| Blinding of participants and personnel (performance bias) |  | Not done |
| Blinding of outcome assessment (detection bias) |  | Not done |
| Incomplete outcome data (attrition bias) |  | Data were excluded from the analysis for 43 children (< 1%) because of incomplete or lost files. |
| Selective reporting (reporting bias) |  | Not detected |
| Other bias |  | The quantity of the corn soy blend ration was much higher than ready-to-use supplementary foods, assuming that there would be significant household food sharing of the corn soy blend ration. |
| Recruitment Bias |  | Recruitement of study participants done after the randomization of the clusters |
| Loss of clusters |  | No loss of clusters. |
| Baseline Imbalance |  | Before the two districts were selected for the study, livelihood and food security profiling was conducted to ensure comparability of populations and food security status. The two districts bordered each other and were of comparable size with very similar environments, populations, access to services, and food security levels. Similar cash crops (chat, enset, and livestock) and food crops (enset and barley) were prevalent in both districts. In addition, five supplementary feeding program sites were purposely and geographically chosen in each of the two districts so that all beneficiaries had equal access. |
| Incorrect Analysis |  | A design effect of 1.5 was applied to compensate for variability among and between districts. Robust Standarad Errors were used to account for the clustered data. |
| Comparison with individual randomized controlled trial |  |  |

### LaGrone 2012

| **Methods** | Randomized Controlled Trial |
| --- | --- |
| **Participants** | Participants came from families of subsistence farmers. An estimated 10 to 23% of rural pregnant Malawians are HIV-positive. Considering rates of vertical transmission of HIV, the projected childhood HIV prevalence is 0.2 to2%  **Inclusion Criteria**  Children aged 6 to 59 mo with moderate acute malnutrition (weight-for-height z-score < -2 and > -3z without bipedal edema).  **Exclusion Criteria:**  1. Simultaneous involvement in another research trial or supplementary feeding program,  2. A chronic debilitating illness (not including HIV or tuberculosis)  3. A history of peanut allergy  4. Received therapy for acute malnutrition within 1 month before presentation |
| **Interventions** | 1. Corn soy blend plus plus  2. Soy ready-to-use supplementary foods (Locally Produced)  3. Soy/whey ready-to-use supplementary foods (Plumpy’Sup; Nutriset)  Participants received approximately 75kcal/kg/d for 3 months. |
| **Outcomes** | 1. Recovery (weight-for-height z-score > -2)  2. Failure (No recovery)  3. Deterioration to severe acute malnutrition  4. Mortality  5. Weight Gain (g/kg/d)  6. Length gain (mm/d)  7. Mid-upper arm circumference Gain (mm/d)  8. Weight-for-height z-score End  9. Diarrhea (in first 2 weeks of treatment)  10. Vomiting (in first 2 weeks of treatment)  11. Default Rate (did not return for 3 consecutive visits)  12. Duration for recovery  13. Transferred to inpatient care |
| **Notes** | For purpose of analysis intervention groups two and three were combined under lipid-based nutrient supplements. |

#### Risk of bias table

| **Bias** | **Authors' judgement** | **Support for judgement** |
| --- | --- | --- |
| Random sequence generation (selection bias) |  | A block randomization list was created by using a computer random number generator. |
| Allocation concealment (selection bias) |  | Allocation was performed by caregivers drawing opaque envelopes containing one of nine coded letters corresponding to one of the three supplementary foods. This code was accessible only to the food distribution personnel, who did not assess participant outcomes or eligibility. |
| Blinding of participants and personnel (performance bias) |  | The investigators who performed the clinical assessments were blinded to the child’s assigned food group. The children and caregivers could not be blinded because the three supplementary foods differed in taste, appearance, and preparation required. |
| Blinding of outcome assessment (detection bias) |  | Investigator blinded trial |
| Incomplete outcome data (attrition bias) |  | Not significant. A total of 181 (6.7%) children missed a total of 198 visits. Only 1.3% of the children defaulted. |
| Selective reporting (reporting bias) |  | None detected |
| Other bias |  | None detected |
| Recruitment Bias |  |  |
| Loss of clusters |  |  |
| Baseline Imbalance |  |  |
| Incorrect Analysis |  |  |
| Comparison with individual randomized controlled trial |  |  |

### Matilsky 2009

| **Methods** | Randomized Controlled Trial |
| --- | --- |
| **Participants** | Twelve rural study sites were identified in the southern region of Malawi based on census reports of moderately wasted children provided by the World Food Program.  Inclusion Criteria:  1. Children with moderate wasting according to the WHO’s current standards (weight-for-height z-score < -2 but > -3) and with a good appetite  Exclusion Criteria  1. Children who had signs of severe malnutrition, including having a weight-for-height z-score< -3 and/or edema  2. Chronic illness, cardiac disease, congenital abnormalities, cancer  3. Children who had been discharged from the nutritional rehabilitation unit. |
| **Interventions** | Group 1: Milk Peanut Fortified Spread  Group 2: Soy Peanut Fortified Spread  Group 3: Corn Soy Blend  Supplement given for 8 weeks. |
| **Outcomes** | 1. Recovery (weight-for-length z-score >-2)  2. Deterioration to severe acute malnutrition  3. Mortality  4. Weight Gain  5. Height Gain  6. Mid-upper arm circumference Change  Children in all 3 groups received rations of 314 kJ/kg/d. The quantity of food provided was determined by the child’s body weight and was based on meeting ;50% of the estimated energy requirement of the moderately wasted child. |
| **Notes** | Data from Group 1 and 2 combined to avoid unit of analysis errors |

#### Risk of bias table

| **Bias** | **Authors' judgement** | **Support for judgement** |
| --- | --- | --- |
| Random sequence generation (selection bias) |  | A research assistant not involved in the study implemented the randomization process |
| Allocation concealment (selection bias) |  | Caretakers chose an envelope that contained one of six letters and this letter was recorded separately from the child’s clinical measurements. Each of the six letters corresponded to one of the three diets. |
| Blinding of participants and personnel (performance bias) |  | Field assistants who were aware of which letter corresponded to which food coordinated the food distribution process but did not assess the participants. Field workers and investigators remained unaware of the type of food each child received for the duration of the study. |
| Blinding of outcome assessment (detection bias) |  | Not mentioned |
| Incomplete outcome data (attrition bias) |  | Attrition low, 29/1362 |
| Selective reporting (reporting bias) |  | Not detected |
| Other bias |  | Not detected |
| Recruitment Bias |  |  |
| Loss of clusters |  |  |
| Baseline Imbalance |  |  |
| Incorrect Analysis |  |  |
| Comparison with individual randomized controlled trial |  |  |

### Medoua 2016

| **Methods** | Randomized Controlled Trial |
| --- | --- |
| **Participants** | Moderately malnourished children living in the health districts of Mvog-Beti and Evodoula in Cameroon.  Inclusion Criteria  1. Children aged 24-59 months  2. Weight-for-height z-score < - 2 and ≥ -3 without oedema  Exclusion Criteria  1. Poor appetite  2. Chronic debilitating illness  3. History of peanut allergy |
| **Interventions** | Group 1: Corn soy blend plus  Group 2: Ready-to-use supplementary foods  Every child received a daily ration of 167 kJ (40 kcal)/kg body weight during 56 d (approximately 50% of total energy requirement).  Deworming and nutritional counselling provided to all study participants |
| **Outcomes** | 1. Recovery from moderate acute malnutrition (defined as weight-for-height z-score > -2)  2. Time taken to recover |
| **Notes** |  |

#### Risk of bias table

| **Bias** | **Authors' judgement** | **Support for judgement** |
| --- | --- | --- |
| Random sequence generation (selection bias) |  | A randomization list was created using a random number generator (Stat Trek). |
| Allocation concealment (selection bias) |  | Allocation to either Corn soy blend plus or ready-to-use supplementary foods was performed by caregivers drawing from an opaque bag containing coded numbers corresponding to one of the two supplementary foods. The code was accessible only to the food distributor. |
| Blinding of participants and personnel (performance bias) |  | Investigators performing the clinical assessment and nutrition education were blinded to the child’s assigned food group. If two children were from the same household, both children were given the same type of food to reduce the likelihood of confounding study foods. |
| Blinding of outcome assessment (detection bias) |  | Not mentioned but unlikely given the adequate allocation concealment and blinding done. |
| Incomplete outcome data (attrition bias) |  | No loss to follow up |
| Selective reporting (reporting bias) |  | Not detected |
| Other bias |  | Not detected |
| Recruitment Bias |  |  |
| Loss of clusters |  |  |
| Baseline Imbalance |  |  |
| Incorrect Analysis |  |  |
| Comparison with individual randomized controlled trial |  |  |

### Nackers 2010

| **Methods** | Randomized Controlled Trial |
| --- | --- |
| **Participants** | The study was conducted in two Supplementary Feeding Centres in the remote and difficult-to-access villages of Mallawa and Bangaza (Magaria department, Zinder region, South of Niger). The area is affected by chronic food insecurity marked each year with a ‘hunger gap’, period when the previous year’s stocks have run out but the new crop is not yet ready for harvest. This period usually lasts from three to six months between May and October. In the Zinder region, in 2006, stunting among children < 5 years of age was estimated at 59% and wasting at 11%.  **Inclusion Criteria:**  1. weight-for-height of the reference median % from 70 to < 80% (National Center for Health Statistics reference), without oedema and with a mid-upper arm circumference > 110 mm.  2. All children measuring 65 to < 110 cm (used as a proxy for the age of 6 to 59 months)  **Exclusion Criteria**  1. Children requiring hospitalization as well as those who had been hospitalized or admitted in a nutritional programme in the previous two months  2. Children with a mid-upper arm circumference 135mm and apparently healthy |
| **Interventions** | Group 1: Ready-to-use therapeutic foods [Plumpy’Nut (Nutriset, Malaunay, France) two packs daily, i.e. 1000 kcal/ day]  Group 2: Corn soy blend pre-mix (1750 g of corn soy blend, 175 ml of vegetable oil and 105 g of sugar, i.e. 1231 kcal/ day)  In addition, in the corn soy blend pre-mix group only, children with a haemoglobin 9 and <11 g /dl were given a weekly iron supplementation (100–200 mg according to weight), as recommended by the nutritional national protocol.  Other interventions were similar in both groups and included at admission: measles vaccination, single vitamin A and folic acid supplementation, deworming and malaria testing (rapid diagnosis test: Paracheck) and treatment when positive. Nutritional advices, medical examination and appropriate treatment were provided on a weekly basis. A family ration of corn soy blend (2450 g), oil (140 ml) and sugar (140 g) in separate containers was given every week and a ration at discharge (50 kg of cereals, 7.5 kg of legume and 7.5 l of oil). |
| **Outcomes** | 1. Weight gain  2. Recovery rate (percent weight-for-height of the reference median 85% for 2 consecutive weeks)  3. Transfer to inpatient facility (Transfer criteria were medical (e.g. severe infectious diseases, severe anaemia and severe dehydration) and/or nutritional (development of oedema, weight loss with anorexia or deterioration of general condition).  4. Mortality  5. Non-responder (Children who did not reach the discharge criteria after 16 weeks)  6. Defaulter rates (Those who missed three consecutive visits)  7. Length of stay  8. Mid-upper arm circumference gain  9. Haemoglobin gain during treatment  10. Relapse  11. Height gain 6 months after discharge. |
| **Notes** |  |

#### Risk of bias table

| **Bias** | **Authors' judgement** | **Support for judgement** |
| --- | --- | --- |
| Random sequence generation (selection bias) |  | The allocation sequence (blocks of 10) was computer generated |
| Allocation concealment (selection bias) |  | Concealed in sealed envelopes |
| Blinding of participants and personnel (performance bias) |  | Cannot be done owing to the nature of intervention |
| Blinding of outcome assessment (detection bias) |  | Not mentioned; likely not done |
| Incomplete outcome data (attrition bias) |  | Attrition rate low 3/807 initially randomized. |
| Selective reporting (reporting bias) |  | Not detected |
| Other bias |  | During the course of the study, due to communication problems with those remote Supplementary Feeding Centres, children weighing > 8 kg at any time during their treatment were provided with three daily packs of ready-to-use therapeutic foods instead of two as planned in the study protocol. Consequently, these children were excluded from the analysis. |
| Recruitment Bias |  |  |
| Loss of clusters |  |  |
| Baseline Imbalance |  |  |
| Incorrect Analysis |  |  |
| Comparison with individual randomized controlled trial |  |  |

### Nikiema 2014 (C)

| **Methods** | Cluster randomized controlled trial |
| --- | --- |
| **Participants** | Children aged 6 to 24 months, with uncomplicated moderate acute malnutrition (weight-for-height z-score < -2 and > 3) and living in the catchment area of a health center were cumulatively included in the trial until the preset sample size was fulfilled. Children with a diagnosis of severe acute malnutrition were excluded from the trial. |
| **Interventions** | 1. Child Centred Counselling  2. Fortified Corn Soy Blend (corn soy blend plus plus) (65 g/d)  3. Ready-to-use supplementary foods (50 g/d) (Fortified Spread)  Both food supplements were designed to provide 250 kcal/d for 3 months.  All children, regardless of group allocation, received vitamin A (100,000 IU for children 6 to 12 mo of age, 200,000 IU for children .1 year of age) and 100 mg mebendazole (1 tablet 2 times per day for 3 days). Those with anemia (hemoglobin <11 g/dL) were given iron + folic acid syrup (100 mg; 1 dose 3 times per day) for 4 weeks. Vaccinations were also administered according to the national schedule. |
| **Outcomes** | 1. Recovery (defined as weight-for-height z-score > -2)  2. Failure to recover (weight-for-height z-score<-2 after 3 mo of treatment)  3. Defaulter  4. Deterioration to severe acute malnutrition  5. Mortality  6. Weight Gain  7. Length Gain  8. Mid-upper arm circumference Gain  9. Weight-for-height z-score  10. Height-for-age z-score |
| **Notes** |  |

#### Risk of bias table

| **Bias** | **Authors' judgement** | **Support for judgement** |
| --- | --- | --- |
| Random sequence generation (selection bias) |  | Random allocation was performed in public by the heads of each health center who were invited to draw 1 paper from a basket containing 18 pieces of paper (6 papers for each of the study arms). This was done under the supervision of the principal investigator during the launch meeting. |
| Allocation concealment (selection bias) |  | The method of randomization (see above) stated precludes allocation concealment. |
| Blinding of participants and personnel (performance bias) |  | Blinding not possible as one arm did not receive any dietary supplement. |
| Blinding of outcome assessment (detection bias) |  | Blinding not possible as one arm did not receive any dietary supplement. |
| Incomplete outcome data (attrition bias) |  | No significant attrition |
| Selective reporting (reporting bias) |  | None detected |
| Other bias |  | None detected |
| Recruitment Bias |  | Randmization of clusters done before the enrolment of study participants. |
| Loss of clusters |  | No loss of clusters |
| Baseline Imbalance |  | Cluster comparability not mentioned, at baseline nutritional status of mothers (BMI) and children (as measured by mid-upper arm circumference and height-for-age z-score, but not weight-for-height z-score) and child morbidity was slightly better in the ready-to-use supplementary foods arm |
| Incorrect Analysis |  | Not mentioned, author's clarification sought. |
| Comparison with individual randomized controlled trial |  |  |

### Vanelli 2014

| **Methods** | Non-randomized controlled trial |
| --- | --- |
| **Participants** | Children aged 6 to 60 months enrolled in the infirmary of “G” hospital  **Inclusion Criteria**  1. Weight-for height z-score of -3.0 to less than -2.0 SD  **Exclusion Criteria**  1. Acquired chronic disease . |
| **Interventions** | Group 1: Children fed with United Nations World Food Programme Supplementations regimen only  Group 2: Food Programme Supplementation regimen plus a supplement of “Parma pap” (ready-to-use therapeutic foods made by resident pediatricians)  All screened participants were receiving United Nations World Food Programme Supplementations made with corn flour, palm oil, dried fishes and milk powder. The ration for one child provided a maximum of 1000 to 1200 kcal/person/day and 10-12% of energy from protein. The ready-to-use therapeutic foods provided 200 Kcal/kg/day.  Supplements were provided for 12 weeks. |
| **Outcomes** | 1. Recovery (weight-for-height z-score value of -1.0 to less than -2.0)  2. Mortality  3. Failure  4. Weight-for-height z-score change |
| **Notes** |  |

#### Risk of bias table

| **Bias** | **Authors' judgement** | **Support for judgement** |
| --- | --- | --- |
| Random sequence generation (selection bias) |  | Not mentioned; the authors mention that the recruitment was 'personally managed' by the residents of the post-graduate School of Paediatrics, University of Parma, Italy, during their stay in hospital. |
| Allocation concealment (selection bias) |  | Not mentioned; not likely given the method of allocation |
| Blinding of participants and personnel (performance bias) |  | Not possible given the nature of intervention |
| Blinding of outcome assessment (detection bias) |  | Not done |
| Incomplete outcome data (attrition bias) |  | Drop-out rate was 14% (45/332) and it was higher in Group1 (35/45) than in Group 2 (10/45). |
| Selective reporting (reporting bias) |  | Not detected |
| Other bias |  | Not detected |
| Recruitment Bias |  |  |
| Loss of clusters |  |  |
| Baseline Imbalance |  |  |
| Incorrect Analysis |  |  |
| Comparison with individual randomized controlled trial |  |  |
