# Supplementary material for: Lipid based nutrient supplements (LNS) for treatment of children (6 months to 59 months) with moderate acute malnutrition (MAM): A systematic review
Source: PLoS One. 2017 Sep 21;12(9):e0182096. doi: 10.1371/journal.pone.0182096 (PMC5608196; doi:10.1371/journal.pone.0182096)
Supplement: S3 Table — (DOCX) [file pone.0182096.s004.docx]

**S3: PROGRESS Plus Equity checklist of included studies**

| **PROGRESS Plus Factors** | ***Ackatia-Armah 2015*** | ***Delchevalerie 2015*** | ***Karakochu 2012*** | ***LaGrone 2012*** | ***Matilsky 2009*** | ***Medoua 2015*** | **Nackers 2010** | ***Nikie`ma 2014*** | ***Vanelli 2014*** |
| --- | --- | --- | --- | --- | --- | --- | --- | --- | --- |
| Place | 12 community health centres (Centres de Santé Communautaire) and their surrounding communities in the Dioila Health District, which is located 170 km southeast of Bamako, the capital of Mali. | 5 Supplementary Feeding Centres based at public clinics in a rural setting of Bo district and run by Médecins Sans Frontières in Sierra Leone. Gondama center, where 18/19 defaults were from, was near a market. Defaulting was partly associated with living outside the catchment area. | 10 health centres and  health posts in the northern region of the Sidama zone, Ethiopia. This area was considered a “priority 2” level of food and nutrition insecurity by the country’s Emergency Nutrition Coordination Unit. All of the sites were chosen so  that all beneficiaries had equal access and were  within a walking distance of ≤5 km  for service users. | 18 rural therapeutic feeding clinics in southern Malawi. | 12 rural study sites in the southern region of Malawi. | Health districts of Mvog-Beti (urban area) or  Evodoula (rural area) in the Centre region of Cameroon | The study was conducted in two Supplementary  Feeding Centres  in the remote and difficult-to-access villages  of Mallawa and Bangaza in the Magaria department,  Zinder region, South of Niger. The supplementary feeding programmes in the region were supported by Médecins Sans Frontières. | 18 rural health centres in the  health district of Houndé, located in the Western region of  Burkina Faso. The district had relatively low food insecurity and an lipid-based nutrient supplements production unit was already in place. | Two study sites in Sierra Leone:  1. “G” hospital in a poor neighbourhood on the eastern end of Freetown.  2. Xaverian Mission in Makeni, a northern province. |
| Race/Ethnicity/Language | N/A | N/A | N/A | N/A | N/A | N/A | N/A | N/A | N/A. Language translators assisted the researchers in informing participant caretakers about the study in order to obtain informed consent. |
| Occupation | N/A for participants due to age. Of household heads (no details about who, exactly, was the household head): Most were farmers (81%) or unskilled labourers (9%). | N/A, although the researchers categorized the centres according to their location in either mining or agricultural areas. This suggests that many of the occupations of residents in the area, and of the caretakers of study participants, are likely related to mining and agriculture. | N/A | Participants came from families of subsistence farmers. | N/A | N/A | N/A | N/A for participants due to age, however most mothers were reportedly entirely dependent on their husbands to buy fish, meat, or oil, while some were also reportedly concerned with their agricultural and commercial activities. | N/A. |
| Gender/Sex | Approximately 52% of the study participants were female children, approximately 48% were male children. Caretakers that prepared and served the supplements were almost always the mothers of participants, suggesting traditional gender norms with regards to childcare. | 254 male children and 316 female child participants. | 697 female child participants and 428 male child participants. | 1684 of participants were female children and 1028 were male children. Data is not available regarding the sex or gender of the caretakers. | 493 male participants and 869 female child participants | 49 female child participants and 32 male child participants. | 252 female child participants and 299 male children. | 853 female children and 971 male children.  Most mothers were entirely dependent on their husbands to buy fish, meat, or oil to cook the recommended recipes, reflecting traditional gender norms in household makeup and labour division. Some mothers were concerned with their agricultural or commercial activities, which reflects some economic independence, but could also suggest financial necessity. | 155 male child participants and 181 female child participants. |
| Religion/Culture | N/A. | Food sharing discussed. | Food sharing reported. | Single annual harvest of maize reported. Suggests seasonal practice that may be incorporated into the culture, but this is not discussed. Food sharing was presumed. | A small number of caretakers reported sharing the supplements with others. Corn is the staple food. | Food sharing could not be excluded. | There is a ‘hunger gap’ each year that usually lasts from 3 to 6 months between May and October in this region when the previous year’s stocks have run out but the new crop is not yet ready for harvest. | N/A | Researchers were aware that peanuts and palm oil were the least expensive, most readily available and most popular ingredients to prepare a supplementary food with. This supplement was named ‘Pharma pap’ and was the subject of this study. Cultural beliefs did not allow researchers to suspend the Food Programme Supplementations regimen in one group of participants. |
| Education | N/A for participants due to age. Most (57%) of household heads had no education and most (85%) of the study participants’ mothers also had no education. However, signed consent was reportedly obtained from all of the participants’ caretakers.  Product-specific counseling on the preparation and serving  methods of the different dietary supplements was provided to the caretakers of participants. All anthropometrists were trained and standardized at the beginning of the study and on three more occasions during the study. | N/A. However, written informed consent was obtained from the caretakers (usually parents), suggesting some level of education. In the case of illiteracy, witnessed verbal consent was obtained. | N/A | Caretakers were all provided with nutrition and general health counselling. Study nurses gave the caregivers  information about the illness of the participants, the benefits  of supplementary feeding, feeding the supplement only to the enrolled child and not to share it, feeding the supplement in addition to the usual diet, how to store unfinished portions of the supplement, and spacing out the use of the daily portions to last until the next biweekly distribution. Additional instructions were given to caregivers of children in the corn soy blend plus plus arm about how to prepare the supplement properly; i.e. using a ratio of five parts water to one part dry flour. | Mothers were instructed that the supplements were medicines and that were not to be given to children who were not ill or shared with other children who were sick. Prompted by the healthcare professionals, the increased compliance and vigilance of caretakers (mostly mothers) contributed to high recovery rates. | It was inferred that most caretakers were literate since informed written consent was provided to the researchers and no mention is made of verbal consent. Nutrition and general health counselling were provided to all caregivers, including information about how best to use the food they had in their homes, the illness of the participants and the benefit of supplementary feeding. This investment in education is hypothesized to have contributed to high recovery rates despite the study providing the participants and their caretakers with lower quantities of supplements. Caregivers were instructed to continue to feed children their usual diet along with the supplementary food as medicine (364). The key messages included:  (i) continue to breast-feed your child until he is 2 years  old; (ii) wash your hands and your child’s hands with soap  and water before eating; (iii) wash your hands with soap  and water after using the toilet or cleaning your child’s  bottom; (iv) help your child eat and finish all food; (v) feed  your child a variety of foods every day; (vi) vegetables are  good for your child, it help him keep healthy and prevent  illness; (vii) foods from animals help your child gain  weight, grow strong and lively, give them every day;  (viii) give fruits to your child every day; (ix) add beans, soya or groundnuts in your child’s porridge every day; and (x) keep food and water covered. | Caretakers were provided with nutritional advice on a weekly basis. | Health workers in the CCC arm of the study were trained in communication and nutrition counselling by using a child-centred approach. A refresher training was organized at months 6 and 12 of the intervention. Formative supervision sessions were carried out  quarterly by the district nutrition officers to address identified  weaknesses in service delivery and questions raised by health  workers.  Most (82.6%) of study participants’ mothers had no education and 15.5% had achieved primary school education. Less than 2% of the mothers whose children participated in the study had high school and secondary education levels. The child caretakers in the CCC arm of the study also received weekly personalized child-centred counselling. Significantly lower attendance  was found for children in the CCC group, which suggests that the low recovery rate for CCC might be related to a greater defaulting rate and low attendance.  After each weekly consultation, caretakers were also invited to cooking sessions where recipes for optimizing child meals with local ingredients were shared. Each participant had an individual file in which all information regarding medical profile, advice received, strategy implementation notes and identified alternatives were recorded. Caretakers were instructed that food supplements were only intended for children with MAM. | N/A due to young age of participants and education of caretakers not mentioned. However, some mothers were literate as they signed informed consent forms. Mothers who were not literate gave verbal permission. In addition, mothers/caretakers of participants participated in the preparation of ‘pap’; inferring that they received preparation education, at least. |
| Socio-economic Status | Most of the participant’s households were low-income. In general, the children originated from agricultural households with low rates of literacy (of mothers, 85%), meagre possessions, poor housing quality (65% used a torch light for their primary source of light) and poor sanitary infrastructure (69% used a bucket/pan as their type of toilet facility). More than a third (40%) of the households were reportedly food insecure. Household food  Security information was obtained by using the standardized Household Food Insecurity Access Scale questionnaire. Food security status was determined to be unrelated to the participant’s growth outcomes and did not modify the growth responses to treatment.  Reported cost of the product in US$. | Low SES: Researchers ensured similar representations for the poorest areas in both study groups. Participants resided in a district where wasting and malnutrition were common as a result of underlying diseases (e.g. malaria, gastrointestinal and  respiratory infections), of poor access to clean water and appropriate weaning foods, and because of the seasonal peak of hunger from May to October corresponding to the rainy season. Study participants were also recruited while attending public health clinics that provide care free of charge to the population, or they were identified through outreach programs. | Participants resided in a region that was considered “priority 2” level of food and nutrition insecurity and where no other food assistance programs were implemented (912). Availability and cost of the treatment foods were not reported, although it is mentioned that ready-to-use supplementary foods are considerably more expensive per metric ton than corn soy blend, and the new World Food Programme recommendation, corn soy blend plus plus, is more expensive than the previous corn soy blend. | The participants came from families of subsistence farmers. Most (75%) households were moderately to severely food insecure, based on the Household Food Insecurity Access Scale. 50 mothers of participants were known to be dead and 84 fathers of participants had also passed away. 254 mothers and 84 of participants were known to be HIV positive. Because exclusionary criteria involved participation in another supplementary feeding program and if they had received therapy for acute malnutrition within 1 month prior to their presentation for participation consideration, nutrition related social networks and contacts are implied. Costs of the supplements (corn soy blend plus plus, Soy ready-to-use supplementary foods and Soy/whey ready-to-use supplementary foods are considered in US$. | N/A | Most children were living in contexts of moderate food insecurity. | Participants resided in an area that is affected by chronic food insecurity that is marked each year with a ‘hunger gap’, period when the previous year’s stocks have run out but the new crop is not yet ready for harvest. This period usually lasts from 3 to 6 months between May and October. | 78.6% of participants came from low (34.5%) and intermediate (44.1) socioeconomic levels. Most of the mothers or caretakers were illiterate (82.6%). | N/A. However, some caretakers found distances between villages and hospital “G”, lack of public transports, and the inability to entrust other children to another caretaker in the village during scheduled research visits at hospital prohibitive. This lack of resources resulted in missed evaluation days for participants. |
| Social capital | Community health centres in the Dioila Health District were partly chosen based on their history of collaboration with external projects. Participants were identified during 5 bimonthly community-based screening sessions, indicating social health networks and connections. These networks are supported by the existence of a national Community-based management of acute malnutrition protocol, which was based on international treatment guidelines, including the WHO guidelines. As a result, a supplement production and delivery network was in place. The study experienced logistical constraints related to availability of transportation and seasonally inaccessible roads.  Food sharing inferred through reports of home visit observations and data collection. All children identified as having severe acute  malnutrition (severe acute malnutrition; based on MOH criteria) with complication were immediately referred for treatment at the local district hospital. Fieldworkers visited the home in the event of missed scheduled clinic appointments to determine the reason for the absence and encourage continued participation. | Malnourished children were identified when attending sick to the clinic or by outreach programs. Data concerning family characteristics (e.g. living mother, deceased sibling, family size, and weekly expenses) was collected. Some of the participants were taken care of by someone other than their mothers, and this factor was partly associated with defaulting from the program. Discussions regarding distances to clinic infer a transportation network of some sort. Gondama center, where 18/19 defaults were from, was near the city market, which indicate a commercial network. | The Emergency Nutrition Coordination Unit in  Ethiopia uses a “priority” classification system to prioritize districts for nutrition services on the basis of food and nutrition security indicators and available resources. This priority classification system determines the extent to which health and nutrition services will be provided. All of the study supplementary feeding program sites were within a walking distance of 5 km for beneficiaries. | Other research trials being conducted at the same time indicate social health and academic networks. Caretakers and children who were aware they were infected with HIV had made use of health networks previously. Child participants were recruited when they presented at the clinic sites, again pointing to health network.  Information about family networks and connections was gathered, though information about HIV infection in both the participants and their mothers, whether the mothers and fathers were alive, and whether the mothers were the primary care providers. At least 11 of the participants were orphaned. | Children present at the study sites during recruitment period, suggesting there was at least one social network or connection. | Cameroon has a national protocol for the management of acute malnutrition, indicating political and health networks. No report about how children were recruited, so it is difficult to assess the social networks and connections of participants. Data was collected regarding  whether the participants lived with both of their parents, education  level of the mother, whether the mother was the  primary caregiver, whether the mother had a job, food consumption score, the sex of the child, whether the child lived in a rural or urban area, the type of supplementary food eating by the child and vaccination history of the child. However, only the type of supplementary food  received was a significant predictor of recovery | The two Supplementary Feeding Centres in Mallawa and Bangaza were part of a larger programme with a total of 14 Ambulatory Therapeutic Feeding Centres and 2 Inpatient  Therapeutic Feeding Centres, indicating a social network dedicated to health. | Children with moderate acute malnutrition were either detected passively via the  routine growth-monitoring program or at consultations for sick children, or actively through a monthly community-based  screening. This recruitment method suggests a developed social health network. Mothers received nonspecific  dietary advice through health services or community channels according to the national moderate acute malnutrition treatment recommendations, inferring community contacts and networks. Some mothers also demonstrated concern with agricultural or commercial activities that were not being attended to during study participation sessions.  The region had agricultural networks as a result of high cereal production. | N/A. However, social connections or networks were mentioned. For example, lack of public transportations and caretakers resulted in some study participants missing visits for evaluation on some days. Children in Freetown were also receiving FSPs, evidencing the existence of a social network. The research also infers international academic social networks and connections since most of the researchers were residents of the post-graduate School of Paediatrics from the University of Parma in Italy. The residents worked under the supervision and responsibility of a senior paediatrician working at the same hospital. |
| Plus   - Age - Disability - Sexual orientation | 1264 children ages 6 to 35 months of age. The restricted age range was used to limit the variability in expected growth responses and to focus on the younger children who might be more sensitive to different dietary regimens. Moreover, in Mali, the prevalence of wasting is greatest within the 6- to 35-mo age bracket.  Children with mild acute malnutrition were included in the trial.  Moderate acute malnutrition was defined as weight-for-length z-score between -3 and -2 or midupper arm circumference between 11.5 and 12.5 cm. A second set of inclusion criteria was based on national norms that were being used at the time the study took place.  Children with severe and chronic illnesses, including anaemia, malnutrition, congenital abnormalities, and HIV that might interfere with nutritional recovery, or a history of allergy to peanuts or previous serious allergic reactions to any substance and requiring emergency medical care, were excluded. | 570 children with moderate acute malnutrition aged 6 to 59 months. Weight-for-height of the reference median at 70-79% without edema. Children were excluded from the trial if one of the following conditions were observed: bilateral  edema, midupper arm circumference < 110 mm, weight-for-height of the reference median < 70%, or medical complications requiring hospitalization. Excluded children were transferred to a therapeutic feeding center or another medical facility for specialized care. | A total of 1125 children aged 6 to 60 months with moderate acute malnutrition. Moderate acute malnutrition is defined as a weight-for-height z-score between -2  and -3 or a weight-for-height percentile between 70%  and 79%, compared with a reference population.  Exclusion criteria  included the following: 1) children with midupper arm circumference, 110 mm, bilateral pitting edema, or other complications; 2) children  transferred from therapeutic feeding programs; and 3) children with any condition preventing safe ingestion of either food (i.e. peanut allergy). | 2712 children 6–59 months of age with moderate acute malnutrition, defined as a weight-for-height z-score between  -2 and -3. Children were excluded if they had a chronic debilitating illness (not including HIV or tuberculosis), or had a history of peanut allergy. Children were also excluded if they had received therapy  for acute malnutrition within 1 mo before presentation so as to focus the study primarily on the initial treatment of moderate acute malnutrition.  The results involving participants with HIV were similar across supplements. Children with HIV recovered less frequently than children who were not infected with HIV. Severe wasting was higher while the development of kwashiorkor was less frequent among participants who were HIV positive and who failed to recover.  19 of 24 participants who were receiving antiretroviral therapy recovered, whereas only 31 of 54 who were not receiving antiretroviral therapy. As the study points out, these results highlight the need to programmatically link HIV and malnutrition treatment programs in areas with a high prevalence of HIV. | 1362  Children 6-60 months of age and had moderate wasting according to WHO guidelines of weight-for-height z-score < -2 but ≥-3 were included in the study. Children who had signs of severe malnutrition, including having a weight-for-height z-score <-3 and/or edema, chronic illness, cardiac disease, congenital abnormalities, cancer, or those who had been discharged from the nutritional rehabilitation unit, were not eligible for the study. | 81 children aged 25-59 months with moderate acute malnutrition. Moderate acute malnutrition is defined as  weight-for-height between −3 SD and −2 SD below the median  weight-for-height of the WHO child growth standards  (weight-for-height z-score between -3 and -2) without oedema.Children were excluded from the study if they had no appetite, a chronic debilitating illness, and/or a history of peanut allergies. | 551 children 6-59 months of age and measuring 65 to <110cm that were newly admitted to either Supplementary Feeding Centres with moderate acute malnutrition and good appetite.  Moderate acute malnutrition was defined as a weight-for-height of the reference median percentage from 70 to < 80% (National Center for Health Statistics reference), without oedema and with a mid-upper arm circumference ≥ 110 mm.  Children requiring hospitalization and those who had been hospitalized or admitted in a nutritional programme in the previous 2 months were excluded from the study. | 1824 children aged 6-24 months of age and with uncomplicated moderate acute malnutrition (weight-for-height z-score < -2 and ≥ -3). Those with a diagnosis of severe acute malnutrition (presence of pitting  edema or weight-for-height z-score < -3, without complications) were excluded  from the trial. | 332 children 6-60 months of age in”G” hospital with moderate acute malnutrition. Participants from “G” hospital were all receiving United Nations World Food Programme Supplementations. 21 children with moderate acute malnutrition who were 8 to 21 months of age in Makeni. These children were not receiving supplements. Moderate acute malnutrition was defined as weight-for-height z-score of -3 to less than -2 SD. Children affected with an acquired chronic disease were excluded from the study. |
