# Supplementary material for: Lipid based nutrient supplements (LNS) for treatment of children (6 months to 59 months) with moderate acute malnutrition (MAM): A systematic review
Source: PLoS One. 2017 Sep 21;12(9):e0182096. doi: 10.1371/journal.pone.0182096 (PMC5608196; doi:10.1371/journal.pone.0182096)
Supplement: S4 Table — (DOCX) [file pone.0182096.s005.docx]

**S4 Account of PROGRESS-Plus factors reported in the included studies**

| **PROGRESS-Plus factors** | **Summary and analysis of reported factors** |
| --- | --- |
| **P**lace | All of the studies were conducted in healthcare centres that were located in low-income, rural areas of African countries. Medoua et al (48) and [Vanelli](Vanelli%202014) (45) also conducted a portion of their research in urban settings. Two of the studies (45, 46) were conducted in Sierra Leone and two others (22, 40) were conducted in Malawi. The other studies were conducted in Mali ([Ackatia-Armah](Ackatia-Armah%202015%20(C)) (35-38)), Ethiopia (47), Cameroon (48), Niger (49) and Burkina Faso (41). The studies took place in at least 79 study sites, including community health centres and posts, Supplementary Feeding Centres, therapeutic feeding clinics, a hospital and a mission post. Except for the study conducted in Burkina Faso, all participants resided in areas with moderate to high food insecurity. Defaulting in the studies by [Delchevalerie et al (46)](Delchevalerie%202015%20(C))  and [Nikiema](Nikiema%202014%20(C)) et al. (41) was related to distance from the participant’s home to the study follow-up sessions. Transportation was not readily available and some roads were seasonably inaccessible for study participants and their caretakers in [Ackatia-Armah](Ackatia-Armah%202015%20(C)) et al’s (35-38) research. Nevertheless, most participants’ caregivers voluntarily attended the scheduled follow-up visits. [Karakochuk](Karakochuk%202012%20(C)) (47) partly attributed the relatively low defaulting rates to the accessibility of the program. However, the program took place in an area where there was no other food supplementation program in place, which could have contributed to the low defaulting rate. [Vanelli](Vanelli%202014)’s study (45) was especially designed to be adapted to the place in which lipid-based supplements implementation took place and made use of locally available and popular ingredients to produce the research supplement. |
| **R**ace/Ethnicity/Language | Race and ethnicity were not taken into account by any of the studies included in this review. This exclusion suggests all participants were considered racially the same and/or that racial differences were considered insignificant to the studies’ purposes and results. However, the exclusion is significant as all of the studies took place in African countries and regions that are ethnically diverse, and which have been historically affected by racial and ethnic ideologies and tensions. Without information regarding the racial or ethnic groups that the study participants and their caretakers belonged to it is difficult to assess whether and to what extent racial or ethnic factors affect nutritional status in the study locations. Language differences among populations in a particular region could also affect access to and comprehension of information or educational opportunities that inform practices which later affect health. This includes information and educational supports regarding the preparation, provision and adherence to supplements that were provided as part of the studies. Language differences and factors were not reported in the studies except in one case when translators were required in order to obtain informed consent from the participants’ caretakers (45). |
| **O**ccupation | This factor was one of the least reported among the included studies. Due to the study participants' age, their occupation was not factored in the selected articles. However, some of the studies reported about the occupations of the participant’s caretakers. Two of the studies ([Ackatia-Armah](Ackatia-Armah%202015%20(C)) et al (35-38) and [LaGrone et al (39-40)](LaGrone%202012) ) indicated that most of the caretakers (usually the parents) of participants were farmers. [Nikiema](Nikiema%202014%20(C)) (41) reported that most mothers were entirely dependent on their husbands to purchase food items, indicating traditional gendered divisions of labour where men work for wages, typically outside of the home, while women work within the home caring for children and do not receive wages. Other mothers whose children participated in [Nikiema](Nikiema%202014%20(C))’s (41) study expressed concern related with their agricultural and commercial activities, suggesting that these caretakers were possibly farmers and/or merchants. The occupation of caretakers in the other studies is unknown. None of the studies considered the relationship between the occupations of caretakers, the time these caretakers spent on their occupations, the time spent supporting the participant’s recovery with supplements and the recovery rates of participants. This sort of discussion may have provided insights regarding the intervention design, types of knowledge or capacity supports that caretakers in similar occupations and with similar time limitations may require to optimize the recovery rates of children with moderate acute malnutrition through the use of lipid-based nutrient supplements. |
| **G**ender/Sex | All of the included articles reported this category and provided the distribution by sex of the participants. Female child participants outnumbered male children in seven of the nine studies, while [Nackers](Nackers%202010) et al. (49) and [Nikiema et al.](Nikiema%202014%20(C))(41) included more male child participants than female children. [Karakochuk](Karakochuk%202012%20(C)) et al. (47) reported that nonresponse rates in their study were not related to the sex, age, initial weight or health status of the participants. None of the other studies provided discussions of the results and recovery rates distributed by sex, nor did they provide detailed reports about the distribution by sex of the participants’ caretakers. This exclusion suggests that there were few, if any, observable differences in treatment or recovery rates distributed by the sex of either the participants or their caretakers. However, the exclusion is significant as female children often experience less access to resources, including food and nutrition, than male children as a result of discriminatory beliefs and gender norms. Women are also often the primary caretakers of children as a result of gender norms. This was true in [Ackatia-Armah](Ackatia-Armah%202015%20(C)) et al. (35-38), [Nikiema](Nikiema%202014%20(C)) et al. (41), and Vanelli’s (45) studies, which reported that most caretakers of the participants were their mothers. None of the studies reported that fathers accompanied the participants to healthcare centres or follow-up sessions, nor did they mention fathers’ involvement in the daily care of infants. [Ackatia-Armah](Ackatia-Armah%202015%20(C)) et al. (35-38) gathered information regarding the educational level and occupations of ‘heads of household’, but it was mothers who often prepared and served the supplements to participants, even when they were also the heads of household. Mothers in [Vanelli et al. (45)](Vanelli%202014)’s research were involved in the preparation of the ready-to-use therapeutic food that was used in the study. Most of the mothers in [Nikiema](Nikiema%202014%20(C))’s study (41) were entirely dependent on their husbands to purchase food items, indicating traditional gendered divisions of labour where men typically work outside of the home for wages while women typically do not receive wages working within the home and caring for children. Furthermore, some of the caretakers involved in this research were concerned that participating in the study affected their agricultural or economic activities, which suggests some economic and labour independence for these women and mothers, but could also indicate the socioeconomic necessity of their households. Studies that include information about whether or not parents are deceased do not include reports of the sex/gender of caretakers that are not parents. This information could have indicated if communication nuances or incentives were required that addressed and appealed to caretakers who were not the parents of study participants. This is particularly important when defaulting from a supplementary program is common among participants who are cared for by people who are not their parents, or their mothers in particular (40). |
| **R**eligion/ Culture | This category was one of the most underreported among the included studies. Religious beliefs were not mentioned and information about cultural factors had to be extrapolated from the general discussions. Except for the studies by [Ackatia-Armah](Ackatia-Armah%202015%20(C)) et al. (35-38) and [Nikiema](Nikiema%202014%20(C)) et al. (41), all of the studies mentioned food sharing in the regions where the research took place. [LaGrone (39, 40)](LaGrone%202012)  and [Matilsky](Matilsky%202009) et al. (22) mention that maize is the staple food crop in the areas where their studies were conducted and [LaGrone](LaGrone%202012) indicates that a single annual harvest takes place. No further mention about how food sharing, maize or single harvests are incorporated within the local customs or culture. The research by [Vanelli et al. (45)](Vanelli%202014) contributed the most to understanding cultural factors to consider when implementing lipid-based nutrient supplement programmes. They reported that cultural beliefs did not allow the researchers to suspend the Food Programme Supplementations regimen in the “G” hospital arm of their study; however, no further details about whose beliefs they are referring to -- whether the participants or researchers’ -- are provided. Their study concluded that ready-to-use therapeutic foods can be prepared with locally-available ingredients that are popular among and familiar to caretakers (i.e. culturally acceptable), provided that caretakers are provided with access to the materials and capacity supports for the preparation of the product. Local preparation of a lipid-based nutrient supplement would help close the equity gap that exists between those who can afford ready-to-use therapeutic foods and those who are most at need of them – usually resource scarce and low-income households. The approach by [Vanelli](Vanelli%202014)’s team of researchers promotes the active participation of caretakers and the community in owning the supplement produced with local ingredients. During the three years that this study took place the researchers did not observe seasonal interferences on the preparation, administration and acceptability of the supplement. |
| **E**ducation | All of the trial participants were too young for their education to be taken into account; however, most studies included some information regarding this category and the caretakers of participants, with various degrees of detail. Most mothers and other primary caretakers did not have formal education. When information about the education levels of husbands, heads of household or partners was collected, husbands/partners usually had higher levels of educational achievement than mothers. Despite high levels of illiteracy across the included studies, most of the studies collected written informed consents. [Delchevalerie](Delchevalerie%202015%20(C)) et al. (46) and [Vanelli](Vanelli%202014) et al. (45) obtained witnessed verbal consent when caretakers were illiterate. The caretakers in the studies by [LaGrone](LaGrone%202012) (39, 40), [Matilsky et al.](Matilsky%202009) (22), [Medoua](Medoua%202016) et al. (48), [Nackers](Nackers%202010) et al. (49), [Nikiema et al. (41)](Nikiema%202014%20(C)) and [Vanelli](Vanelli%202014) et al. (45) received capacity supports during the trials. Most included instructions not to share the supplements with other family members or to not provide the supplements to children who are not sick. [Vanelli](Vanelli%202014) et al. (45) trained their caretakers to prepare the lipid-based nutrient supplement from mostly locally available products. General nutrition and health counselling was provided by [LaGrone et al (39, 40)](LaGrone%202012), [Matilsky](Matilsky%202009) et al. (22), [Medoua](Medoua%202016) et al. (48) and [Nikiema](Nikiema%202014%20(C)) et al. (41), in addition to supplement preparation instructions. Both [Matilsky](Matilsky%202009) et al. (22) and [Medoua](Medoua%202016) et al. (48) attributed part of the success of their studies to the investments of educating caretakers. Only [Nikiema](Nikiema%202014%20(C)) et al. (41) experienced high defaulter rates among caretakers and participants who were receiving educational supports. While this occurrance was partly attributed to the distance between the clinic and residences of participants, the training programme itself could have been inadequate. For example, [Nikiema](Nikiema%202014%20(C)) et al. (41) reports that an individual file was made for all study participants that documented their medical profile, the advice their caretakers had received, strategy implementation notes and successful alternatives. However, it is not entirely clear who these files were primarily intended for – the researchers or the caretakers. Taking into account the high levels of illiteracy reported by the study, if the files were intended for the caretakers it is questionable that these would have been adequate resources for them. |
| **S**ocio- economic Status | The socioeconomic status of research participants were reported by all but one of the included studies (22). With the exception of Nikiema’s study (41), most participants and their caretakers came from low income and food insecure households. [Ackatia-Armah](Ackatia-Armah%202015%20(C)) et al. (35-38) also reported on household possessions, housing quality, light sources, and the sanitary infrastructure of their study participants and found that most lacked adequate means. Similarities in the socioeconomic status of the participants in the study reinforce the need to link nutrition actions with strategies that address sanitary and housing conditions, practices and qualities. In addition, they reinforce the fact that the social determinants of health are interconnected and, therefore, interdependent on multisectoral interventions to alleviate conditions that produce and reproduce health inequities. |
| **S**ocial Capital | Social capital was not reported or discussed by any of the included studies. As a result, social networks or connections had to be deduced from the information provided in the articles. The most obvious social connections or networks were healthcare related. In fact, most of the studies’ participants were recruited at healthcare facilities and supplementary feeding programs. Many children were excluded from participating in the studies if they had previously received healthcare for moderate acute malnutrition. Likewise, studies included or excluded participants who were known to be infected with HIV. The studies by [Ackatia-Armah](Ackatia-Armah%202015%20(C)) et al. (35-38), [Karakochuk](Karakochuk%202012%20(C)) (47), [Medoua](Medoua%202016) (48), and [Nikiema](Nikiema%202014%20(C)) (41) report national protocols and systems in place that are designed to respond to food insecurity and malnutrition. This is a strong indication that across the studies participants had links to political and social networks and connections related to health. Information about family networks were also gathered in the studies by [Ackatia-Armah](Ackatia-Armah%202015%20(C)) (35-38), [Delchevalerie](Delchevalerie%202015%20(C)) (46), [LaGrone](LaGrone%202012) (39, 40) and [Medoua](Medoua%202016) (48). Extended family care networks were most obvious in [LaGrone](LaGrone%202012) and [Medoua](Medoua%202016)’s studies (39, 40, 48) as several children’s parents were deceased and, therefore, the children were being taken care of by someone else. In [Delchevalerie et al.](Delchevalerie%202015%20(C))’s study (46), the status of a participant being taken care of by someone other than their mother was partly associated with defaulting from the program. Kin and community networks are also suggested in [Vanelli](Vanelli%202014) et al. (45), which reported on caregivers’ concerns with finding appropriate supervision for other children left at home while they participated in the study. Without further information it is challenging to determine how social capital plays a role in the success of the included studies. |
| **Plus** · Age · Disability · Sexual Orientation | All of the studies’ participants were between 6 to 60 months of age. They also all had moderate acute malnutrition, which was mostly defined according to WHO standards of weight-for-height z-score of -3.0 to less than -2.0 SD, and corresponding to a body weight-for-height of over 70% but less than 80% of the standard reference values. [LaGrone](LaGrone%202012) et al. (39, 40) included children with HIV while most of the other studies excluded children with the infection. Most studies reported referring children whose condition upon recruitment was, or later deteriorated into, severe acute malnutrition to local hospitals and clinics. Due to the age of the children, their sexual orientation was not reported nor a factor that determined their health. None of the studies reported on the sexual orientation of caretakers either. |
