# Supplementary material for: Lipid based nutrient supplements (LNS) for treatment of children (6 months to 59 months) with moderate acute malnutrition (MAM): A systematic review
Source: PLoS One. 2017 Sep 21;12(9):e0182096. doi: 10.1371/journal.pone.0182096 (PMC5608196; doi:10.1371/journal.pone.0182096)
Supplement: S5 Table — (DOCX) [file pone.0182096.s006.docx]

## S5: Characteristics of excluded studies

### Ciliberto 2005

| **Reason for exclusion** | Includes children with severe acute malnutrition after stabilization phase.  Children aged 10 to 60 months attending 1 of 7 Nutrition Rehabilitation Units for Severe Acute Malnutrition from December 2002 to June 2003 were screened for eligibility for the second phase of treatment for childhood malnutrition where lipid-based nutrient supplements was tested as an alternative to F100. These children had received the first phase, that included dietary therapy with a milk-based liquid food (F-75). |
| --- | --- |

### Defourny 2007

| **Reason for exclusion** | Prospective observational study, no control group  This study measures the response of almost 57000 malnourished children attending the Médecins Sans Frontières therapeutic feeding programme in Maradi, Niger in terms of cure, mortality and weight gain. There was no control group. |
| --- | --- |

### Dube 2009

| **Reason for exclusion** | Outcome not of interest, studies acceptability and energy intake  This is an acceptability trial with cross-over design. Children were offered weighed amounts of ready-to-use therapeutic foods and khichri in unlimited amounts for 2 days, one meal of each on both days. Acceptability of ready-to-use therapeutic foods compared to khichri based on direct observation and energy intake for test and control meals was assessed. |
| --- | --- |

### Fabiansen 2016

| **Reason for exclusion** | Observational study, no control group  This was an observational study nested in a randomized trial that investigated the effectiveness of new formulations of corn-soy blend and lipid-based nutrient supplements. The study assessed the difference in growth rate between children < 67 cm and those ≥ 67 cm in length at program admission. |
| --- | --- |

### Iuel-Brockdorf 2016

| **Reason for exclusion** | Outcome not of interest, studies acceptability  The objective of this study was to evaluate, within the context of a randomized controlled trial of product effectiveness, the acceptability of new formulations of six corn-soy blended flours (corn soy blend) and six lipid based nutrient supplements (lipid-based nutrient supplements) with different quantities of milk and qualities of soy for the treatment of children with moderate acute malnutrition. |
| --- | --- |

### Kuusipalo 2006

| **Reason for exclusion** | Study participants did not have moderate acute malnutrition as defined by weight-for-height z-score; included study participants had weight-for-age z-score less than -2  Infants 6 to 17 months of age were eligible to participate in the study if they were underweight, as defined by a weight that was below the green area when plotted on the Malawian road to health card (corresponding approximately to a weight-for-age z-score less than j2 of the World Health Organisation (WHO)-adopted National Center for Health Statistics reference curve). |
| --- | --- |

### Lagrone 2010

| **Reason for exclusion** | Prospective observational study, no control group  Children aged 6 to 59 months were recruited in rural southern Malawi. Each child received 65 kcal/kg/d of locally produced soy/peanut ready-to-use supplementary foods for 8 weeks. There was no control group. |
| --- | --- |

### Lin 2008

| **Reason for exclusion** | Study participants not moderately malnourished  All children 5.5 to 6.5 months of age residing in 8 villages in the Machinga district of southern Malawi without evidence of edema or severe chronic illness were eligible for the study. |
| --- | --- |

### Maleta 2004

| **Reason for exclusion** | Children did not have moderate acute malnutrition as defined; study participants were underweight (weight-for-age z-score < -2) and stunted (height-for-age z-score < -2).  Sixty-one underweight, stunted children 42 to 60 months of age were recruited in rural Malawi, in southeastern Africa. They received either ready-to-use therapeutic foods or maize and soy flour for 12 weeks. The inclusion criteria for the trial was underweight (weight-for-age z-score < -2) and stunting (height-for-age z-score < -2). The weight-for-height z-score ranged between -0.3 to -3. |
| --- | --- |

### Patel 2005

| **Reason for exclusion** | Children did not have moderate acute malnutrition, weight-for-height <85%, but >80%  Children, aged 10 to --60 months at risk of malnutrition, weight-for-height > 80%, but < 85% of the standard reference values of WHO) without oedema, were eligible for the study. These anthropometric criteria defining children at risk of malnutrition were chosen as these are specified by the Malawian national guidelines. |
| --- | --- |

### Phuka 2009

| **Reason for exclusion** | Children did not have moderate acute malnutrition as defined; included study participants had weight-for-age z-score < -2  Inclusion criteria for the trial included age of at least 6 months but less than 15 months, low weight-for-age z-score ( < -2.0), assumed residence in the study area throughout the follow-up period and signed informed consent from at least one authorized guardian. Exclusion criteria were severe wasting, weight-for-length z-score (< -3.0). |
| --- | --- |

### Purwestri 2012

| **Reason for exclusion** | Study participants not moderately malnourished; with weight-for-height z-score ≥ -2 to < -1.5 SD  This study reports on a comparison of supplementary feeding program outcomes of mildly wasted children with weight-for-height z-score ≥-2 to <-1.5 SD aged ≥6 to <60 months old given locally produced Ready-to-Use-Food-Nias biscuits. |
| --- | --- |

### Roy 2005

| **Reason for exclusion** | Lipid-based nutrient supplements not used as intervention  Supplementary feeding group received intensive nutrition education twice a week and supplementary feeding (8 to 9 g protein and 300 kcal per day from 40 g roasted and powdered rice, 20 g roasted and powdered pulse, 10 g molasses, and 6 g oil) for six days a week. |
| --- | --- |

### Scherbaum 2015

| **Reason for exclusion** | Both groups received lipid-based nutrient supplements, no control group; study participants mildly to moderately wasted.  This study compared the effectiveness of two nutritionally comparable forms of locally produced ready-to-use foods for daily feeding programmes of moderately to mildly wasted children (weight-for-height z-score≥-3 to <-1.5SD) were assessed on Nias, Indonesia.Both dietary treatments were locally prepared in the study area and consisted of comparable macro- and micronutrients. |
| --- | --- |

### Singh 2010

| **Reason for exclusion** | Children did not have moderate acute malnutrition as defined; included study participants had weight-for-age z-score <-2  Children aged 18-60 months, -2 SD weight-for-age and below but not requiring hospitalization for malnutrition, were considered eligible. |
| --- | --- |

### Thakwalakwa 2010

| **Reason for exclusion** | Children did not have moderate acute malnutrition as defined; included study participants had weight-for-age z-score <-2  The target population for enrollment included moderately underweight infants and children who met the following inclusion criteria: a signed, informed consent from at least 1 guardian, aged between 6 and 15 mo, weight-for-age z-score < -2 based on the National Centre for Health Statistics/ Centers for Disease Control and Prevention growth reference. |
| --- | --- |

### Thakwalakwa 2012

| **Reason for exclusion** | Children did not have moderate acute malnutrition as defined; included study participants had weight-for-age z-score < -2  The inclusion criteria were: a signed informed consent from at least one guardian, aged between 6 and 15 months, weight-for-age z-score < -2, availability during the period of the study and permanent residence in the catchment area. |
| --- | --- |

### Tomedi 2012

| **Reason for exclusion** | Study participants not malnourished, with weight-for-length z-score greater than -2 at baseline  All children who lived in the two target sub-locations were eligible to participate if they were 6 to 20 months of age at the onset of the study and had weight-for-height z-score> -2. |
| --- | --- |

### Traore 2013

| **Reason for exclusion** | Lipid-based nutrient supplements not used in the intervention arms.  Children aged 6 to 24 months, affected by moderate acute malnutrition (-3 < weight-for-length z-score < -2) were randomly assigned to 3 groups receiving gruels prepared from one of the three following locally processed fortified flours: the two first (Energy density = 150 kcal/100g) were formulated according to specific recommendations for moderate acute malnutrition, one with 8% dried milk (MAM-FFM), the second without milk (MAM-FF), and the third (ED = 100 kcal/100g) was the Misola (MIS) flour formulated according to recommendations for healthy children. |
| --- | --- |
