# Supplementary material for: Lipid based nutrient supplements (LNS) for treatment of children (6 months to 59 months) with moderate acute malnutrition (MAM): A systematic review
Source: PLoS One. 2017 Sep 21;12(9):e0182096. doi: 10.1371/journal.pone.0182096 (PMC5608196; doi:10.1371/journal.pone.0182096)
Supplement: S6 Table — (DOCX) [file pone.0182096.s007.docx]

**S6 Characteristics of studies awaiting classification**

### Manary 2014

| **Methods** | Cluster randomized controlled trial |
| --- | --- |
| **Participants** | 6 Months to 59 Months  Inclusion Criteria   - Child diagnosed with moderate acute malnutrition and enrolled to receive a ration from a feeding site (i.e. enrolled in the supplementary feeding program)   Exclusion Criteria   - Children with bipedal oedema - Children receiving food rations from another organization (e.g. USAID or UNICEF) |
| **Interventions** | 1. Super Cereal Plus (SC+) at 800 kcal/d, 215 g/d (Control group) 2. Super Cereal (SC) and oil and sugar at 998 kcal/d - 200 g SC and 20 g oil and 20 g sugar, per day 3. Corn soy Blend 14 (corn soy blend14) and oil at 978 kcal/day - 150 g corn soy blend14 and 45 g oil, per day 4. Plumpy'Sup - 500 kcal/d, 92 g/d |
| **Outcomes** | Primary  1. Recovery from moderate acute malnutrition [ Time Frame: 12 weeks ] (mid-upper arm circumference ≥ 12.5 cm by 12 weeks once)  2. Percent default/non-response: children who do not recover after 12 weeks  3. Percent relapse: children who developed moderate acute malnutrition again within 6 months of recovery  Secondary   - Change in Growth rates - Duration of treatment - Cost estimates for participation - Default reason - Change in recovery status after 12 weeks |
| **Notes** | The authors were contacted for details regarding this study. We were informed that the study is under the publication process. |

### van der Kam 2016

| **Methods** | Randomized controlled trial |
| --- | --- |
| **Participants** | Children aged 6-59 months diagnosed as having malaria, diarrhea or respiratory tract infection at an outpatient clinic in Goronyo, Nigeria |
| **Interventions** | The study participants were randomised to one of three arms: one sachet/d of ready-to-use therapeutic foods; two sachets per day of micronutrients or no supplement (control) for 14 days for each illness over 6 months. |
| **Outcomes** | 1. Negative Nutritional Outcome  2. Mortality |
| **Notes** | The published data pertains to all included children. The authors have been requested to provide segregated data regarding children with moderate acute malnutrition. |
