# Supplementary material for: Lipid based nutrient supplements (LNS) for treatment of children (6 months to 59 months) with moderate acute malnutrition (MAM): A systematic review
Source: PLoS One. 2017 Sep 21;12(9):e0182096. doi: 10.1371/journal.pone.0182096 (PMC5608196; doi:10.1371/journal.pone.0182096)
Supplement: S7 Table — (DOCX) [file pone.0182096.s008.docx]

## S7 Characteristics of ongoing studies

### Friis 2013

| **Study name** | Effectiveness of improved diets for children with moderate acute malnutrition: a randomized controlled trial in Province du Passoré, Burkina Faso |
| --- | --- |
| **Methods** | Randomised nutrition intervention trial with a 2-by-2-by-3 factorial design |
| **Participants** | Inclusion criteria:  1. Weight-for-height z-score = -3 and < -2 (based on the WHO growth standard) or Mid Upper Arm Circumference =115mm and < 125 mm 2. Six to 23 months of age 3. Resident in the catchment area at the time of inclusion 4. Whose parents/guardians have signed (or thumb-printed whenever illiterate) the informed consent  Exclusion criteria:  1. Children with severe acute malnutrition defined as a weight-for-height z-score < -3, and/or a mid-upper arm circumference <115 mm, and/or bilateral pitting oedema. 2. Children with medical complications requiring hospitalization 3. Children whose household plans to leave the catchment area in the next 6 months 4. Children with a hemoglobin concentration <4g/dl or with evidence of a decompensate anemia (e.g. dyspnea, tachycardia etc.) 5. Children who have been treated for severe acute malnutrition or who have been hospitalized in the last 2 months 6. Children with known allergy to milk, peanut, corn soya blend (corn soy blend) and/or ready-to-use therapeutic food 7. Children with a severe disability limiting the possibility of investigations 8. Children enrolled in any other nutritional program or part of any other study conducted in the area |
| **Interventions** | Children will receive one of 12 newly developed food products at a quantity of 500kcal per day for 12 weeks. The 12 products include 6 improved corn soy blends and 6 lipid based nutrient supplements |
| **Outcomes** | Primary  1. Absolute lean mass increment from baseline to 3 months  Secondary  1. Increase of weight-for-height z-score to > -2 and linear growth 2. Increase in mid upper arm circumference > 125 mm 3. Physical activity measured using an accelerometer, at 0, 3 and 6 months 4. Motor and language milestones development 5. Morbidity 6. Level of biological indicators of nutritional status, growth and immune function (haemoglobin, serum acute phase proteins, serum ferritin, essential fatty acids, insulin-like growth factor-1) at 0, 3 and 6 months 7. Thymus size using an ultrasound scanner at 0, 3 and 6 months 8. Acceptability of and adherence of products |
| **Starting date** | 02/08/2013 |
| **Contact information** | hfr@life.ku.dk |
| **Notes** | Recruitment completed |
