# Supplementary material for: Lipid based nutrient supplements (LNS) for treatment of children (6 months to 59 months) with moderate acute malnutrition (MAM): A systematic review
Source: PLoS One. 2017 Sep 21;12(9):e0182096. doi: 10.1371/journal.pone.0182096 (PMC5608196; doi:10.1371/journal.pone.0182096)
Supplement: S8 Table — (DOCX) [file pone.0182096.s009.docx]

**S8 Lipid-based nutrient supplement versus Specially formulated micronutrient fortified foods**

| **Outcome or Subgroup** | **Studies** | **Participants** | **Statistical Method** | **Effect Estimate** |
| --- | --- | --- | --- | --- |

| 1.1 Recovery from moderate acute malnutrition (ALL) | 9 |  | Risk Ratio (M-H, Random, 95% CI) | Subtotals only |
| --- | --- | --- | --- | --- |
| 1.1.1 RCT | 8 | 8934 | Risk Ratio (M-H, Random, 95% CI) | 1.08 [1.02, 1.14] |
| 1.1.2 Non-RCT | 1 | 336 | Risk Ratio (M-H, Random, 95% CI) | 1.23 [1.06, 1.42] |
| 1.2 Recovery from moderate acute malnutrition (SUBGROUP: by age) | 8 | 8934 | Risk Ratio (M-H, Random, 95% CI) | 1.08 [1.02, 1.14] |
| 1.2.2 6-23 months of age | 1 | 1369 | Risk Ratio (M-H, Random, 95% CI) | 1.00 [0.94, 1.06] |
| 1.2.3 24-59 months of age | 1 | 81 | Risk Ratio (M-H, Random, 95% CI) | 1.16 [0.93, 1.46] |
| 1.2.4 Mixed | 6 | 7484 | Risk Ratio (M-H, Random, 95% CI) | 1.09 [1.02, 1.16] |

| 1.3 Recovery from moderate acute malnutrition (SUBGROUP: by type of supplement) | 8 | 8934 | Risk Ratio (M-H, Random, 95% CI) | 1.08 [1.02, 1.14] |
| --- | --- | --- | --- | --- |
| 1.3.2 RUSF | 7 | 8483 | Risk Ratio (M-H, Random, 95% CI) | 1.06 [1.01, 1.11] |
| 1.3.3 RUTF | 1 | 451 | Risk Ratio (M-H, Random, 95% CI) | 1.23 [1.09, 1.38] |
| 1.4 Recovery from moderate acute malnutrition (SUBGROUP: by duration of supplementation) | 8 | 8934 | Risk Ratio (M-H, Random, 95% CI) | 1.08 [1.02, 1.14] |
| 1.4.1 8 weeks or less | 2 | 1443 | Risk Ratio (M-H, Random, 95% CI) | 1.11 [1.04, 1.18] |
| 1.4.2 9-12 weeks | 3 | 5345 | Risk Ratio (M-H, Random, 95% CI) | 1.06 [0.97, 1.15] |
| 1.4.3 13 weeks or longer | 3 | 2146 | Risk Ratio (M-H, Random, 95% CI) | 1.09 [0.94, 1.27] |

| 1.5 Recovery from moderate acute malnutrition (ALL) | 9 | 9270 | Risk Ratio (M-H, Random, 95% CI) | 1.09 [1.03, 1.15] |
| --- | --- | --- | --- | --- |
| 1.5.1 Cluster RCT | 4 | 4328 | Risk Ratio (M-H, Random, 95% CI) | 1.06 [0.97, 1.16] |
| 1.5.2 RCT | 4 | 4606 | Risk Ratio (M-H, Random, 95% CI) | 1.11 [1.01, 1.21] |
| 1.5.3 Non-RCT | 1 | 336 | Risk Ratio (M-H, Random, 95% CI) | 1.23 [1.06, 1.42] |
| 1.6 Recovery from moderate acute malnutrition (SUBGROUP: Calories provided) | 8 | 8934 | Risk Ratio (M-H, Random, 95% CI) | 1.08 [1.02, 1.14] |
| 1.6.1 250 or less | 1 | 1369 | Risk Ratio (M-H, Random, 95% CI) | 1.00 [0.94, 1.06] |
| 1.6.2 250-500 | 5 | 6544 | Risk Ratio (M-H, Random, 95% CI) | 1.10 [1.03, 1.18] |
| 1.6.3 1000 or more | 2 | 1021 | Risk Ratio (M-H, Random, 95% CI) | 1.09 [0.84, 1.43] |

| 1.7 Recovery from moderate acute malnutrition (SUBGROUP: Milk based LNS versus Non milk based LNS) | 8 | 8935 | Risk Ratio (M-H, Random, 95% CI) | 1.08 [1.02, 1.13] |
| --- | --- | --- | --- | --- |
| 1.7.1 Milk Based LNS | 2 | 1140 | Risk Ratio (M-H, Random, 95% CI) | 1.15 [1.03, 1.29] |
| 1.7.2 Non Milk Based LNS | 7 | 7795 | Risk Ratio (M-H, Random, 95% CI) | 1.06 [1.01, 1.11] |
| 1.8 No recovery | 8 |  | Risk Ratio (M-H, Random, 95% CI) | Subtotals only |
| 1.8.1 Cluster RCT and RCT | 7 | 8364 | Risk Ratio (M-H, Random, 95% CI) | 0.70 [0.58, 0.85] |
| 1.8.3 Non RCT | 1 | 336 | Risk Ratio (M-H, Random, 95% CI) | 0.51 [0.27, 0.98] |

| 1.9 Duration to Recovery | 3 | 2020 | Mean Difference (IV, Random, 95% CI) | -4.77 [-12.54, 3.00] |
| --- | --- | --- | --- | --- |
| 1.10 Deterioration to SAM | 6 |  | Risk Ratio (M-H, Random, 95% CI) | Subtotals only |
| 1.10.1 Cluster RCT and RCT | 5 | 6788 | Risk Ratio (M-H, Random, 95% CI) | 0.87 [0.73, 1.03] |
| 1.10.2 Non-RCT | 1 | 336 | Risk Ratio (M-H, Random, 95% CI) | 0.61 [0.23, 1.60] |

| 1.11 Tranferred to Inpatient | 5 | 4939 | Risk Ratio (M-H, Random, 95% CI) | 0.57 [0.24, 1.34] |
| --- | --- | --- | --- | --- |
| 1.11.1 Cluster RCT | 2 | 1695 | Risk Ratio (M-H, Random, 95% CI) | 0.54 [0.07, 4.30] |
| 1.11.2 RCT | 3 | 3244 | Risk Ratio (M-H, Random, 95% CI) | 0.50 [0.31, 0.81] |
| 1.12 Mortality | 9 |  | Risk Ratio (M-H, Random, 95% CI) | Subtotals only |
| 1.12.1 Cluster RCT and RCT | 8 | 8934 | Risk Ratio (M-H, Random, 95% CI) | 0.91 [0.54, 1.52] |
| 1.12.2 Non-RCT | 1 | 336 | Risk Ratio (M-H, Random, 95% CI) | 0.56 [0.05, 6.08] |

| 1.13 Post Discharge Mortality | 3 | 2859 | Risk Ratio (M-H, Random, 95% CI) | 0.62 [0.28, 1.37] |
| --- | --- | --- | --- | --- |
| 1.13.1 Cluster RCT | 1 | 570 | Risk Ratio (M-H, Random, 95% CI) | 0.36 [0.13, 0.98] |
| 1.13.2 RCT | 2 | 2289 | Risk Ratio (M-H, Random, 95% CI) | 1.25 [0.16, 9.49] |
| 1.14 Weight Gain (g/kg/d) [g/kg/day] | 5 | 5054 | Mean Difference (IV, Random, 95% CI [g/kg/day]) | 0.62 [0.18, 1.06] |
| 1.14.1 Cluster RCT | 2 | 1939 | Mean Difference (IV, Random, 95% CI [g/kg/day]) | 0.49 [-0.69, 1.66] |
| 1.14.2 RCT | 3 | 3115 | Mean Difference (IV, Random, 95% CI [g/kg/day]) | 0.71 [0.24, 1.18] |

| 1.15 Weight Gain Total [kg] | 1 | 1264 | Mean Difference (IV, Random, 95% CI [kg]) | 0.23 [0.14, 0.32] |
| --- | --- | --- | --- | --- |
| 1.16 WHZ End | 3 | 5443 | Mean Difference (IV, Random, 95% CI) | 0.10 [0.05, 0.14] |
| 1.16.1 Cluster RCT | 1 | 1369 | Mean Difference (IV, Random, 95% CI) | 0.00 [-0.47, 0.47] |
| 1.16.2 RCT | 2 | 4074 | Mean Difference (IV, Random, 95% CI) | 0.11 [0.04, 0.17] |

| 1.17 WLZ Gain | 1 | 1264 | Mean Difference (IV, Random, 95% CI) | 0.28 [0.15, 0.41] |
| --- | --- | --- | --- | --- |
| 1.18 Length Gain | 1 | 1264 | Mean Difference (IV, Random, 95% CI) | 0.16 [-0.02, 0.34] |
| 1.18.1 Length Gain Total (cm) | 1 | 1264 | Mean Difference (IV, Random, 95% CI) | 0.16 [-0.02, 0.34] |

| 1.19 Length Gain (mm/d) | 2 | 4081 | Mean Difference (IV, Random, 95% CI) | -0.00 [-0.03, 0.02] |
| --- | --- | --- | --- | --- |
| 1.19.1 Cluster RCT | 1 | 1369 | Mean Difference (IV, Random, 95% CI) | -0.01 [-0.04, 0.02] |
| 1.19.2 RCT | 1 | 2712 | Mean Difference (IV, Random, 95% CI) | 0.01 [-0.03, 0.05] |
| 1.20 HAZ End | 2 | 2731 | Mean Difference (IV, Random, 95% CI) | 0.16 [-0.03, 0.34] |
| 1.20.1 Cluster RCT | 1 | 1369 | Mean Difference (IV, Random, 95% CI) | 0.20 [-0.29, 0.69] |
| 1.20.2 RCT | 1 | 1362 | Mean Difference (IV, Random, 95% CI) | 0.15 [-0.05, 0.35] |

| 1.21 MUAC Gain [cm] | 1 | 1264 | Mean Difference (IV, Random, 95% CI [cm]) | 0.29 [0.17, 0.41] |
| --- | --- | --- | --- | --- |
| 1.21.1 New Subgroup | 1 | 1264 | Mean Difference (IV, Random, 95% CI [cm]) | 0.29 [0.17, 0.41] |
| 1.22 MUAC Gain (mm/day) [mm/day] | 4 | 4474 | Mean Difference (IV, Random, 95% CI [mm/day]) | 0.04 [0.02, 0.06] |
| 1.22.1 Cluster RCT | 1 | 1369 | Mean Difference (IV, Random, 95% CI [mm/day]) | 0.04 [0.01, 0.07] |
| 1.22.2 RCT | 3 | 3105 | Mean Difference (IV, Random, 95% CI [mm/day]) | 0.04 [0.02, 0.07] |

| 1.23 Relapse after discharge | 2 | 2289 | Risk Ratio (M-H, Random, 95% CI) | 0.99 [0.82, 1.19] |
| --- | --- | --- | --- | --- |
| 1.24 Default Rate | 7 | 7570 | Risk Ratio (M-H, Random, 95% CI) | 1.32 [0.73, 2.40] |
| 1.24.1 Cluster RCT | 3 | 2964 | Risk Ratio (M-H, Random, 95% CI) | 2.59 [0.79, 8.53] |
| 1.24.2 RCT | 4 | 4606 | Risk Ratio (M-H, Random, 95% CI) | 0.88 [0.56, 1.40] |

| 1.25 Hemoglobin (Final) | 1 | 1154 | Mean Difference (IV, Random, 95% CI) | 0.25 [0.06, 0.44] |
| --- | --- | --- | --- | --- |
| 1.26 Change in Hemoglobin [gm/dL] | 2 | 1357 | Mean Difference (IV, Random, 95% CI [gm/dL]) | 0.36 [-0.34, 1.05] |
| 1.26.1 Cluster RCT | 1 | 1154 | Mean Difference (IV, Random, 95% CI [gm/dL]) | 0.72 [0.45, 0.99] |
| 1.26.2 RCT | 1 | 203 | Mean Difference (IV, Random, 95% CI [gm/dL]) | 0.01 [-0.15, 0.17] |

| 1.27 Vomiting | 1 | 2712 | Risk Ratio (M-H, Random, 95% CI) | 1.37 [1.09, 1.72] |
| --- | --- | --- | --- | --- |
| 1.28 Diarrhea | 1 | 2712 | Risk Ratio (M-H, Random, 95% CI) | 1.10 [0.98, 1.24] |
