# Supplementary material for: Lipid based nutrient supplements (LNS) for treatment of children (6 months to 59 months) with moderate acute malnutrition (MAM): A systematic review
Source: PLoS One. 2017 Sep 21;12(9):e0182096. doi: 10.1371/journal.pone.0182096 (PMC5608196; doi:10.1371/journal.pone.0182096)
Supplement: S9 Table — (DOCX) [file pone.0182096.s010.docx]

S9 Lipid-based nutrient supplements versus Counselling

| **Outcome or Subgroup** | **Studies** | **Participants** | **Statistical Method** | **Effect Estimate** |
| --- | --- | --- | --- | --- |

| 2.1 Recovery from MAM | 1 | 1299 | Risk Ratio (M-H, Random, 95% CI) | 1.28 [1.18, 1.39] |
| --- | --- | --- | --- | --- |
| 2.2 No Recovery | 1 | 1299 | Risk Ratio (M-H, Random, 95% CI) | 0.94 [0.68, 1.28] |

| 2.3 Deterioration to SAM | 1 | 1299 | Risk Ratio (M-H, Random, 95% CI) | 0.71 [0.51, 0.99] |
| --- | --- | --- | --- | --- |
| 2.4 Mortality | 1 | 1299 | Risk Ratio (M-H, Random, 95% CI) | 0.44 [0.11, 1.74] |

| 2.5 Defaulter | 1 | 1299 | Risk Ratio (M-H, Random, 95% CI) | 0.37 [0.26, 0.51] |
| --- | --- | --- | --- | --- |
| 2.6 Weight Gain | 1 | 1299 | Mean Difference (IV, Random, 95% CI) | 0.50 [0.18, 0.82] |
| 2.6.1 Weight Gain (g/kg/d) | 1 | 1299 | Mean Difference (IV, Random, 95% CI) | 0.50 [0.18, 0.82] |

| 2.7 WHZ End | 1 | 1299 | Mean Difference (IV, Random, 95% CI) | 0.20 [-0.13, 0.53] |
| --- | --- | --- | --- | --- |
| 2.8 Length Gain | 1 | 1299 | Mean Difference (IV, Random, 95% CI) | 0.02 [-0.00, 0.04] |
| 2.8.1 Length Gain (mm/d) | 1 | 1299 | Mean Difference (IV, Random, 95% CI) | 0.02 [-0.00, 0.04] |

| 2.9 HAZ End | 1 | 1299 | Mean Difference (IV, Random, 95% CI) | 0.50 [0.14, 0.86] |
| --- | --- | --- | --- | --- |
| 2.10 MUAC Gain | 1 | 1299 | Mean Difference (IV, Random, 95% CI) | 0.02 [-0.02, 0.06] |
| 2.10.1 MUAC Gain (mm/d) | 1 | 1299 | Mean Difference (IV, Random, 95% CI) | 0.02 [-0.02, 0.06] |
