# Supplementary material for: Lipid based nutrient supplements (LNS) for treatment of children (6 months to 59 months) with moderate acute malnutrition (MAM): A systematic review
Source: PLoS One. 2017 Sep 21;12(9):e0182096. doi: 10.1371/journal.pone.0182096 (PMC5608196; doi:10.1371/journal.pone.0182096)
Supplement: S10 Table — (DOCX) [file pone.0182096.s011.docx]

## S10 Sensitivity Analyses for Recovery from Moderate Acute Malnutrition

| **Outcome or Subgroup** | **Studies** | **Participants** | **Statistical Method** | **Effect Estimate** |
| --- | --- | --- | --- | --- |

| 3.1 Sequence Generation | 9 | 9270 | Risk Ratio (M-H, Random, 95% CI) | 1.09 [1.03, 1.15] |
| --- | --- | --- | --- | --- |
| 3.1.1 Low Risk | 8 | 8934 | Risk Ratio (M-H, Random, 95% CI) | 1.08 [1.02, 1.14] |
| 3.1.2 Others | 1 | 336 | Risk Ratio (M-H, Random, 95% CI) | 1.23 [1.06, 1.42] |
| 3.2 Allocation Concealment | 9 | 9270 | Risk Ratio (M-H, Random, 95% CI) | 1.09 [1.03, 1.15] |
| 3.2.1 Low Risk | 5 | 5731 | Risk Ratio (M-H, Random, 95% CI) | 1.10 [1.03, 1.18] |
| 3.2.2 Others | 4 | 3539 | Risk Ratio (M-H, Random, 95% CI) | 1.08 [0.97, 1.20] |

| 3.3 Blinding of Participants and Personnel | 9 | 9270 | Risk Ratio (M-H, Random, 95% CI) | 1.09 [1.03, 1.15] |
| --- | --- | --- | --- | --- |
| 3.3.1 Low Risk | 3 | 4155 | Risk Ratio (M-H, Random, 95% CI) | 1.06 [0.99, 1.14] |
| 3.3.2 Others | 6 | 5115 | Risk Ratio (M-H, Random, 95% CI) | 1.10 [1.01, 1.20] |
| 3.4 Blinding of Outcome Assessment | 9 | 9270 | Risk Ratio (M-H, Random, 95% CI) | 1.09 [1.03, 1.15] |
| 3.4.1 Low Risk | 2 | 2793 | Risk Ratio (M-H, Random, 95% CI) | 1.04 [0.96, 1.12] |
| 3.4.2 Others | 7 | 6477 | Risk Ratio (M-H, Random, 95% CI) | 1.10 [1.02, 1.18] |

| 3.5 Incomplete Outcome Data | 9 | 9270 | Risk Ratio (M-H, Random, 95% CI) | 1.09 [1.03, 1.15] |
| --- | --- | --- | --- | --- |
| 3.5.1 Low Risk | 8 | 8934 | Risk Ratio (M-H, Random, 95% CI) | 1.08 [1.02, 1.14] |
| 3.5.2 Others | 1 | 336 | Risk Ratio (M-H, Random, 95% CI) | 1.23 [1.06, 1.42] |
| 3.6 Selective Outcome Reporting | 9 | 9270 | Risk Ratio (M-H, Random, 95% CI) | 1.09 [1.03, 1.15] |
| 3.6.1 Low Risk | 9 | 9270 | Risk Ratio (M-H, Random, 95% CI) | 1.09 [1.03, 1.15] |
| 3.6.2 Others | 0 | 0 | Risk Ratio (M-H, Random, 95% CI) | Not estimable |

| 3.7 Other Bias | 9 | 9270 | Risk Ratio (M-H, Random, 95% CI) | 1.09 [1.03, 1.15] |
| --- | --- | --- | --- | --- |
| 3.7.1 Low Risk | 7 | 7694 | Risk Ratio (M-H, Random, 95% CI) | 1.07 [1.01, 1.13] |
| 3.7.2 Others | 2 | 1576 | Risk Ratio (M-H, Random, 95% CI) | 1.15 [1.04, 1.28] |
